# Cytotoxic Minor Piericidin Derivatives from the Actinomycete Strain *Streptomyces* *psammoticus* SCSIO NS126

**DOI:** 10.3390/md19080428

**Published:** 2021-07-28

**Authors:** Kunlong Li, Ziqi Su, Yongli Gao, Xiuping Lin, Xiaoyan Pang, Bin Yang, Huaming Tao, Xiaowei Luo, Yonghong Liu, Xuefeng Zhou

**Affiliations:** 1 CAS Key Laboratory of Tropical Marine Bio-Resources and Ecology, Guangdong Key Laboratory of Marine Materia Medica, South China Sea Institute of Oceanology, Chinese Academy of Sciences, Guangzhou 510301, China; likunlong16@mails.ucas.ac.cn (K.L.); xiupinglin@hotmail.com (X.L.); xypang@scsio.ac.cn (X.P.); yangbin@scsio.ac.cn (B.Y.); 2 Southern Marine Science and Engineering Guangdong Laboratory (Guangzhou), Guangzhou 511458, China; yongligao@scsio.ac.cn; 3 College of Earth and Planetary Sciences, University of Chinese Academy of Sciences, Beijing 100049, China; 4 School of Traditional Chinese Medicine, Southern Medical University, Guangzhou 510515, China; ziqisl@163.com (Z.S.); taohm@smu.edu.cn (H.T.); 5 Institutional Center for Shared Technologies and Facilities, South China Sea Institute of Oceanology, Chinese Academy of Sciences, Guangzhou 510301, China; 6 Institute of Marine Drugs, Guangxi University of Chinese Medicine, Nanning 530200, China

**Keywords:** actinomycete, piericidins, absolute configuration, cytotoxicity

## Abstract

The mangrove-sediment-derived actinomycete strain *Streptomyces* *psammoticus* SCSIO NS126 was found to have productive piericidin metabolites featuring anti-renal cell carcinoma activities. In this study, in order to explore more diverse piericidin derivatives, and therefore to discover superior anti-tumor lead compounds, the NS126 strain was further fermented at a 300-L scale under optimized fermentation conditions. As a result, eight new minor piericidin derivatives (piericidins L-R (**1**–**7**) and 11-demethyl-glucopiericidin A (**8**)) were obtained, along with glucopiericidin B (**9**). The new structures including absolute configurations were determined by spectroscopic methods coupled with experimental and calculated electronic circular dichroism. We also proposed plausible biosynthetic pathways for these unusual post-modified piericidins. Compounds **1** and **6** showed selective cytotoxic activities against OS-RC-2 cells, and **2**–**5** exhibited potent cytotoxicity against HL-60 cells, with IC_50_ values lower than 0.1 μM. The new piericidin glycoside **8** was cytotoxic against ACHN, HL-60 and K562, with IC_50_ values of 2.3, 1.3 and 5.5 μM, respectively. The ability to arrest the cell cycle and cell apoptosis effects induced by **1** and **6** in OS-RC-2 cells, **2** in HL-60 cells, and **8** in ACHN cells were then further investigated. This study enriched the structural diversity of piericidin derivatives and confirmed that piericidins deserve further investigations as promising anti-tumor agents.

## 1. Introduction

Actinomycetes from terrestrial sources have been studied and screened since the 1950s, yielding many important anti-infective and anti-cancer drugs. At the same time, actinomycetes isolated from the marine environment, especially *Streptomyces* strains, have yielded several promising drug candidates, and have received considerable attention in recent years [1,2,3]. Piericidins, which are produced by actinomycetes isolated from soil, insects and marine samples, are known as NADH:ubiquinone oxidoreductase inhibitors and antitumor agents [4].

Recently, some biologically significant piericidins were discovered in two mangrove-sediment-derived *Streptomyces* strains by our group [5,6,7]. In a culture extract of the strain *Streptomyces psammoticus* SCSIO NS126, twenty-seven natural piericidins were isolated and found to have notable anti-renal cell carcinoma (RCC) activities. Our previous studies also revealed that piericidins could conceivably provide a novel scaffold for the further development of potent and mechanistically novel anti-RCC agents [5,6]. It was previously shown that external pH plays an important role in regulating the secondary metabolites of microorganisms [8]. At times, the influence of pH was found to be greater than that of carbon and nitrogen nutrition in the cultivation of bacteria [9]. In recent years, bacteria capable of growing under acidic conditions have attracted the interest of scientists due to their structurally novel secondary metabolites featuring compelling biological activities [10]. Researchers have demonstrated that mangrove-sediment microbial communities are highly correlated with environmental factors such as salinity, pH and nutrient bioavailability [11]. 

**Figure 1:**
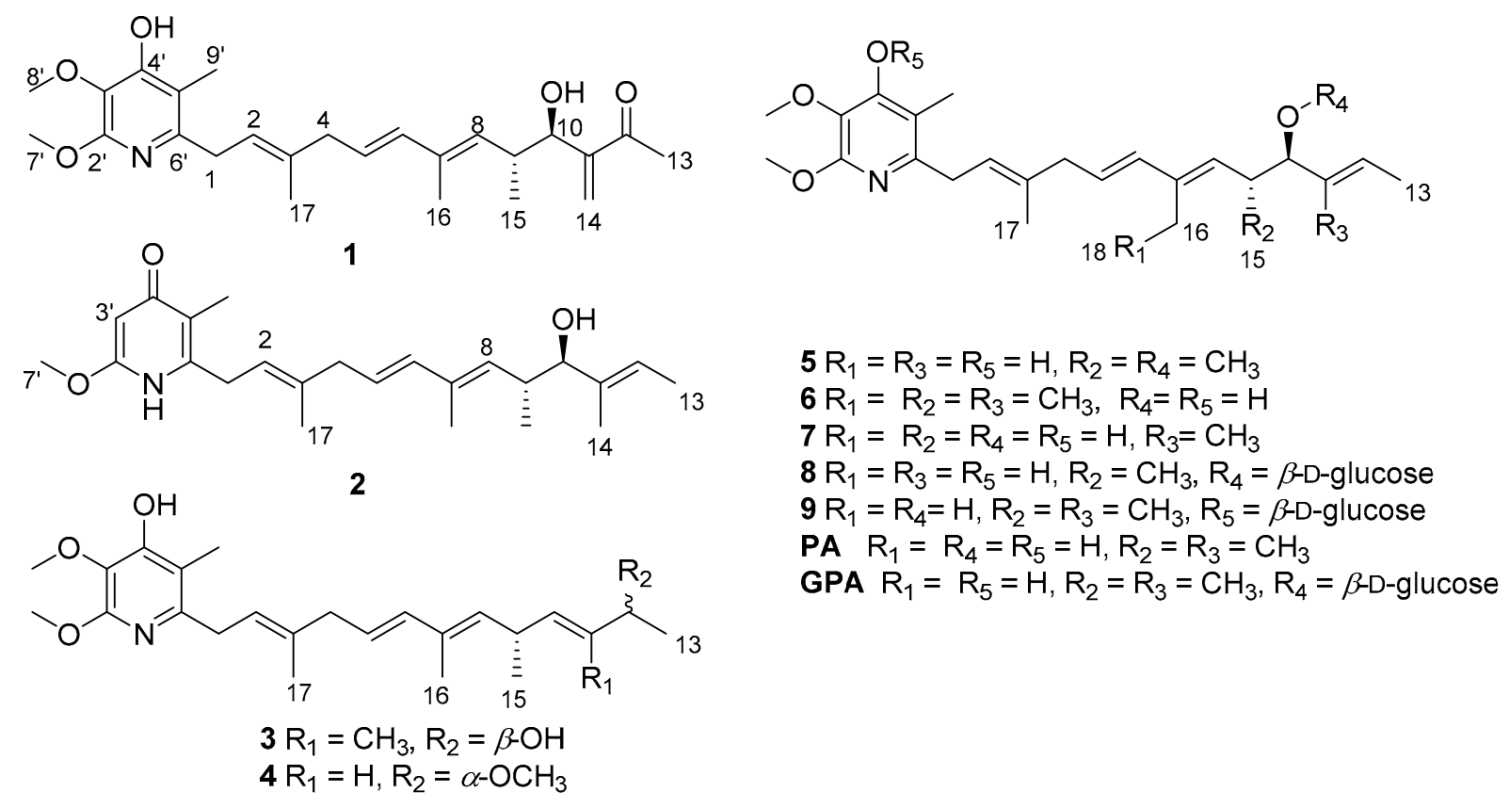
Structures of compounds **1**–**9**, PA and GPA.

In order to explore more diverse piericidin derivatives, and therefore to discover better anti-tumor lead compounds, we sought to optimize the fermentation conditions of the mangrove-sediment-derived NS126 strain by adjusting the pH and choosing the culture time, as well as engaging in large-scale fermentation (300 L). In addition to the isolation and accumulation of the reported active piericidins, nine other piericidin compounds (**1**–**9**) were obtained (Figure 1), including eight new piericidins (**1**–**8**) in trace amounts (about 1–3 mg), as well as a known glucopireicidin B (**9**) that was first discovered in this strain. Herein, we report the isolation, structural elucidation and bioactivities of these compounds.

## 2. Results

In the fermentation condition screening tests, the secondary metabolites of the NS126 strain fermented at pH 4 showed greater chemical diversity (Figure S82). Moreover, after culturing for 30 h (pH 4), the strain was able to produce a large quantity of the main piericidin metabolites (Figure S83), piericidin A (PA) and glucopiericidin A (GPA) (Figure 1) [6]. At the same time, the extract also showed a variety of chemicals in its composition (Figure S84). Strain NS126 was further fermented at a 300 L scale under optimized fermentation conditions (pH 4, cultured for 30 h). About 1200 mg PA and 680 mg GPA were isolated and purified in order to further evaluate their pharmacological and pharmacokinetic/toxicokinetic properties [5]. In addition to the accumulation of the reported active piericidins, eight other new piericidins (**1**–**8**) were obtained in trace amounts (about 1–3 mg), as well as a known glucopireicidin B (**9**) that was discoved first in this strain [12] (Figure 1).

**Figure 2:**
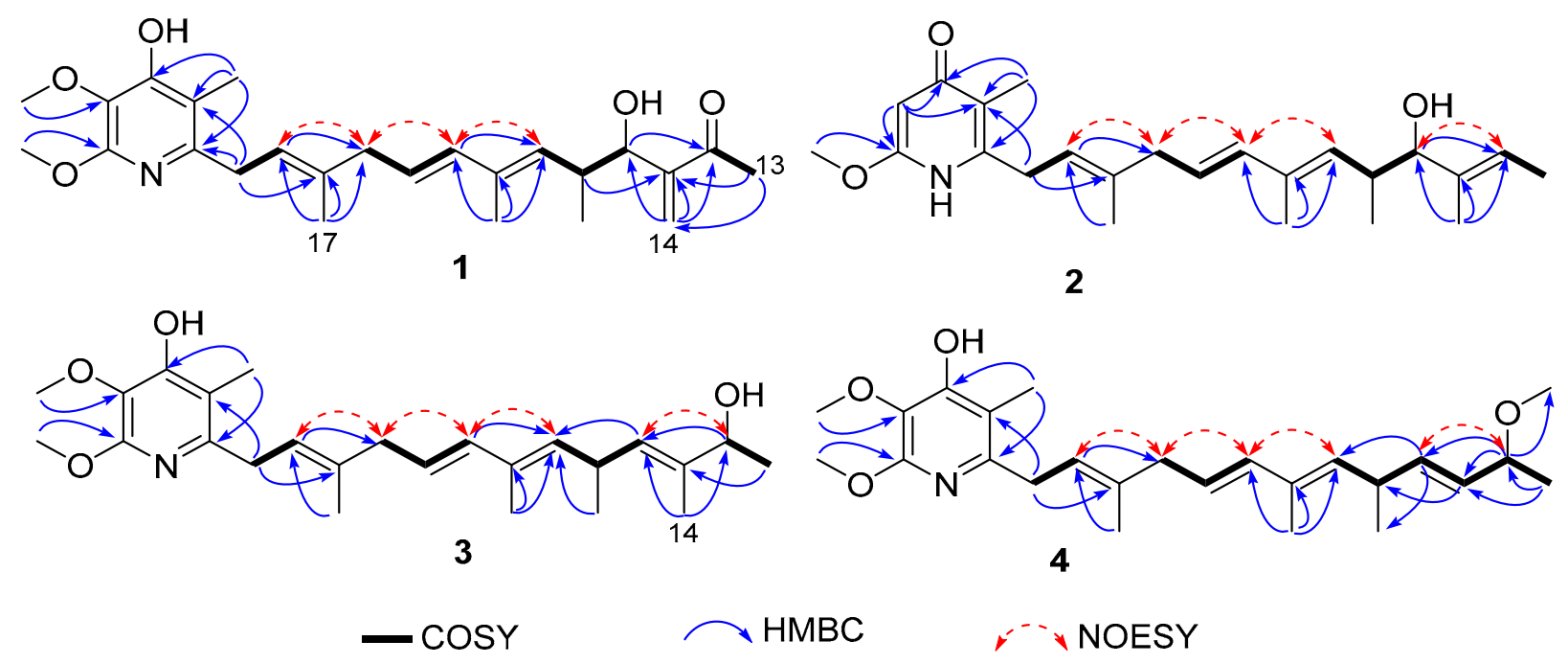
Key COSY, HMBC and NOESY correlations in **1**–**4**.

Compound **1** was isolated as a pale yellow oil. The HRESIMS (*m*/*z* 430.2583 [M + H]^+^) data suggested the molecular formula of C_25_H_35_NO_5_, revealing nine degrees of unsaturation. The IR spectrum exhibited absorption bands for the pyridine ring (1585, 1500 and 802 cm^−1^) and one carbonyl group (1670 cm^−1^). The NMR spectra showed signals for seven methyls (including two oxygenated ones), three methylenes (including one terminal olefinic carbon), six methines (including four olefinic ones) and nine aromatic/olefinic carbons. A comparison of the pyridine ring’s ^1^H and ^13^C NMR data (Table 1 and Table 2) with those of the PA indicated that they share a similar skeleton [13]. However, a set of signals at H_2_-14 (*δ*_H_ 6.21; *δ*_H_ 6.02), C-11 (*δ*_C_ 152.0) and C-12 (*δ*_C_ 201.8) (Table 1 and Table 2), which was not present in the ^1^H and ^13^C NMR spectra of PA, was observed in the spectrum of **1**. Through the analysis of the HMBC spectrum, the *α*, *β*-unsaturated carbonyl moiety (CH_2_=C-C=O) was confirmed by the HMBC correlations from H_2_-14 to C-10/C-11/C-12, H_3_-13 to C-11/C-14 and H-10 to C-12 (Figure 2). In this way, the planar structure of **1**, named piericidin L (**1**), was identified, as shown in Figure 1. Piericidin L is the first piericidin analogue possessing a terminal-olefinic-bond moiety conjugated with the carbonyl moiety [6].

Compound **2** was obtained as a brown oil, and it exhibited a molecular formula of C_24_H_35_NO_3_ via the (+)-HRESIMS ion at *m*/*z* 386.2696 [M + H]^+^. The NMR signals of **2** resembled those of Mer-A2026 B, except for the presence of another carbonyl group at C-4′ (*δ*_C_ 179.1), which was confirmed by the HMBC correlations of H-3′ with C-2′/C-4′/C-5′, and those of H_3_-9′ with C-4′/C-5′ (Figure 2) [14]. In this way, the planar structure of **2** was established (Figure 1) and named piericidin N (**2**), which is the third piericidin aglycone found to consist of a pyridone structure [15,16].

Compound **3** was isolated as a pale yellow oil. The (+)-HRESIMS data (*m*/*z* 438.2614 [M + Na]^+^) indicated that this compound possessed the same molecular formula as PA—C_25_H_37_NO_4_. A comparison of the ^1^H and ^13^C NMR data for **3** and PA suggested that **3** was an analogue of PA, with a pyridine, four double bonds, and two hydroxyl groups (Table 1 and Table 2) [13]. However, the locations of C-10 (*δ*_C_ 130.3) and C-12 (*δ*_C_ 73.9) changed, as determined by the ^1^H-^1^H COSY signals of two isolated proton-spin systems of H-8/H-9/H-10 (*δ*_H_ 5.26) and H-12 (*δ*_H_ 4.11)/H_3_-13, together with the HMBC correlations of H-10 with C-8/C-9, H_3_-13 with C-11/C-12, H_3_-14 with C-10/C-12, and H_3_-15 with C-8/C-9/C-10 (Figure 2). In this way, the planar structure of **3** was determined and named piericidin Q (**3**), as shown in Figure 1.

Compound **4** was determined to have the same molecular formula as **3**. A comparison of the ^1^H and ^13^C NMR spectra of **4** with those of **3** showed many similarities, except for the elimination of 11-CH_3_ and the methylation of 12-OH (Table 1 and Table 2), which were confirmed by the ^1^H-^1^H COSY correlations of H-8/H-9/H-10/H-11/H-12/H-13, as well as the HMBC correlations of H-10 to C-8/C-9, H-11 to C-9, H-12 to C-11/C-12, and H_3_-14 to C-12 (Figure 2). Compound **4** was named piericidin O (**4**), as shown in Figure 1. Both **3** and **4** are the first-discovered piericidin analogues with changes in C-10 and C-12, representing novel post-modification piericidins.

Compound **5** displayed a hydrogen adduct ion at *m*/*z* 416.2808 [M + H]^+^ in its (+)-HRESIMS data, which determined the molecular formula of C_25_H_37_NO_4_. The analysis of the ^1^H and ^13^C NMR data indicated that **5** was structurally close to 11-demethylpiericidin A, except that 10-OH was changed to a methoxyl group in **5** [6]. The above data were supported by the HMBC correlations of H_3_-14 with C-10 and H-10 with C-8/C-15/C-12, as well as the ^1^H-^1^H COSY correlations of H-8/H-9/H-10/H-11/H-12/H-13. The ultimate planar structure of **5**, piericidin P (**5**), is shown in Figure 1. 

Compounds **6** and **7** were both isolated as a faint yellow oil, and their molecular formulae were established as C_26_H_39_NO_4_ and C_24_H_35_NO_4_, according to the HRESIMS data ((*m*/*z* 430.2955 [M + H]^+^) and (*m*/*z* 402.2640 [M + H]^+^), respectively). A comparison of their ^1^H and ^13^C NMR data with those of PA indicated that both **6** and **7** shared a similar PA skeleton (Table 1 and Table 2) [13]. For **6**, the only change was the replacement of 7-CH_3_ in the PA with ethyl (16-CH_2_, 18-CH_3_), which was corroborated by the HMBC correlations from H_3_-18 to C-7 and H_2_-16 to C-6/C-7/C-8, as well as the ^1^H-^1^H COSY correlations of H_3_-18/H_2_-16. Similarly, the only difference in **7** was the disappearance of 9-CH_3_ in PA, which was confirmed by the ^1^H-^1^H COSY correlations (H-8/H_2_-9/H-10) and HMBC correlations (H_2_-9 to C-7/C-11). The planar structures of **6** and **7**, named piericidins M (**6**) and R (**7**), were ultimately determined as shown in Figure 1.

Compound **8** was obtained as a pale yellow oil with a molecular formula of C_30_H_45_NO_9_, as determined by (+)-HRESIMS (*m*/*z* 564.3162 [M + H]^+^). The ^1^H and ^13^C NMR data were similar to those of glucopiericidin A, except for the absence of 11-CH_3_ (Table 1 and Table 2), which was confirmed by the HMBC correlations of H-10 with C-8/C-15/C-12 and the ^1^H-^1^H COSY correlations of H-8/H-9/H-10/H-11/H-12/H-13 [12]. The connection between the hexose unit and C-10 was established by the HMBC correlation of the anomeric proton H-1″ (*δ*_H_ 4.33) with C-10 (*δ*_C_ 86.1). After the acidic hydrolysis of **8**, d-glucose was identified by reversed-phase HPLC (26.78 min). The coupling constant of the anomeric proton at *δ*_H_ 4.33 (*J* = 7.8 Hz) indicated it to be a *β*-glucoside [17]. The planar structure of **8** was thus established as 11-demethyl-glucopiericidin A (**8**), as shown in Figure 1. 

The geometries of the olefins in compounds **1**–**8** were all deduced to be *E*, based on the NOESY correlations of H-2/H_2_-4, H_2_-4/H-6, H-6/H-8 and H-10/H-12 (Figure 2).

**Figure 3:**
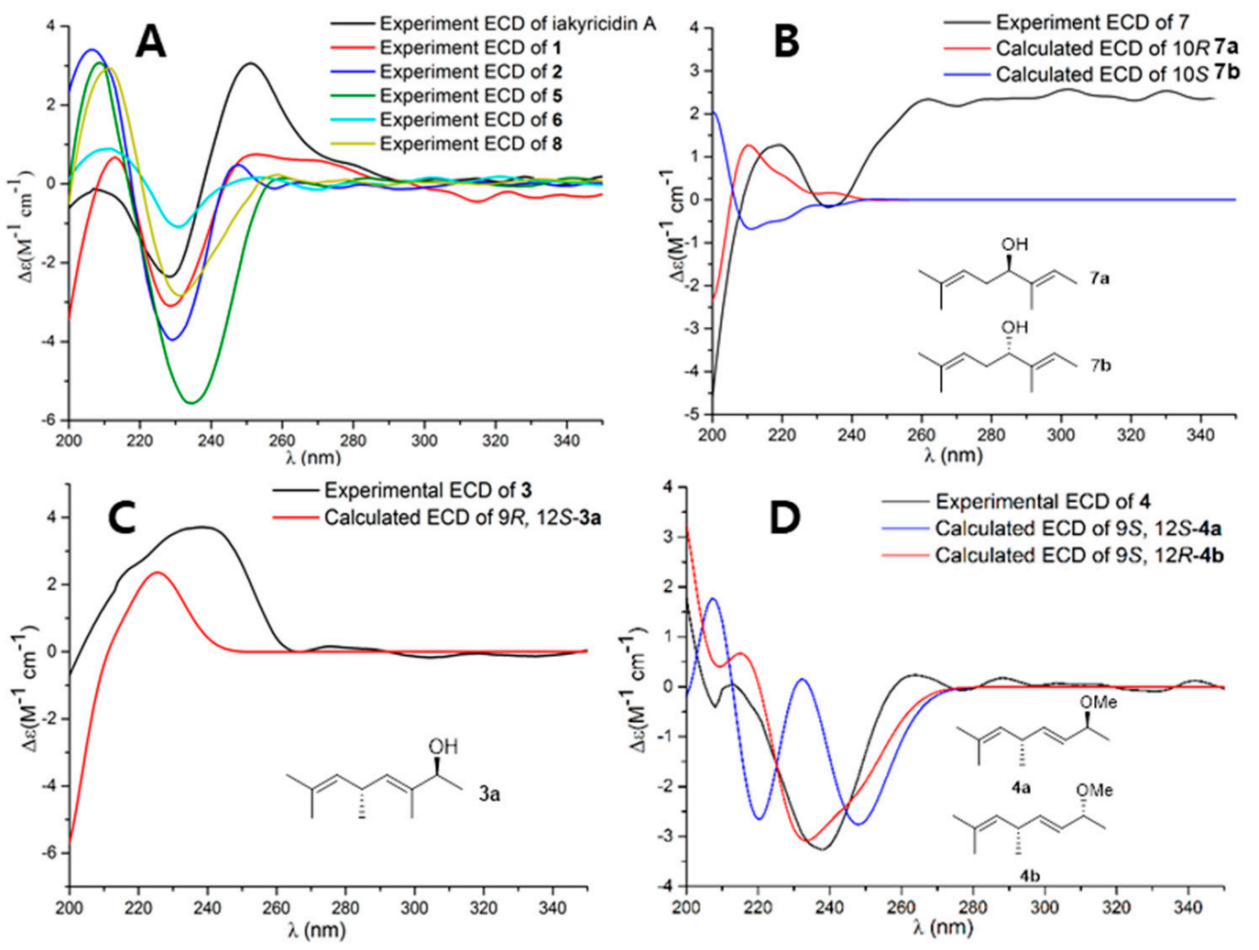
(**A**) Experimental ECD spectra of **1**–**2**, **5**–**6**, **8**, and iakyricidin A. (**B**) Experimental ECD spectra of **7,** and the calculated ECD spectra of truncated models **7a**/**7b**. (**C**) Experimental ECD spectra of **3,** and the calculated ECD spectrum of truncated model **3a**. (**D**) Experimental ECD spectra of **4,** and the calculated ECD spectra of truncated models **4a**/**4b**.

Considering a biosynthetic point of view, the nearly identical experimental ECD curves between compounds **1**–**2**, **5**–**6**, **8** and the reported iakyricidin A revealed the shared 9*R* and 10*R* configurations in the five compounds (Figure 3A) [7]. Moreover, the Boltzmann-weighted ECD spectra of the truncated model 10*R*-**7a** gave the best agreement with the experimental ECD spectrum of **7** (Figure 3B) and facilitated the determination of the 10*R* absolute configuration of **7**, which was consistent with that of the biosynthetic piericidin analogues [18].

**Figure 4:**
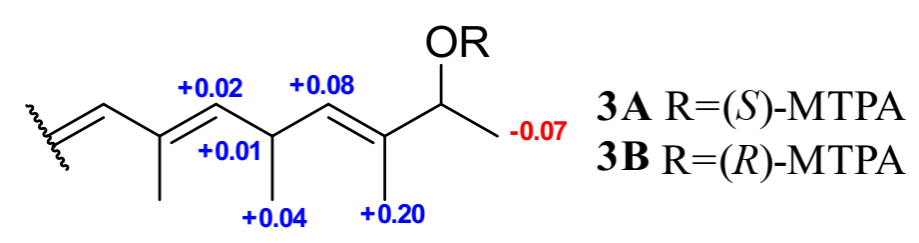
Δ *δ*_H_ values (*δ_S_*−*δ_R_*, in ppm) for **3A** and **3B**.

Mosher’s method was employed to determine the absolute configurations of C-12 in **3**. The treatment of **3** with (*R*)- and (*S*)- MTPA-Cl yielded (*S*)- and (*R*)- MTPA ester derivatives, respectively. The calculation of the ^1^H NMR Δ *δ*_S−R_ values for the *mono*-MTPA esters of **3** established the 12*S* configuration (Figure 4) [7]. Moreover, given that **3** was a post-modified derivative of PA, the absolute configuration of C-9 was inferred as *R*, which was supported by the similarity between the calculated ECD spectrum of truncated models 9*R* and 12*S*-**3a** and the experimental ECD of **3**, as shown in Figure 3C. Finally, the absolute configurations of **3** were assigned as (9*R*, 12*S*) [18].

From a biosynthetic point of view, and considering **4** as post-modified piericidin, the absolute configuration of C-9 was determined as *S*. Due to the opposite cotton effect that occurred at 240 nm between **3** and **4**, the absolute configuration of C-12 in **4** was inferred to be *R*. Furthermore, the ECD spectra of the truncated models 12R*-**4a**/**4b** were calculated in order to determine the absolute configurations. The results indicated that the experimental ECD spectra exhibited similar cotton effects to those of the spectra calculated for 12*R*-**4b,** as shown in Figure 3D [18]. Accordingly, the absolute configuration of **4** was established as (9*S*, 12*R*).

The known glucopiericidin B (**9**) was identified by the comparison of its ^1^H and ^13^C NMR data with the literature data [12]. This is the first time that glucopiericidin B (**9**) was obtained from this strain.

**Scheme 1:**
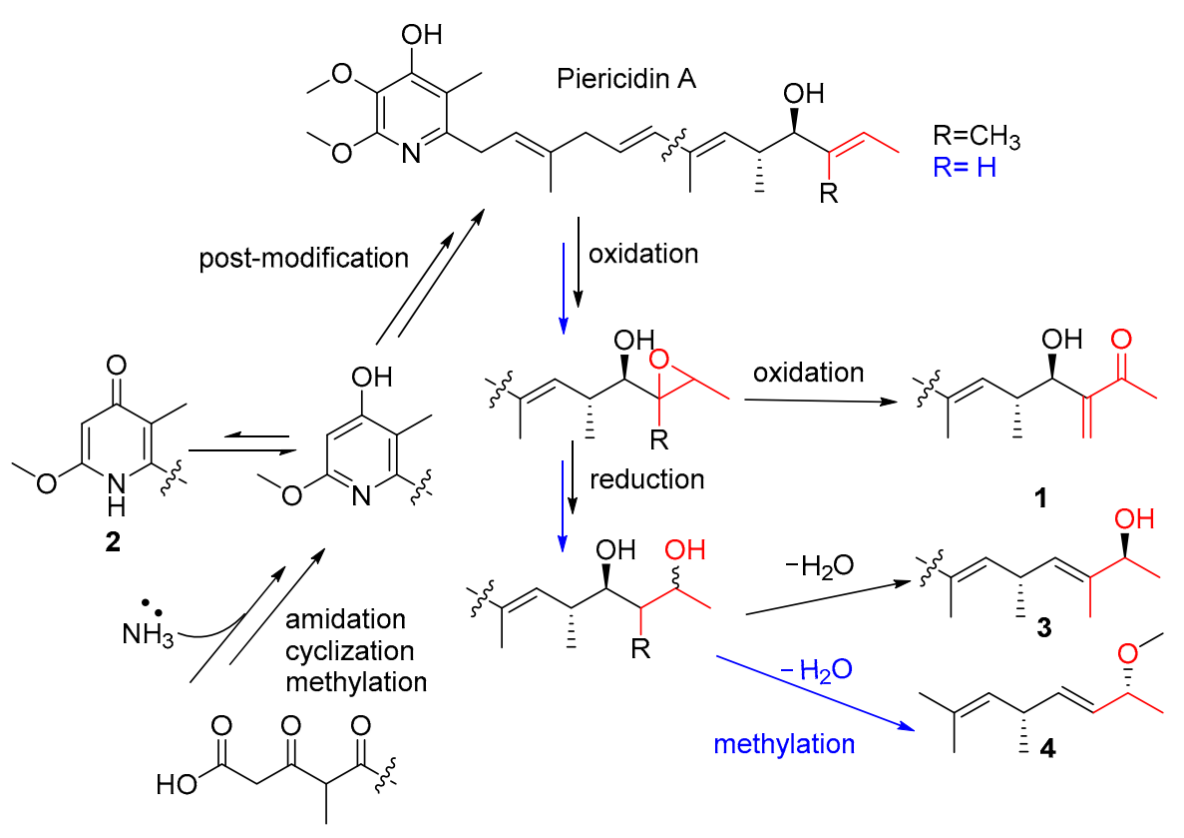
Plausible biogenetic pathways.

In the biosynthetic pathways, piericidins originate from a modular polyketide synthase (PKS) pathway [16,19]. Compounds **1**, **3** and **4** represented three unusual post-modified piericidins, all of which might be biogenetically derived from the main metabolite, PA/11-demethylpiericidin A. Compound **1** was formed from PA through two-step oxidation. Compounds **3** and **4** were derived from PA/11-demethylpiericidin A through oxidation, hydrogenation reduction, dehydration and methylation (Scheme 1). Compounds **6** and **7** were much more likely to be biogenetically derived from the main metabolite, piericidin A, by unfrequent methylation on C-16 (for **6**) or demethylation on C-15 (for **7**). Unlike the previously reported compounds of this strain, the plausible post-PKS modification steps have never been reported in the corresponding positions of piericidins. Some authors reported special piericidin analogues with branched-chain C–C cyclization [7] and 13-dimethoxy and C-2/C-3 epoxy rings [6,16]; these rare analogues indicated several plausible special post-PKS modifications in piericidin biosynthesis.

Due to the previously discovered potential of piericidins in treating RCC [6], eight new piericidins were evaluated for their antiproliferative activities against three human renal cancer cell lines—ACHN, 786-O, and OS-RC-2—together with three human leukemia cell lines: HL-60, K-562 and MOLT-4. As presented in Table 3, piericidins L (**1**) and M (**6**) showed selective anti-proliferative activities against OS-RC-2 cells, with IC_50_ values of 2.2 and 4.5 μM, respectively. Compounds **2**–**5** exhibited strong inhibition against HL-60 cells, with IC_50_ values lower than 0.1 μM. The new piericidin glycoside, 11-demethyl-glucopiericidin A (**8**), displayed significant cytotoxicities against ACHN, HL-60 and K562, with IC_50_ values of 2.3, 1.3 and 5.5 μM, respectively. 

**Figure 5:**
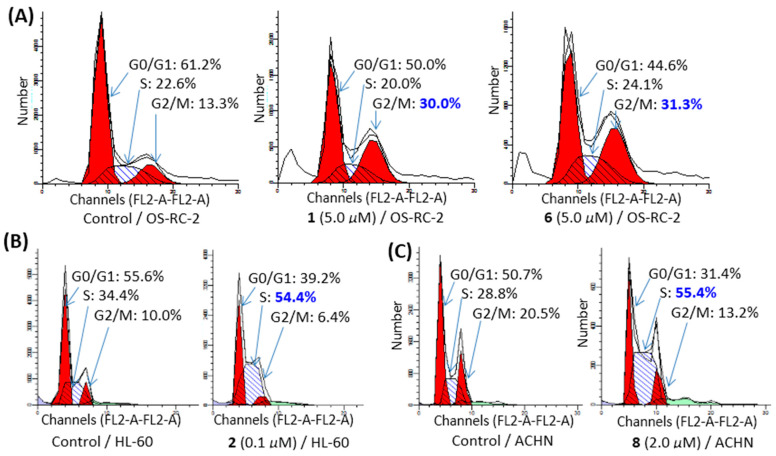
(**A**) **1**- and **6**-arrested cell cycle progression during the G2/M phase in OS-RC-2 cells; (**B**) **2**-arrested cell cycle progression during the S phase in HL-60 cells; (**C**) **8**-arrested cell cycle progression during the S phase in ACHN cells.

**Figure 6:**
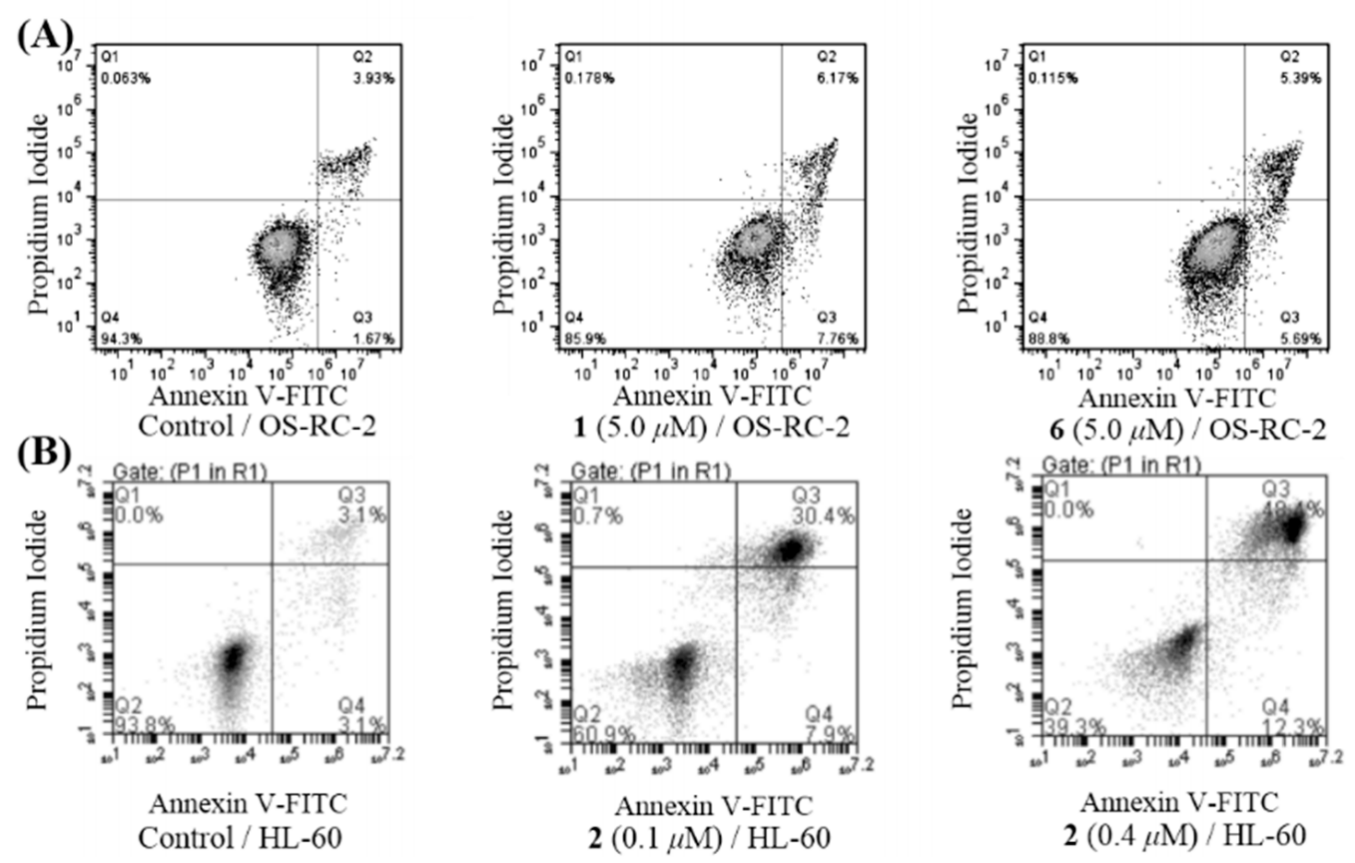
(**A**) **1**- and **6**-induced apoptosis in OS-RC-2 cells; the OS-RC-2 cells were treated with **1** or **6** (5 μM) for 24 h. (**B**) **2**-induced apoptosis in HL-60 cells; the HL-60 cells were treated with **2** (0.1 and 0.4 μM) for 72 h.

Due to the compounds’ selective activities against cancer cells [7], we further investigated the abilities of **1** and **6**, **2**, and **8** to arrest the cell cycle and induce cell apoptosis effects in OS-RC-2 cells, HL-60 cells and ACHN cells, respectively. In order to determine whether an alteration of the cell cycle occurred after treatment with these compounds, the DNA content of the treated cells was measured by flow cytometry. The results indicated that **1** and **6** (with concentrations of 5 μM for both) could arrest the cell cycle during the G2/M phase in OS-RC-2 cells (Figure 5A), while **2** (0.1 μM, Figure 5B) and **8** (2 μM, Figure 5C) could arrest the cell cycle during the S phase in HL-60 and ACHN cells. The apoptotic cells induced by **1**, **2**, or **6** were quantified by flow cytometry using Annexin V (AV)-FITC/propidium iodide (PI) double staining. The results showed that **1** and **6** (both 5 μM) could induced apoptosis after a 24 h treatment of OS-RC-2 cells (Figure 6A), while **2** (0.1 and 0.4 μM) could induce apoptosis after a 72 h treatment of HL-60 cells (Figure 6B).

## 3. Discussion

According to our previous review, 40 natural piericidin derivatives were reported before 2016, including 11 piericidin glycosides [4]. In order to explore the natural piericidin compounds from Actinomycetes, our group performed chemical dereplication using high-performance liquid chromatography/mass spectrometry (HPLC/MS) to screen the strains with piericidins in marine (including mangrove-derived) *Streptomyces* strains [6]. The HPLC/MS analysis uncovered abundant and diverse piericidins in two actinomycete strains, *S. iakyrus* SCSIO NS104 and *S. psammoticus* SCSIO NS126, isolated from a mangrove sediment sample collected from the Pearl River estuary in the South China Sea. The chemical investigation of the NS104 and NS126 strains revealed 34 natural piericidins, including 21 new compounds [6,7]. These two actinomycete strains, especially NS126, have made important contributions and provided both new and diverse natural piericidin derivatives. Although the recent literature showed that biosynthesis provides a novel way to produce piericidin derivatives [20], the optimization of the fermentation conditions of the target strain remains the most effective way to obtain target compounds with similar structures. In this paper, the NS126 strain was further optimized and subjected to a 300 L fermenter, which resulted in the isolation of eight unusual post-modified piericidin derivatives (**1**–**8**) in trace amounts under acidic conditions. The bioassay showed the carbonyl-containing piericidin L (**1**) with selective cytotoxic activities, which was similar to the previous reported carbonyl-containing piericidin analog iakyricidin A [7]. It is suggested that a carbonylated branched chain could greatly enhance the cytotoxicity of the piericidin analogs. The new piericidin glycoside **8** has a broad spectrum of cytotoxic activity, and it also confirmed that piericidin glycosides possess the potential for further development as antitumor agents [5,6]. This study advances our comprehensive understanding of the structural diversity and cytotoxicity of actinomycete-derived piericidin compounds.

## 4. Materials and Methods

### 4.1. General Experimental Procedures

The optical rotations were achieved using a PerkinElmer MPC 500 (Waltham) polarimeter. The UV spectra were recorded on a Shimadzu UV-2600 PC spectrometer (Shimadzu). The ECD spectra were measured using a Chirascan circular dichroism spectrometer (Applied Photophysics). The IR spectra were measured on an IR Affinity-1 spectrometer (Shimadzu, Beijing, China). The NMR spectra were obtained on a Bruker Avance spectrometer (Bruker) operating at 700 MHz for ^1^H NMR and 175 MHz for ^13^C NMR, using tetramethylsilane as an internal standard. The HRESIMS spectra were collected on a Bruker mix TOF-QII mass spectrometer (Bruker). The TLC and column chromatography (CC) were performed on plates pre-coated with GF254 silica gel (10−40 μm) and over silica gel (200−300 mesh) (Qingdao Marine Chemical Factory) and a Sephadex LH-20 (Amersham Biosciences), respectively. All of the solvents employed were of analytical grade (Tianjin Fuyu Chemical and Industry Factory). The semipreparative HPLC was performed on an HPLC (Hitachi-L2130, diode array detector, Hitachi L-2455, Tokyo, Japan) using a Phenomenex ODS column (250 mm × 10.0 mm i.d., 5 μm; Phenomenex, Torrance, CA, USA). The artificial sea salt was a commercial product (Guangzhou Haili Aquarium Technology Company, Guangzhou, China).

### 4.2. Collection of THE Information on, and Cultivation of, the NS126 Strain

The information on the NS126 strain was reported in our previous study [6]. A few cell-loops of the strains were inoculated into a 1000 mL Erlenmeyer flask containing 100 mL seed medium (mannitol 1 g, soya peptone 0.5 g, soya-bean oil 0.125 g, K2HPO3 0.02 g, pH 7.0 and 50 mL distilled water) and then cultivated on a rotary shaker at 180 rpm, 28 °C, for 24 h as the seed culture. Then, 300 mL seed culture was inoculated into a 10 L fermenter containing 6 L seed medium at 180 rpm, 28 °C, for 24 h. The fermentation conditions, pH (pH 4, pH 7 and pH 10) and culture time (18 h, 24 h, 30 h, 33 h, 36 h and 42 h), were screened. Lastly, the entire seed culture was transferred to a 300 L fermenter containing 200 L media (cotton seed meal, 2.5 g; soluble starch, 1 g; glucose, 1 g; yeast extract, 0.3 g; CaCO_3_, 0.5 g, and 100 mL distilled water, pH 4). After cultivation at 180 rpm, 28 °C, for 30 h, the bacterial culture broth was centrifuged at 3500 rpm to obtain mycelium. Then, the mycelium was broken using an ultrasonic treatment apparatus for 15 min and extracted three times with an equal volume of ethyl acetate. The organic extract was then concentrated under a vacuum to provide the crude extract (138.5 g).

### 4.3. Isolation and Purification

The EtOAc extract (120 g) was subjected to silica gel vacuum liquid chromatography using a step-gradient elution of petroleum ether (PE)–CH_2_Cl_2_ (1:0, 2:1, 0:1), CH_2_Cl_2_–MeOH (200:1, 100:1, 50:1, 30:1, 0:1), to yield eight fractions according to the TLC profiles (Frs.B1–B10). From Frs.B3, compounds **1** (1.1 mg, t_R_ = 41 min), **2** (2.5 mg, t_R_ = 32 min), **3** (2.0 mg, t_R_ = 45 min), **4** (0.9 mg, t_R_ = 48 min), **5** (2.1 mg, t_R_ = 43 min) and **9** (1.05 mg, t_R_ = 30 min) were further purified through a Sephadex LH-20 with CH_2_Cl_2_/MeOH (1:1, *v*/*v*) and semipreparative HPLC (80% MeOH/H_2_O, 2 mL/min, 230 nm). Frs.B4 was separated into four subfractions (Frs.B4-1–4-4) using ODS silica gel chromatography via elution with MeCN/H_2_O (5–100%). Frs.B4-2 was directly separated via semipreparative HPLC (80% MeOH/H_2_O, 2 mL/min, 230 nm) to yield **6** (1.01 mg, t_R_ = 34 min), **7** (0.93 mg, t_R_ = 37 min) and **8** (2.0 mg, t_R_ = 37 min).

Piericidin L (**1**): pale yellow oil; [*α*${]}_{D}^{25}$ + 3.1 (*c* 0.10, MeOH); UV (MeOH) λ_max_ (log ε) 200 (3.14), 226 (2.93) and 268 (2.37) nm; IR (film) *ν*_max_ 3377, 2920, 1670, 1585, 1411, 1190, 1124, 1045, 972, 721, 667 and 600 cm^−1^; CD (0.33 mg/mL, MeOH) λ_max_ (Δε) 201 (−2.69), 220 (+0.30), 235 (−1.23) and 267 (+0.27) nm; ^1^H and ^13^C NMR (see Table 1 and Table 2); and (+)-HR-ESIMS *m*/*z* 430.2583, [M + H]^+^ (calculated for C_25_H_36_NO_5_ 430.2588).

Piericidin N (**2**): brown oil; [*α*${]}_{D}^{25}$ + 2.5 (*c* 0.10, MeOH);UV (MeOH) λ_max_ (log ε) 211 (3.20), 223 (3.28), 227 (3.25) and 238 (3.30) nm; IR (film) *ν*_max_ 3273, 2926, 1606, 1541, 1506, 1456, 1373, 1232, 1192 and 827 cm^−1^; CD (0.33 mg/mL, MeOH) λ_max_ (Δε) 211 (+1.04), 239 (−1.37) and 254 (+0.21) nm; ^1^H and ^13^C NMR (see Table 1 and Table 2); and (+)-HR-ESIMS *m*/*z* 386.2696, [M + H]^+^ (calculated for C_24_H_36_NO_3_ 386.2690).

Piericidin Q (**3**): pale yellow oil; [*α*${]}_{D}^{25}$ + 3.7 (*c* 0.10, MeOH); UV (MeOH) λ_max_ (log ε) 219 (2.86) and 240 (3.01) nm; IR (film) *ν*_max_ 3369, 2926, 2854, 1699, 1587, 1558, 1471, 1141, 1126, 1035, 964, 800 and 725 cm^−1^; CD (0.33 mg/mL, MeOH) λ_max_ (Δε) 240 (+1.40) nm; ^1^H and ^13^C NMR (see Table 1 and Table 2); and (+)-HR-ESIMS *m*/*z* 438.2614, [M + Na]^+^ (calculated for C_25_H_37_NNaO_4_ 438.2615).

Piericidin O (**4**): pale yellow oil; [*α*${]}_{D}^{25}$ + 1.0 (*c* 0.10, MeOH); UV (MeOH) λ_max_ (log ε) 218 (2.85), 236 (2.95) and 263 (2.19) nm; IR (film) *ν*_max_ 3346, 2927, 1653, 1558, 1506, 1417 and 1035 cm^−1^; CD (0.33 mg/mL, MeOH) λ_max_ (Δε) 205 (−0.21), 210 (+0.07) and 237 (−1.28) nm; ^1^H and ^13^C NMR (see Table 1 and Table 2); and (+)-HR-ESIMS *m*/*z* 416.2799, [M + H]^+^ (calculated for C_25_H_38_NO_4_ 416.2795).

Piericidin P (**5**): pale yellow oil; [*α*${]}_{D}^{25}$ + 3.5 (*c* 0.10, MeOH); UV (MeOH) λ_max_ (log ε) 217 (3.31), 235 (3.30) and 260 (1.47) nm; IR (film) *ν*_max_ 3365, 2926, 2852, 1589, 1471, 1192, 1126, 1091, 1045 and 972 cm^−1^; CD (0.33 mg/mL, MeOH) λ_max_ (Δε) 210 (+1.43), 235 (−2.14) nm; ^1^H and ^13^C NMR (see Table 1 and Table 2); and (+)-HR-ESIMS *m*/*z* 416.2799, [M + H]^+^ (calculated for C_25_H_38_NO_4_ 416.2795).

Piericidin M (**6**): pale yellow oil; [*α*${]}_{D}^{25}$ + 2.4 (*c* 0.08, MeOH); UV (MeOH) λ_max_ (log ε) 200 (3.35), 219 (3.01), 232 (3.09) and 266 (2.39) nm; IR (film) *ν*_max_ 3381, 2920, 1471, 1456, 1412, 1190, 1124, 1026, 966, 825, 775 and 669 cm^−1^; CD (0.33 mg/mL, MeOH) λ_max_ (Δε) 206 (+1.01), 213 (+2.85), 232 (−3.17) and 253 (+0.43) nm; ^1^H and ^13^C NMR (see Table 1 and Table 2); and (+)-HR-ESIMS *m*/*z* 430.2955, [M + H]^+^ (calculated for C_26_H_40_NO_4_ 430.2952).

Piericidin R (**7**): pale yellow oil; [*α*${]}_{D}^{25}$ + 4.5 (*c* 0.09, MeOH); UV (MeOH) λ_max_ (log ε) 201 (3.12), 217 (2.89), 232 (2.98) and 268 (2.23) nm; IR (film) *ν*_max_ 3360, 2922, 1585, 1472, 1412, 1190, 1124, 1043, 964, 773, 660 and 600 cm^−1^; CD (0.33 mg/mL, MeOH) λ_max_ (Δε) 218 (−3.02), 235 (−7.33) and 260 (−0.49) nm; ^1^H and ^13^C NMR (see Table 1 and Table 2); and (+)-HR-ESIMS *m*/*z* 402.2640, [M + H]^+^ (calculated for C_24_H_36_NO_4_ 402.2639).

11-demethyl-glucopiericidin A (**8**): pale yellow oil; [*α*${]}_{D}^{25}$ + 1.6 (*c* 0.10, MeOH); UV (MeOH) λ_max_ (log ε) 200 (3.55), 217 (3.28), 237 (3.43), 260 (2.55) and 268 (2.60) nm; IR (film) *ν*_max_ 3390, 2929, 1683, 1653, 1558, 1506, 1456, 1417, 1126, 1076 and 1039 cm^−1^; CD (0.33 mg/mL, MeOH) λ_max_ (Δε) 200 (+0.02), 215 (+1.67) and 234 (−1.50) nm; ^1^H and ^13^C NMR (see Table 1 and Table 2); and (+)-HR-ESIMS *m*/*z* 564.3162, [M + H]^+^ (calculated for C_30_H_46_NO_9_ 564.3167).

### 4.4. Mono-MTPA Esters of Piericidin Q (3)

Piericidin Q (**3**, 0.6 mg) was dissolved in freshly distilled dry pyridine (2 mL) with dry crystals of dimethylaminopyridine (DMAP 0.3 mg). The treatment with (*R*)-MTPA-Cl at 28 °C yielded the *S*-MTPA ester after 16 h. The reaction mixture was purified by semipreparative HPLC (95% CH_3_CN in H_2_O) to produce the *S*-MTPA ester (**3A**) after 30 min. The *R*-MTPA ester (**3B**) was prepared with *S*-MTPA-Cl in the same manner. The ∆δ *_S-R_* values for the mono-*S*- and *R*-MTPA esters of piericidin Q were recorded in ppm in CD_3_OD (Table S7).

### 4.5. Acid Hydrolysis of 11-Demethyl-Glucopiericidin A (8)

The 11-Demethyl-glucopiericidin A (**8**, 1 mg) was refluxed with 2.0 M HCl (2 mL) for 6 h at 80 °C. The reaction mixture was then evaporated to dryness and diluted with H_2_O (2 mL). After extraction with EtOAc (3 × 2 mL), the aqueous layer was concentrated and heated with L-Cysteine Methyl Ester Hydrochloride (3 mg) in pyridine (1 mL) at 60 °C for 1 h. Then, *o*-Tolyl isothiocyanate (0.4 mL) was added to the reaction mixture, which was subsequently stirred at 60 °C for 1 h. The sugar (d-glucose) standard was derivatized using L-Cysteine Methyl Ester Hydrochloride and *o*-Tolyl isothiocyanate in the same manner. The reaction mixtures were analyzed using reversed-phase HPLC under the following conditions: a YMC-Pack ODS-A column (250 × 4.6 mm, 5 μm), a UV detector, the CH_3_CN/H_2_O mobile phase (20/80, *v*/*v*, 0.08%TFA), a detection wavelength of 250 nm, and a 0.8 mL/min flow rate. Comparing the retention times with those of the derivative of an authentic sample of d-glucose (retention time: 26.97 min, Figure S81) confirmed the d-configuration of the glucose.

### 4.6. ECD Calculation

The relative configurations of **3**, **4** and **7** were subjected to random conformational searches using the Spartan’14 software with the MMFF method [21]. The conformers with a Boltzmann population of over 5% (relative energy within 6 kcal/mol) were chosen for the ECD calculations using the Gaussian 09 software [22], and the stable conformers were initially optimized at the B3LYP/6-31+G(d,p) level in MeOH using the CPCM model. The overall theoretical calculation of ECD was achieved in MeOH using time-dependent density functional theory at the B3LYP/6-31+G(d,p) level for the stable conformers of **3**, **4**, and **7**. The rotatory strengths were calculated for a total of 30 excited states. The ECD spectra of the different conformers were generated using SpecDis 1.6 (University of Würzburg) and Prism 5.0 (GraphPad Software Inc., San Diego, CA, USA) software, with a half-bandwidth of 0.3−0.4 eV, according to the Boltzmann-calculated contribution of each conformer after UV correction.

### 4.7. Cell Culture and Cytotoxic Bioassay

HL-60, K-562, MOLT-4, ACHN, OS-RC-2 and 786-O cells were purchased from Shanghai Cell Bank, Chinese Academy of Sciences. The ACHN cells were grown and maintained in an MEM medium with 10% FBS, while the other cells were grown in a RPMI1640 medium with 10% FBS. The cell viability was determined using a CCK-8 (Dojindo) assay [23]. The cells were seeded at a density of 400 to 800 cells/well in 384-well plates, and were then treated with various concentrations (50, 10, 2, 0.4 and 0.08 μM) of compounds or a solvent control. After 72 h of incubation, the CCK-8 reagent was added, and the absorbance of the triplicate tests was measured at 450 nm using an Envision 2104 multi-label reader (Perkin Elmer). The dose–response curves were plotted in order to determine the IC_50_ using Prism 5.0 (GraphPad Software Inc.).

### 4.8. Cell Cycle and Apoptosis Assay

The cell cycle arrests by **1** and **6** in OS-RC-2 cells, by **2** in HL-60 cells, and by **8** in ACHN cells were analyzed via PI DNA staining using flow cytometry [23,24]. In summary, the cells were treated with compounds under suitable concentrations (5 μM for **1** and **6**; 0.1 and 0.4 μM for **2**; 2 μM for **8**) for 24, 48 and 72 h. After the treatment, the cells were harvested, prepared and fixed overnight. The fixed cells were then harvested, washed, re-suspended, and finally stained with PI (Sigma-Aldrich). The cell-cycle distribution was studied using an Accuri C6 (BD) flow cytometer. The cell apoptosis was analyzed using a FITC annexin V apoptosis detection kit (BD), according to the manufacturer’s protocol [24]. The cells were treated with the compounds under suitable concentrations (5 μM for **1** and **6**; 0.1 and 0.4 μM for **2**; 2 μM for **8**) for 24, 48 and 72 h, stained with annexin V-FITC and PI solution, examined, and analyzed quantitatively using an Accuri C6 (BD) flow cytometer.

## 5. Conclusions

In this study, eight new minor piericidin derivatives were obtained from the sediment-derived actinomycete strain *Streptomyces psammoticus* SCSIO NS126, which was fermented at a 300-L scale under optimized fermentation conditions. The new structures including absolute configurations were determined by spectroscopic methods coupled with experimental and calculated ECD. The plausible biosynthetic pathways for these unusual post-modified piericidins were also proposed. Most of the derivatives showed obvious cytotoxic activities against several cancer cells. It is suggested that some unusual post-modified piericidins, such as carbonyl-containing piericidin, possess selective anti-tumor potential. This study increases our knowledge on the structural diversity and cytotoxicity of actinomycete-derived piericidin compounds, and confirms that piericidins deserve further investigations as promising anti-tumor agents.

## Figures and Tables

**Table 1:** ^1^H NMR data of **1**–**8** (700 MHz, *δ* in ppm).

Pos.	1 ^a^	2 ^a^	3 ^a^	4 ^b^	5 ^b^	6 ^c^	7 ^a^	8 ^a^
1	3.39 (d, 6.9)	3.36 (d, 6.9)	3.42 (d, 6.9)	3.37 (d, 6.9)	3.37 (d, 6.9)	3.25 (d, 6.9)	3.35 (d, 6.9)	3.48 (d, 6.9)
2	5.28 (t, 6.9)	5.18 (t, 6.9)	5.27 (t, 6.9)	5.41 (t, 6.9)	5.40 (d, 6.9)	5.30 (t, 6.9)	5.31 (t, 6.9)	5.26 (t, 6.9)
4	2.76 (d, 7.1)	2.79 (d, 6.9)	2.77 (d, 7.0)	2.78 (d, 6.9)	2.78 (d, 6.7)	2.72 (d, 6.8)	2.76 (d, 6.9)	2.80 (d, 7.1)
5	5.45 (m)	5.50 (m)	5.53(m)	5.57 (m)	5.52 (m)	5.48 (m)	5.53 (m)	5.55 (m)
6	6.02 (d,15.8)	6.08 (d, 5.5)	6.02 (d, 6.2)	6.05 (d, 15.8)	6.07 (d, 6.0)	5.86 (d, 5.7)	6.03(d,15.5)	6.08 (d, 6.1)
8	5.26 (d, 10.4)	5.30 (d, 9.2)	5.22 (d, 9.2)	5.20 (d, 9.1)	5.26 (d, 9.4)	5.19 (d, 9.7)	5.30 (t, 6.7)	5.30 (d, 9.5)
9	2.75 (m)	2.64 (m)	3.42 (m)	3.18 (m)	2.63 (m)	2.50 (m)	2.35 (m)	2.83 (m)
10	4.52 (d, 4.0)	3.69 (d, 8.2)	5.26 (d, 9.0)	5.53 (m)	3.31 (m)	3.59 (d, 7.1)	3.96 (t, 7.0)	4.00 (d, 8.0)
11				5.27(m)	5.26 (m)			5.52 (m)
12		5.44 (q, 6.8)	4.11 (q, 6.9)	3.65 (m)	5.60 (m)	5.32 (q, 6.4)	5.44 (q, 5.9)	5.65 (m)
13	2.30 (s)	1.62 (d, 6.8)	1.20 (d, 6.9)	1.21 (dd, 2.1, 6.3)	1.73 (d, 6.7)	1.54 (d, 6.6)	1.60 (d, 5.4)	1.71 (d, 6.4)
14	6.21 (s); 6.01 (s)	1.60 (s)	1.65 (d, 1.3)	3.23 (s)	3.22 (s)	1.50 (s)	1.60 (s)	
15	1.03 (d, 6.9)	0.81 (d, 6.9)	1.01 (d, 6.8)	1.08 (t, 6.3)	0.93 (d, 6.9)	0.77 (d, 6.8)		1.02 (d, 6.9)
16	1.57 (s)	1.74 (s)	1.74 (s)	1.74 (s)	1.72 (s)	2.17(m); 2.12 (m)	1.71 (s)	1.75 (s)
17	1.74 (s)	1.74 (s)	1.74 (s)	1.75 (s)	1.74 (s)	1.70 (s)	1.73 (s)	1.78 (s)
18						0.93 (t, 7.4)		
3′		5.88 (s)						
7′	3.96 (s)	3.86 (s)	4.00 (s)	3.95 (s)	3.95 (s)	3.77 (s)	3.90 (s)	4.09 (s)
8′	3.76 (s)		3.77 (s)	3.86 (s)	3.86 (s)	3.61 (s)	3.74 (s)	3.81 (s)
9′	2.06 (s)	1.96 (s)	2.07 (s)	2.09 (s)	2.09 (s)	1.95 (s)	2.04 (s)	2.11 (s)
1″								4.33 (d, 7.8)
2″								3.20 (m)
3″								3.36(m)
4″								3.66(m)
5″								3.20 (m)
6″								3.65 (m); 3.78 (m)

^a^ Data measured in CD_3_OD. ^b^ Data measured in CDCl_3_. ^c^ Data measured in DMSO-*d*_6_.

**Table 2:** ^13^C NMR data of **1**–**8** (175 MHz, *δ* in ppm).

Pos.	1 ^a^	2 ^a^	3 ^a^	4 ^b^	5 ^b^	6 ^c^	7 ^a^	8 ^a^
1	34.8, CH_2_	32.3, CH_2_	34.1, CH_2_	34.6, CH_2_	34.6, CH_2_	34.2, CH_2_	34.9, CH_2_	33.4, CH_2_
2	122.9, CH	121.0, CH	122.2, CH	122.2, CH	122.1, CH	122.2, CH	123.4, CH	121.5, CH
3	136.7, C	138.4, C	137.1, C	135.1, C	135.2, C	133.9, C	135.9, C	137.9, CH
4	43.9, CH_2_	43.9, CH_2_	43.9, CH_2_	43.3, CH_2_	43.3, CH_2_	42.4, CH_2_	44.0, CH_2_	43.9, CH_2_
5	126.4, CH	125.7, CH	126.3, CH	126.2, CH	125.6, CH	124.2, CH	126.5, CH	126.2, CH
6	137.6, CH	138.1, CH	137.6, CH	136.0, CH	136.4, CH	134.5, CH	137.4, CH	137.8, CH
7	135.2, C	134.8, C	132.7, C	133.0, C	133.5, C	138.1, C	135.9, C	134.6, C
8	132.9, CH	135.8, CH	136.6, CH	134.4, CH	133.6, CH	134.6, CH	127.8, CH	134.4, CH
9	38.4, CH	37.6, CH	32.6, CH	35.2, CH	37.6, CH	36.1, CH	34.9, CH_2_	38.1, CH
10	73.7, CH	83.7, CH	130.3, CH	137.1, CH	86.8, CH	80.4, CH	78.5, CH	86.1, CH
11	152.0, C	136.6, C	138.0, CH	129.8, CH	129.9, CH	137.8, C	138.7, C	131.5, CH
12	201.8, C	122.8, CH	73.9, CH	78.2, CH	129.9, CH	119.4, CH	121.5, CH	129.4, CH
13	26.5, CH_3_	13.1, CH_3_	22.0, CH_3_	21.6, CH_3_	17.9, CH_3_	12.8, CH_3_	13.1, CH_3_	18.0, CH_3_
14	126.8, CH_2_	11.1, CH_3_	11.8, CH_3_	55.8, CH_3_	56.3, CH_3_	11.4, CH_3_	11.1, CH_3_	-
15	18.5, CH_3_	18.1, CH_3_	21.8, CH_3_	21.0, CH_3_	16.8, CH_3_	18.3, CH_3_	-	16.4, CH_3_
16	13.0, CH_3_	13.1, CH_3_	12.9, CH_3_	12.8, CH_3_	13.0, CH_3_	19.7, CH_2·_	12.9, CH_3_	13.0, CH_3_
17	16.7, CH_3_	16.7, CH_3_	16.7, CH_3_	16.8, CH_3_	16.8, CH_3_	16.5, CH_3_	16.6, CH_3_	16.7, CH_3_
18	-	-	-	-	-	13.8, CH_3_	-	-
2′	155.9, C	161.8, C	155.8, C	153.7, C	153.6, C	154.3, C	156.1, C	155.7, C
3′	130.6, C	92.8, CH	131.2, C	127.9, C	127.9, C	128.5, C	130.1, C	131.5, C
4′	158.5, C	179.1, C	159.8, C	154.1, C	154.1, C	157.1, C	157.2, C	161.6, C
5′	115.2, C	117.6, C	115.9, C	112.1, C	112.1, C	112.9, C	114.8, C	116.8, C
6′	151.2, C	149.8, C	150.5, C	151.0, C	151.1, C	148.9, C	151.7, C	149.9, C
7′	54.4, CH_3_	56.1, CH_3_	55.7, CH_3_	53.2, CH_3_	53.2, CH_3_	52.3, CH_3_	53.7, CH_3_	57.3, CH_3_
8′	61.0, CH_3_	-	61.2, CH_3_	60.8, CH_3_	60.8, CH_3_	59.7, CH_3_	60.8, CH_3_	61.4, CH_3_
9′	10.8, CH_3_	10.3, CH_3_	10.7, CH_3_	10.6, CH_3_	10.6, CH_3_	10.6, CH_3_	10.8, CH_3_	10.7, CH_3_
1″	-	-	-	-	-	-	-	103.3, CH
2″	-	-	-	-	-	-	-	75.5, CH
3″	-	-	-	-	-	-	-	78.3, CH
4″	-	-	-	-	-	-	-	71.6, CH
5″	-	-	-	-	-	-	-	77.9, CH
6″	-	-	-	-	-	-	-	62.7, CH_2_

^a^ Data measured in CD_3_OD. ^b^ Data measured in CDCl_3_. ^c^ Data measured in DMSO-*d*_6_.

**Table 3:** Cytotoxic activities of **1**–**8** against six cancer cells (IC_50_ μM) ^a^.

No.	ACHN	786-O	OS-RC-2	HL-60	K562	MOLT-4
**1**	>50	>50	2.2 ± 0.21	12 ± 1.02	>50	>50
**2**	>50	>50	42.9 ± 5.3	0.08 ± 0.01	>50	/
**3**	>50	>50	>50	0.08 ± 0.01	>50	/
**4**	>50	>50	>50	0.08 ± 0.01	>50	/
**5**	>50	>50	>50	0.1 ± 0.01	>50	/
**6**	>50	>50	4.5 ± 0.37	9.8 ± 0.52	>50	>50
**7**	>50	>50	15 ± 1.3	>50	>50	>50
**8**	2.3 ± 0.32	12.0 ± 1.2	28.7 ± 3.2	1.3 ± 0.14	5.5 ± 0.69	/
PA ^b^	0.40 ± 0.02	30 ± 3.2	5.2 ± 0.67	8.5 ± 0.86	2.4 ± 0.28	25 ± 2.6

^a^ Mean ± SD (n = 5). IC_50_ was calculated using concentrations of 50, 10, 2, 0.4, 0.08 μM. ^b^ PA was used as a positive control.

## Supplementary Materials

The following are available online at https://www.mdpi.com/article/10.3390/md19080428/s1. Figures S1–S3: The optimized conformers and equilibrium populations of **3a**, **4a**/**4b**, and **7a**/**7b**; Figures S4–S80: 1D and 2D NMR for compounds **1**–**9**; Figure S81: The HPLC results for D-glucose and 8 via acidic hydrolysis; Figures S82–S84: The HPLC analysis of crude extracts under different fermentation conditions; Tables S1–S6: Energies of **3a**, **4a**/**4b**, and **7a**/**7b** in ECD calculations; Table S7: ^1^H NMR Data of **3A**/**3B**.
