# Supplementary material for: Cytotoxic Minor Piericidin Derivatives from the Actinomycete Strain Streptomyces psammoticus SCSIO NS126
Source: Mar Drugs. 2021 Jul 28;19(8):428. doi: 10.3390/md19080428 (PMC8398042; doi:10.3390/md19080428)
Supplement: Supplementary file 1 [file marinedrugs-19-00428-s001.zip › marinedrugs-1300553-supplementary.pdf]

Supporting information

Cytotoxic minor piericidin derivatives from the actinomycete strain *Streptomyces psammoticus* SCSIO NS126

Kunlong Li [a,b,f], Ziqi Su [c], Yongli Gao [b,d], Xiuping Lin [a], Xiaoyan Pang [a], Bin Yang [a,b], Huaming Tao [c], Xiaowei Luo [e,*], Yonghong Liu [a,b,e,f,*], Xuefeng Zhou [a,b,*]

[a] CAS Key Laboratory of Tropical Marine Bio-resources and Ecology, Guangdong Key Laboratory of Marine Materia Medica, South China Sea Institute of Oceanology, Chinese Academy of Sciences, Guangzhou, 510301, China; likunlong16@mails.ucas.ac.cn (K.L.); xiupinglin@hotmail.com (X.L.); xypang@scsio.ac.cn (X.P.); yangbin@scsio.ac.cn (B.Y.)

[b] Southern Marine Science and Engineering Guangdong Laboratory (Guangzhou), Guangzhou, 511458, China; yongligao@scsio.ac.cn (Y.G.)

[c] School of Traditional Chinese Medicine, Southern Medical University, Guangzhou 510515, China; ziqisl @163.com (Z.S.); taohm@smu.edu.cn (H.T.)

[d] Institutional Center for Shared Technologies and Facilities, South China Sea Institute of Oceanology, Chinese Academy of Sciences, Guangzhou, 510301, China

[e] Institute of Marine Drugs, Guangxi University of Chinese Medicine, Nanning 530200, China

[f] College of Earth and Planetary Sciences, University of Chinese Academy of Sciences, Beijing, 100049, China

*Correspondence: xfzhou@scsio.ac.cn (X.Z.); yonghongliu@scsio.ac.cn (Y.L.); luoxiaowei1991@126.com (X.L.); Tel: +86-020-89023174 (X.Z.)

List of Supporting Information

Table S1. Energies of **3a** at MMFF94 force field

Table S2. Energies of **3a** at B3LYP/6–31+g(d) level in methanol.

Figure S1. The optimized conformers and equilibrium populations of **3a**

Table S3. Energies of **4a/4b** at MMFF94 force field

Table S4. Energies of **4a/4b** at B3LYP/6–31+g(d) level in methanol.

Figure S2. The optimized conformers and equilibrium populations of **4a/4b**

Table S5. Energies of **7a/7b** at MMFF94 force field

Table S6. Energies of **7a/7b** at B3LYP/6–31+g(d) level in methanol.

Figure S3. The optimized conformers and equilibrium populations of **7a**/**7b**

Table S7. ^{1}H NMR Data of **3A** and **3B** (CD_3OD, 700MHz)

Figure S4. ^{1}H NMR spectrum of piericidin L (**1**) (CD_3OD, 700MHz)

Figure S5. ^{13}C NMR spectrum of piericidin L (**1**) (CD_3OD, 175MHz)

Figure S6. ^{1}H-^{1}H COSY spectrum of piericidin L (**1**) (CD_3OD)

Figure S7. HSQC spectrum of piericidin L (**1**) (CD_3OD)

Figure S8. HMBC spectrum of piericidin L (**1**) (CD_3OD)

Figure S9. NOESY spectrum of piericidin L (**1**) (CD_3OD)

Figure S10. UV spectrum of piericidin L (**1**)

Figure S11. IR spectrum of piericidin L (**1**)

Figure S12. HRESIMS spectrum of piericidin (**1**)

Figure S13. ^{1}H NMR spectrum of piericidin M (**2**) (CD_3OD, 700MHz)

Figure S14. ^{13}C NMR spectrum of piericidin M (**2**) (CD_3OD, 175MHz)

Figure S15. ^{1}H-^{1}H COSY spectrum of piericidin M (**2**) (CD_3OD)

Figure S16. HSQC spectrum of piericidin M (**2**) (CD_3OD)

Figure S17. HMBC spectrum of piericidin M (**2**) (CD_3OD)

Figure S18. NOESY spectrum of piericidin M (**2**) (CD_3OD)

Figure S19. UV spectrum of piericidin M (**2**)

Figure S20. IR spectrum of piericidin M (**2**)

Figure S21. HRESIMS spectrum of piericidin M (**2**)

Figure S22. ^{1}H NMR spectrum of piericidin N (**3**) (CD_3OD, 700MHz)

Figure S23. ^{13}C NMR spectrum of piericidin N (**3**) (CD_3OD, 175MHz)

Figure S24. ^{1}H-^{1}H COSY spectrum of piericidin N (**3**) (CD_3OD)

Figure S25. HSQC spectrum of piericidin N (**3**) (CD_3OD)

Figure S26. HMBC spectrum of piericidin N (**3**) (CD_3OD)

Figure S27. NOESY spectrum of piericidin N (**3**) (CD_3OD)

Figure S28. UV spectrum of piericidin N (**3**)

Figure S29. IR spectrum of piericidin N (**3**)

Figure S30. HRESIMS spectrum of piericidin N (**3**)

Figure S31 .^{1}H NMR spectrum of **3A** (CD_3OD, 700MHz)

Figure S32. ^{1}H-^{1}H COSY spectrum of **3A** (CD_3OD)

Figure S33. ^{1}H NMR spectrum of **3B** (CD_3OD, 700MHz)

Figure S34. ^{1}H-^{1}HCOSY spectrum of **3B** (CD_3OD)

Figure S35. ^{1}H NMR spectrum of piericidin O (**4**) (CD_3OD, 700MHz)

Figure S36. ^{13}C NMR spectrum of piericidin O (**4**) (CD_3OD, 175MHz)

Figure S37. ^{1}H-^{1}H COSY spectrum of piericidin O (**4**) (CD_3OD)

Figure S38. HSQC spectrum of piericidin O (**4**) (CD_3OD)

Figure S39. HMBC spectrum of piericidin O (**4**) (CD_3OD)

Figure S40. NOESY spectrum of piericidin O (**4**) (CD_3OD)

Figure S41. UV spectrum of piericidin O (**4**)

Figure S42. IR spectrum of piericidin O (**4**)

Figure S43. HRESIMS spectrum of piericidin O (**4**)

Figure S44. ^{1}H NMR spectrum of piericidin P (**5**) (CD_3OD, 700MHz)

Figure S45. ^{13}C NMR spectrum of piericidin P (**5**) (CD_3OD, 175MHz)

Figure S46. ^{1}H-^{1}H COSY spectrum of piericidin P (**5**) (CD_3OD)

Figure S47. HSQC spectrum of piericidin P (**5**) (CD_3OD)

Figure S48. HMBC spectrum of piericidin P (**5**) (CD_3OD)

Figure S49. NOESY spectrum of piericidin P (**5**) (CD_3OD)

Figure S50. UV spectrum of piericidin P (**5**)

Figure S51. IR spectrum of piericidin P (**5**)

Figure S52. HRESIMS spectrum of piericidin (**5**)

Figure S53. ^{1}H NMR spectrum of piericidin Q (**6**) (DMSO-d_6, 700MHz)

Figure S54. ^{13}C NMR spectrum of piericidin Q (**6**) (DMSO-d_6, 175MHz)

Figure S55. ^{1}H-^{1}H COSY spectrum of piericidin Q (**6**) (DMSO-d_6)

Figure S56. HSQC spectrum of piericidin Q (**6**) (DMSO-d_6)

Figure S57. HMBC spectrum of piericidin Q (**6**) (DMSO-d_6)

Figure S58. NOESY spectrum of piericidin Q (**6**) (DMSO-d_6)

Figure S59. UV spectrum of piericidin Q (**6**)

Figure S60. IR spectrum of piericidin Q (**6**)

Figure S61. HRESIMS spectrum of piericidin Q (**6**)

Figure S62. ^{1}H NMR spectrum of piericidin R (**7**) (CD_3OD, 700MHz)

Figure S63. ^{13}C NMR spectrum of piericidin R (**7**) (CD_3OD, 175MHz)

Figure S64. ^1H-^1H COSY spectrum of piericidin R (**7**) (CD_3OD)
Figure S65. HSQC spectrum of piericidin R (**7**) (CD_3OD)
Figure S66. HMBC spectrum of piericidin R (**7**) (CD_3OD)
Figure S67. NOESY spectrum of piericidin R (**7**) (CD_3OD)
Figure S68. UV spectrum of piericidin R (**7**)
Figure S69. IR spectrum of piericidin R (**7**)
Figure S70. HRESIMS spectrum of piericidin R (**7**)
Figure S71. ^1H NMR spectrum of compound **8** (CD_3OD, 700MHz)
Figure S72. ^{13}C NMR spectrum of compound **8** (CD_3OD, 175MHz)
Figure S73. ^1H-^1H COSY spectrum of compound **8** (CD_3OD)
Figure S74. HSQC spectrum of compound **8** (CD_3OD)
Figure S75. HMBC spectrum of compound **8** (CD_3OD)
Figure S76. NOESY spectrum of compound **8** (CD_3OD)
Figure S77. UV spectrum of compound **8**
Figure S78. IR spectrum of compound **8**
Figure S79. HRESIMS spectrum of compound **8**
Figure S80. ^1H NMR spectrum of compound **9** (CD_3OD, 700MHz)
Figure S81. The HPLC results for D-glucose and **8** by acidic hydrolysis
Figure S82. The HPLC analysis of crude extract in different external pH
Figure S83. The content analysis of PA and GPA in different fermentation time
Figure S84. The HPLC analysis of crude extract in different fermentation time

Table S1. Energies of **3a** at MMFF94 force field

Configuration	Conformer	Energy (kcal/mol)	Population (%)
9*R*-**3a**	1	54.06	58.6
9*R*-**3a**	2	56.12	25.5
9*R*-**3a**	3	58.00	11.9
9*R*-**3a**	4	63.10	1.5
9*R*-**3a**	5	64.36	0.9
9*R*-**3a**	6	66.17	0.4

Table S2. Energies of **3a** at B3LYP/6–31+g(d) level in methanol.

Configuration	Conformer	E (Hartree)	E (kcal/mol)	Population (%)
9*R*-**3a**	1	– 506.5650469	– 317874.632580219	63.6
9*R*-**3a**	2	– 506.5626421	– 317873.123544171	4.97
9*R*-**3a**	3	– 506.5637643	– 317873.827735893	16.33
9*R*-**3a**	4	– 506.5633788	– 317873.585830788	10.85
9*R*-**3a**	5	– 506.5624711	– 317873.016239961	4.15
9*R*-**3a**	6	– 506.5590446	– 317870.866076946	0.11

9*R*-**3a**

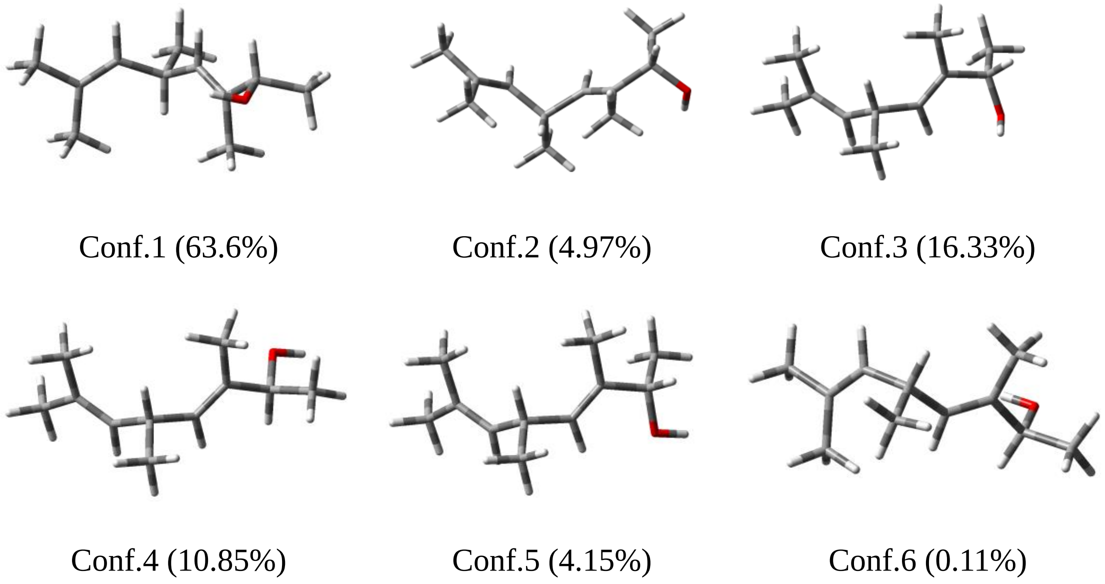

Figure S1. The optimized conformers and equilibrium populations of **3a**

Table S3. Energies of **4a/4b** at MMFF94 force field

Configuration	Conformer	Energy (kcal/mol)	Population (%)
9*S*, 12*S*-**4a**	1	81.27	30.2
9*S*, 12*S*-**4a**	2	81.96	22.9
9*S*, 12*S*-**4a**	3	84.37	8.7
9*S*, 12*S*-**4a**	4	85.63	5.2
9*S*, 12*S*-**4a**	5	85.63	5.2
9*S*, 12*S*-**4a**	6	85.93	4.6
9*S*, 12*S*-**4a**	7	86.70	3.4
9*S*, 12*S*-**4a**	8	86.70	3.4
9*S*, 12*S*-**4a**	9	87.74	2.2
9*S*, 12*S*-**4a**	10	87.86	2.1
9*S*, 12*R*-**4b**	1	81.59	26.8
9*S*, 12*R*-**4b**	2	81.73	25.4
9*S*, 12*R*-**4b**	3	84.69	7.7
9*S*, 12*R*-**4b**	4	84.74	7.5
9*S*, 12*R*-**4b**	5	85.22	6.2
9*S*, 12*R*-**4b**	6	85.97	4.6
9*S*, 12*R*-**4b**	7	86.59	3.6
9*S*, 12*R*-**4b**	8	86.70	3.4
9*S*, 12*R*-**4b**	9	87.56	2.4
9*S*, 12*R*-**4b**	10	88.76	1.5

Table S4. Energies of **4a/4b** at B3LYP/6–31+g(d) level in methanol.

Configuration	Conformer	E (Hartree)	E (kcal/mol)	Population
9*S*, 12*S*-**4a**	1	–506.5485087	–317864.254694337	47.85
9*S*, 12*S*-**4a**	2	– 506.5475945	–317863.681024695	18.15
9*S*, 12*S*-**4a**	3	–506.5475112	–317863.628753112	16.62
9*S*, 12*S*-**4a**	4	–506.5460802	–317862.730786302	3.65
9*S*, 12*S*-**4a**	5	–506.5455304	–317862.385781304	2.04
9*S*, 12*S*-**4a**	6	–506.5451027	–317862.117395277	1.29
9*S*, 12*S*-**4a**	7	–506.5464565	–317862.966918315	5.43
9*S*, 12*S*-**4a**	8	–506.5451652	–317862.156614652	1.38
9*S*, 12*S*-**4a**	9	–506.5455823	–317862.418349073	2.15

9*S*, 12*S*-**4a**	10	–506.5451999	–317862.178389249	1.43
9*S*, 12*R*-**4b**	1	–506.5484928	–317864.244716928	48.28
9*S*, 12*R*-**4b**	2	–506.5475185	–317863.633333935	17.19
9*S*, 12*R*-**4b**	3	–506.5475294	–317863.640173794	17.39
9*S*, 12*R*-**4b**	4	–506.5461035	–317862.745407285	3.84
9*S*, 12*R*-**4b**	5	–506.5464623	–317862.970557873	5.61
9*S*, 12*R*-**4b**	6	–506.5454718	–317862.349009218	1.96
9*S*, 12*R*-**4b**	7	–506.545141	–317862.14142891	1.38
9*S*, 12*R*-**4b**	8	–506.5450981	–317862.114508731	1.32
9*S*, 12*R*-**4b**	9	–506.5456025	–317862.431024775	2.25
9*S*, 12*R*-**4b**	10	–506.5445943	–317861.798369193	0.77

9*S*, 12*S*-**4a**

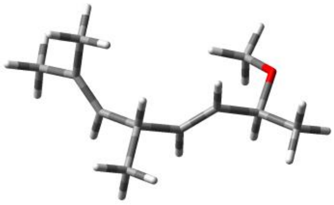

Conf.1 (47.85%)

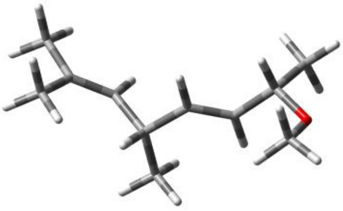

Conf.2 (18.15%)

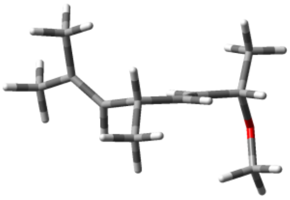

Conf.3 (16.62%)

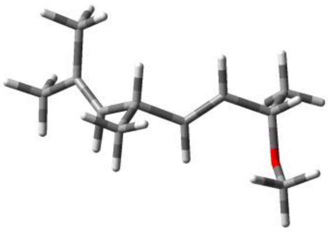

Conf.4 (3.65%)

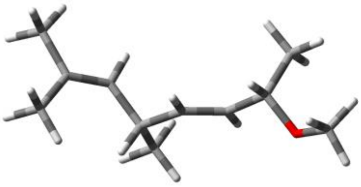

Conf.5 (2.04%)

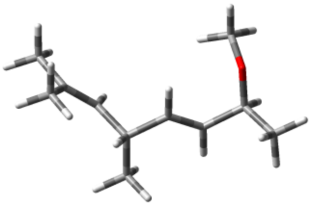

Conf.6 (1.29%)

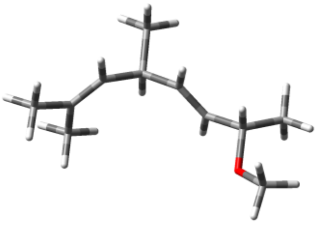

Conf.7 (5.43%)

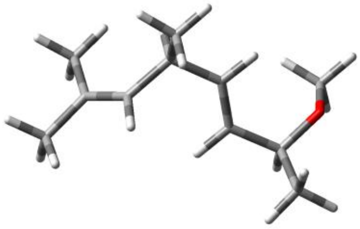

Conf.8 (1.38%)

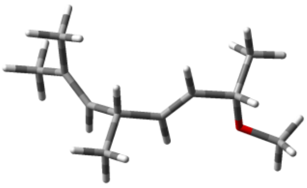

Conf.9 (2.15%)

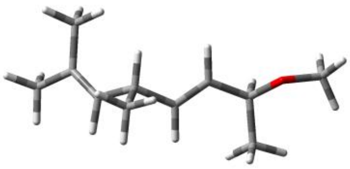

Conf.10 (1.43%)

9*S*, 12*R*-**4b**

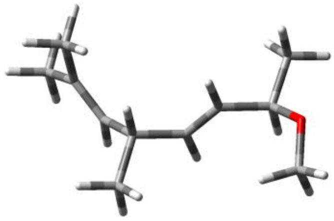

Conf.1 (48.28%)

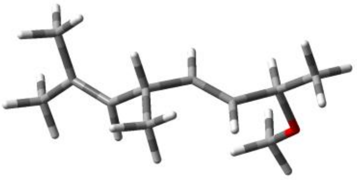

Conf.2 (17.19%)

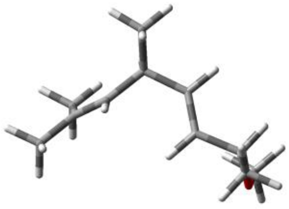

Conf.3 (17.39%)

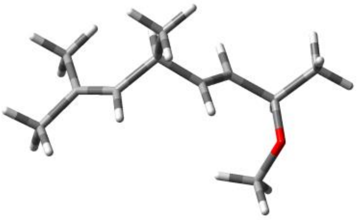

Conf.4 (3.84%)

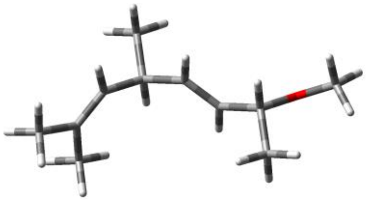

Conf.5 (5.61%)

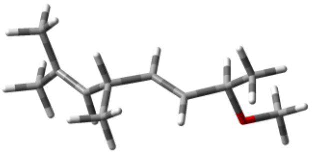

Conf.6 (1.96%)

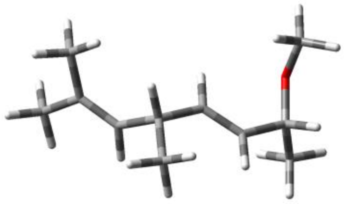

Conf.7 (1.38%)

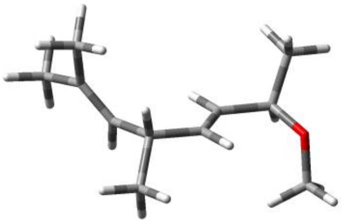

Conf.8 (1.32%)

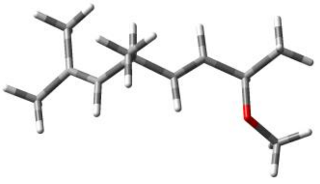

Conf.9 (2.25%)

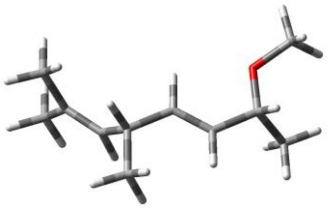

Conf.10 (0.77%)

Figure S2. The optimized conformers and equilibrium populations of **4a/4b**

Table S5. Energies of **7a/7b** at MMFF94 force field

Configuration	Conformer	Energy (kcal/mol)	Population (%)
10*R*-**7a**	1	111.43	33.8
10*R*-**7a**	2	113.24	16.3
10*R*-**7a**	3	114.86	8.5

10*R*-**7a**	4	115.22	7.3
10*R*-**7a**	5	115.33	7.0
10*R*-**7a**	6	115.44	6.7
10*R*-**7a**	7	116.71	4.0
10*R*-**7a**	8	117.00	3.6
10*R*-**7a**	9	117.46	3.0
10*R*-**7a**	10	118.33	2.1
10*S*-**7b**	1	111.43	30.7
10*S*-**7b**	2	113.24	14.8
10*S*-**7b**	3	114.41	9.3
10*S*-**7b**	4	114.86	7.7
10*S*-**7b**	5	115.22	6.7
10*S*-**7b**	6	115.33	6.4
10*S*-**7b**	7	115.44	6.1
10*S*-**7b**	8	116.71	3.7
10*S*-**7b**	9	117.00	3.3
10*S*-**7b**	10	117.46	2.7

Table S6. Energies of **7a/7b** at B3LYP/6–31+g(d) level in methanol.

Configuration	Conformer	E (Hartree)	E (kcal/mol)	Population
10*R*-**7a**	1	– 467.2404965	–293198.083958715	13.79
10*R*-**7a**	2	– 467.2412531	–293198.558732781	30.76
10*R*-**7a**	3	– 467.2395649	–293197.499370399	5.14
10*R*-**7a**	4	–467.2391298	–293197.226340798	3.24
10*R*-**7a**	5	–467.2392257	–293197.286519007	3.59
10*R*-**7a**	6	–467.2406097	–293198.154992847	15.55
10*R*-**7a**	7	–467.2382961	–293196.703185711	1.34
10*R*-**7a**	8	–467.2403141	–293197.969500891	11.37
10*R*-**7a**	9	–467.2405155	–293198.095881405	14.07
10*R*-**7a**	10	–467.2381497	–293196.611318247	1.15
10*S*-**7b**	1	–467.2404965	–293198.083958715	12.04
10*S*-**7b**	2	–467.2412531	–293198.558732781	26.85
10*S*-**7b**	3	–467.2406188	–293198.160703188	13.71
10*S*-**7b**	4	–467.2395649	–293197.499370399	4.48

10*S*-**7b**	5	–467.2391298	–293197.226340798	2.83
10*S*-**7b**	6	–467.2392257	–293197.286519007	3.13
10*S*-**7b**	7	–467.2406097	–293198.154992847	13.58
10*S*-**7b**	8	–467.238296	–293196.70312296	1.17
10*S*-**7b**	9	–467.2403141	–293197.969500891	9.92
10*S*-**7b**	10	–467.2405155	–293198.095881405	12.29

10*R*-**7a**

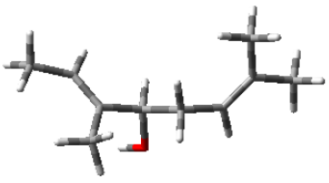

Conf.1 (13.79%)

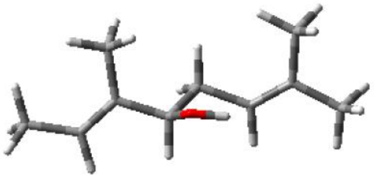

Conf.2 (30.76%)

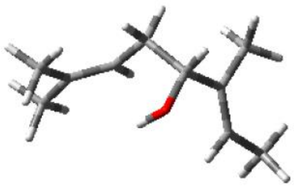

Conf.3 (5.14%)

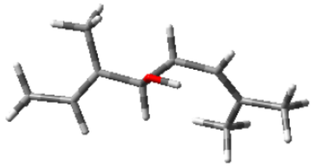

Conf.4 (3.24%)

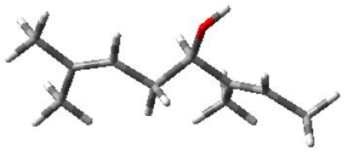

Conf.5 (3.59%)

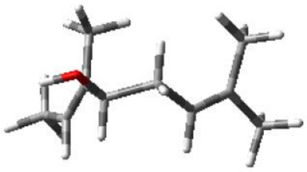

Conf.6 (15.55%)

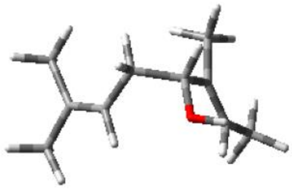

Conf.7 (1.34%)

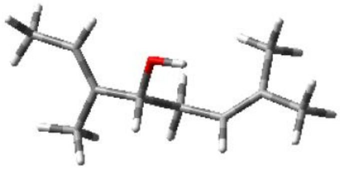

Conf.8 (11.37%)

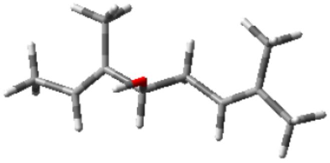

Conf.9 (14.07%)

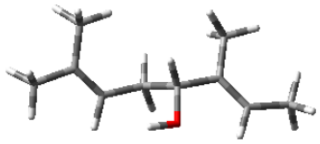

Conf.10 (1.15%)

10*S*-**7b**

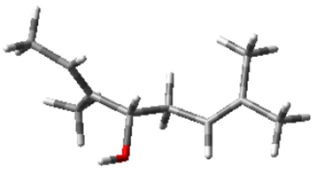

Conf.1 (12.04%)

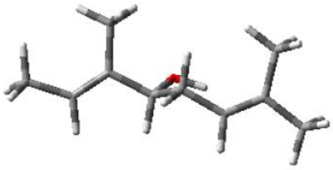

Conf.2 (26.85%)

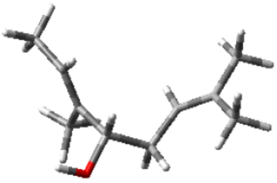

Conf.3 (13.71%)

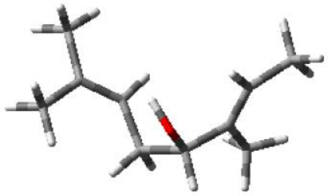

Conf.4 (4.48%)

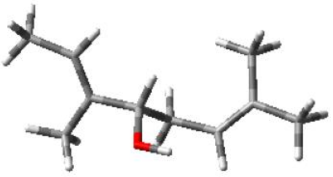

Conf.5 (2.83%)

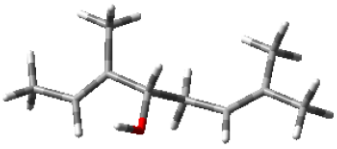

Conf.6 (3.13%)

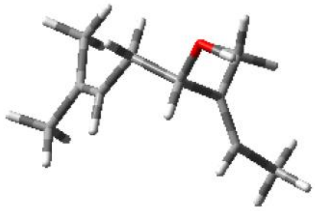

Conf.7 (13.58%)

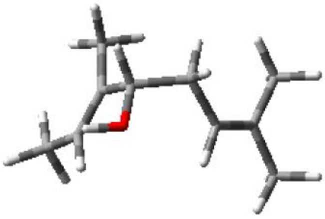

Conf.8 (1.17%)

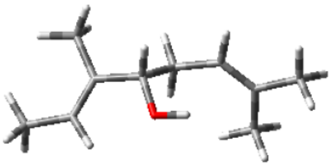

Conf.9 (9.92%)

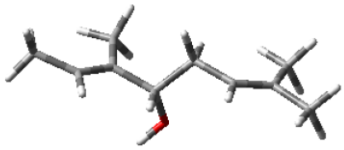

Conf.10 (12.29%)

Figure S3. The optimized conformers and equilibrium populations of 7**a**/**7b**.

Table S7. ^{1}H NMR Data of **3A** and **3B** (CD_3OD, 700MHz)

Pos.	**3A**	**3B**	$\Delta\boldsymbol{\delta}_{S\text{-}R}$
8	5.19	5.17	+0.02
9	3.44	3.43	+0.01
10	5.45	5.37	+0.08
13	1.32	1.39	-0.07
14	1.67	1.47	+0.20
15	1.02	0.98	+0.04

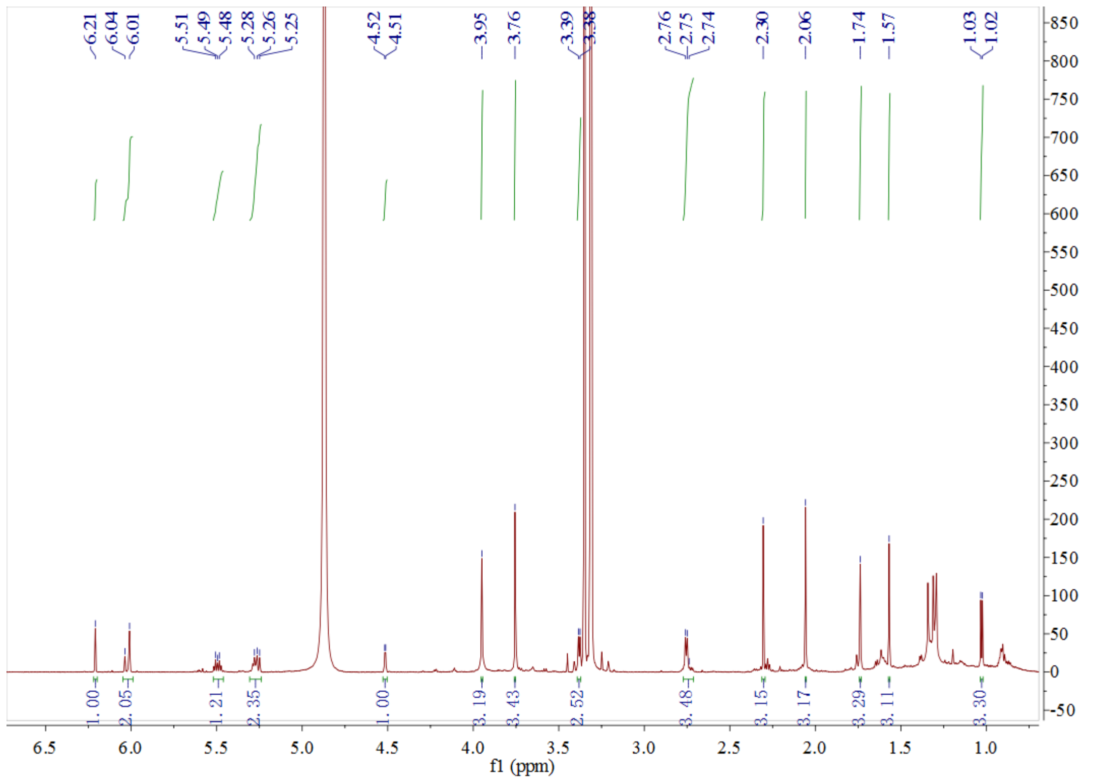

Figure S4. ^{1}H NMR spectrum of piericidin L (**1**) (CD_3OD, 700MHz)

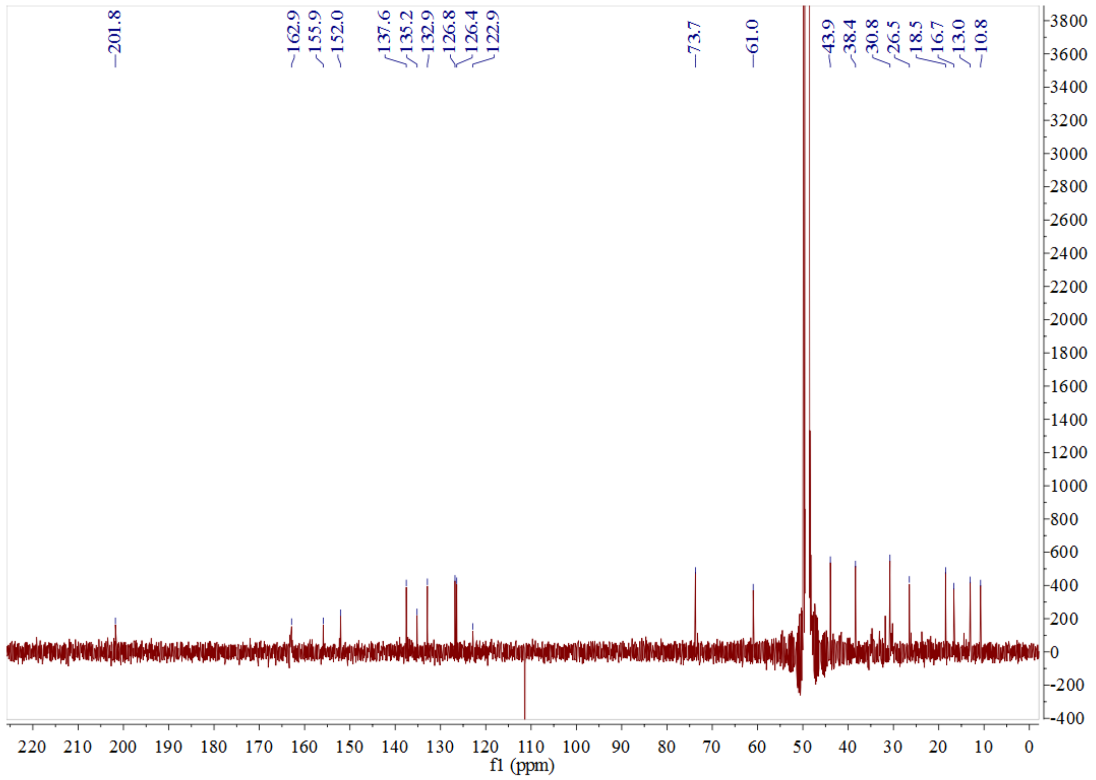

Figure S5. ^{13}C NMR spectrum of piericidin L (**1**) (CD_3OD, 175MHz)

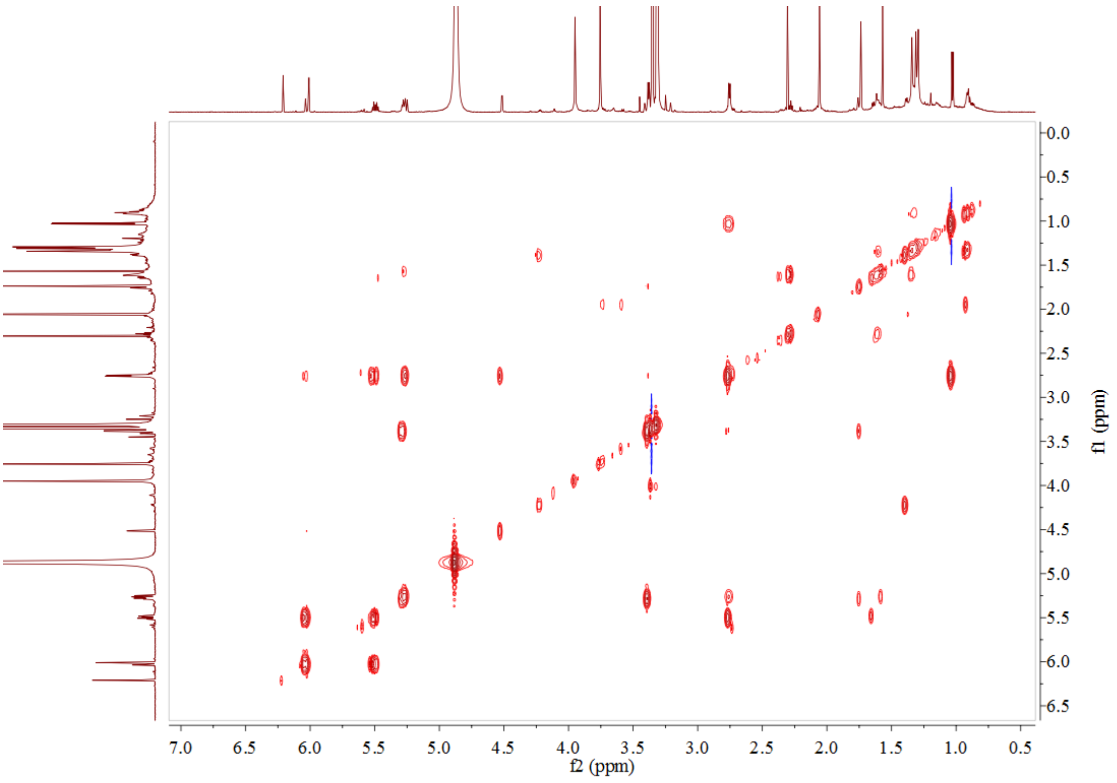

Figure S6. ^{1}H-^{1}HCOSY spectrum of piericidin L (**1**) (CD_3OD)

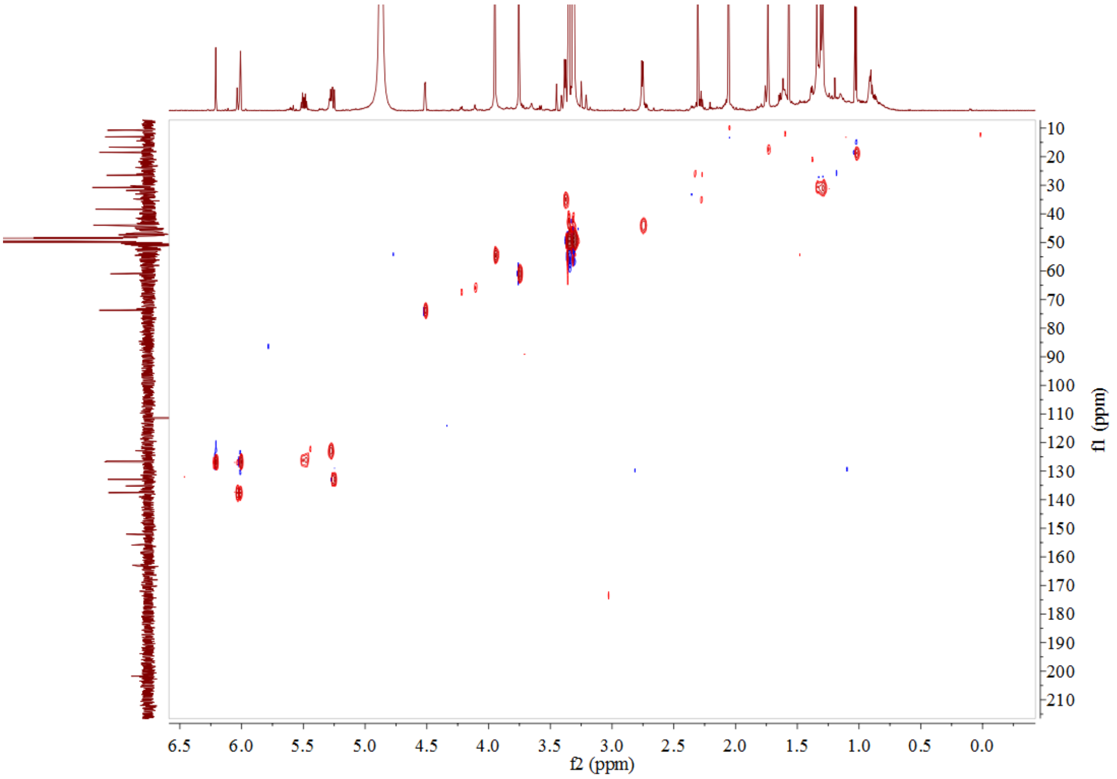

Figure S7. HSQC spectrum of piericidin L (**1**) (CD_3OD)

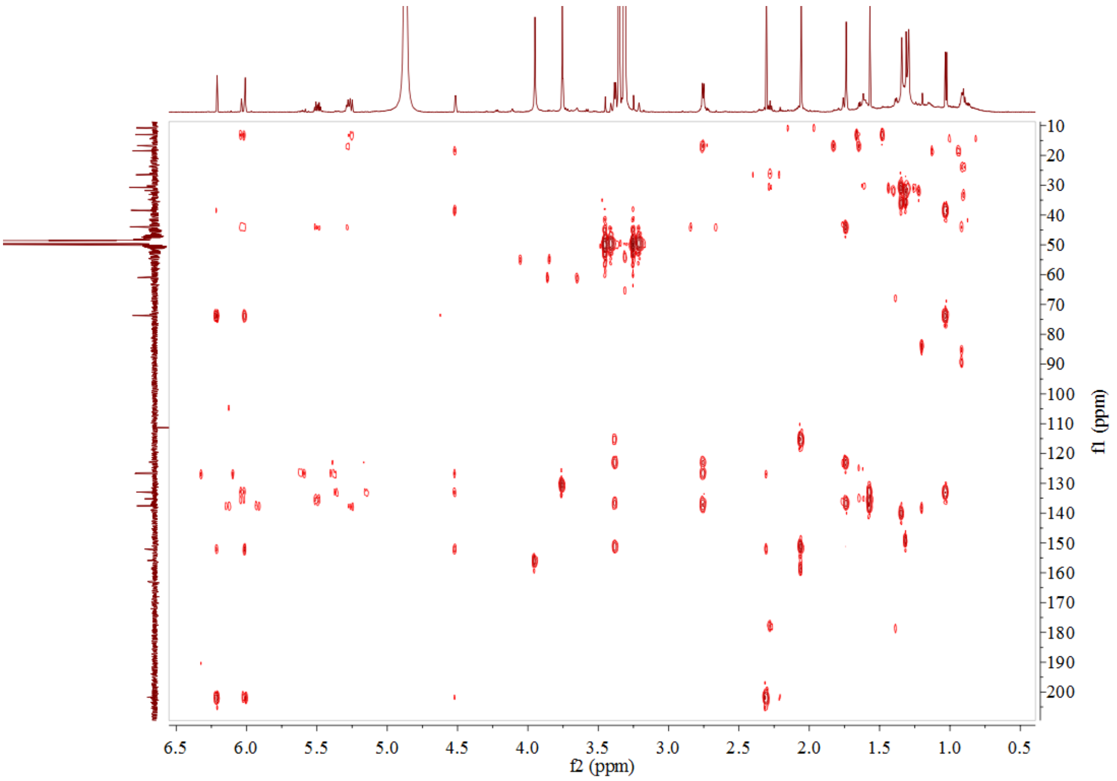

Figure S8. HMBC spectrum of piericidin L (**1**) (CD_3OD)

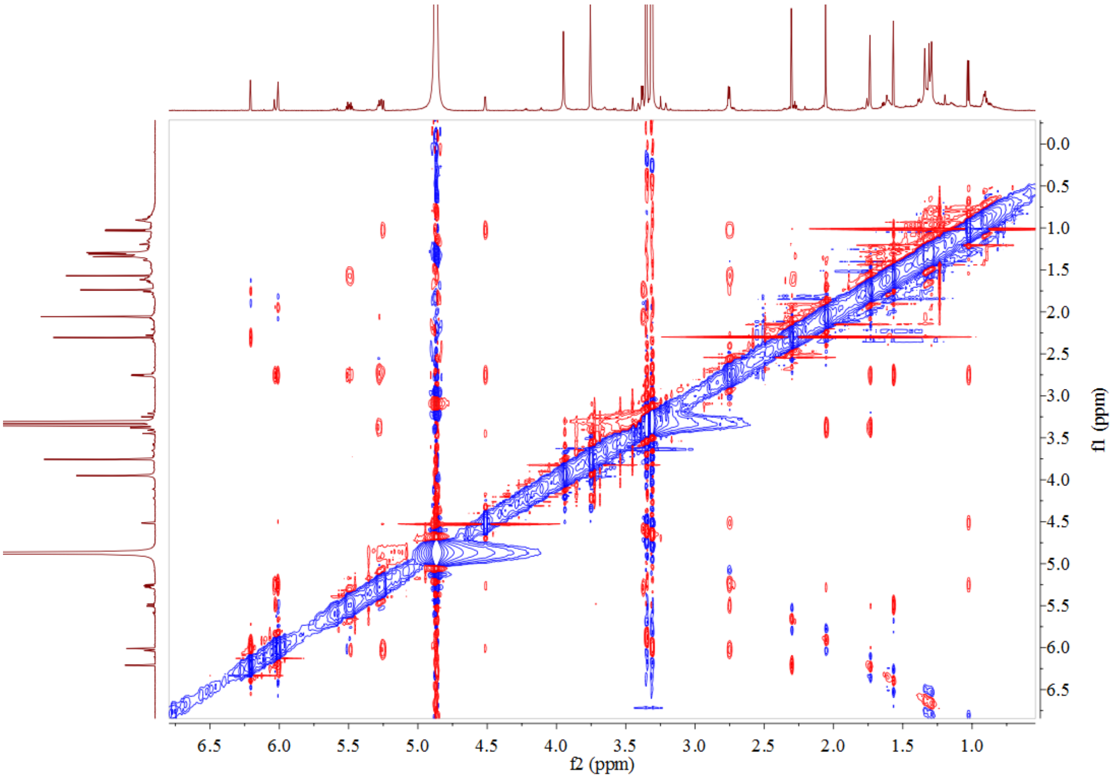

Figure S9. NOESY spectrum of piericidin L (**1**) (CD_3OD)

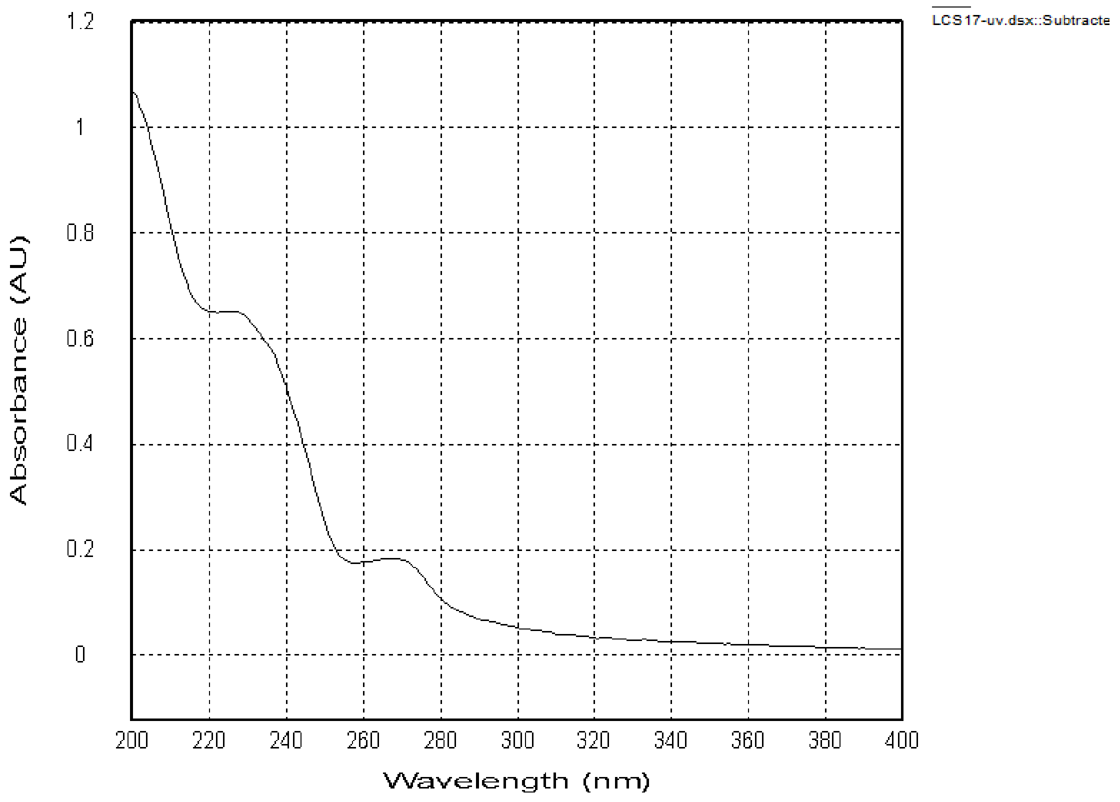

Figure S10. UV spectrum of piericidin L (**1**)

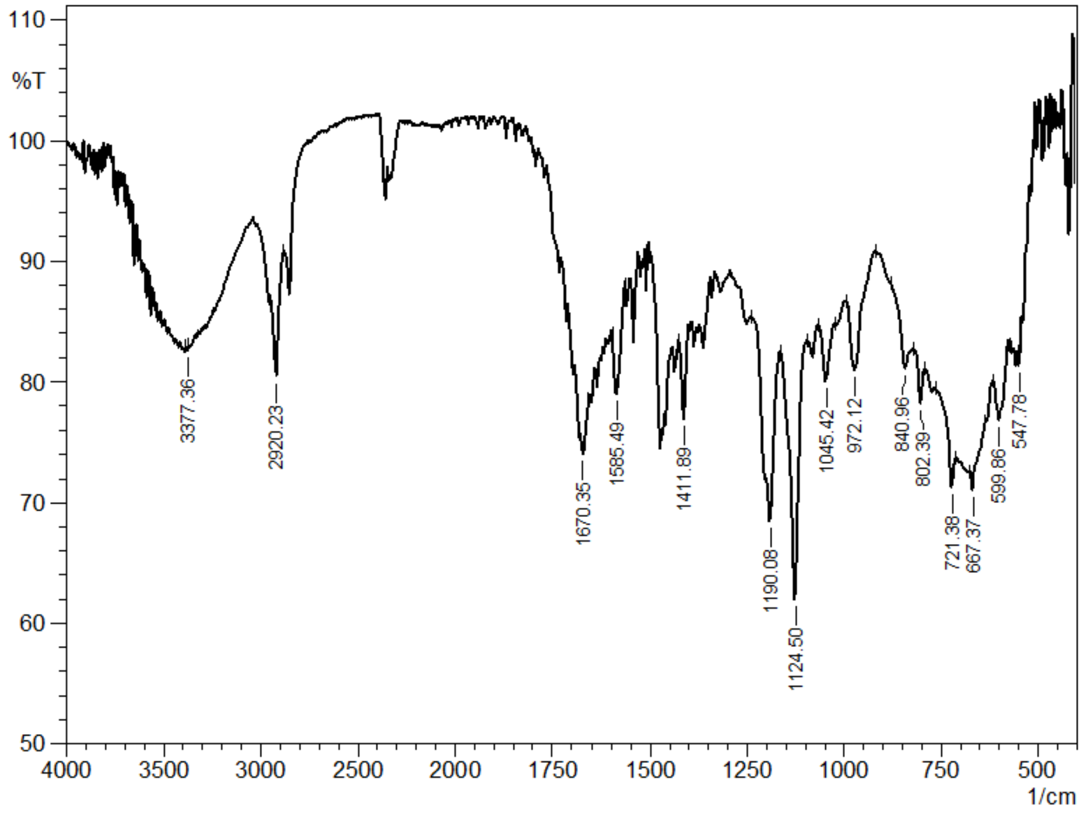

Figure S11. IR spectrum of piericidin L (**1**)

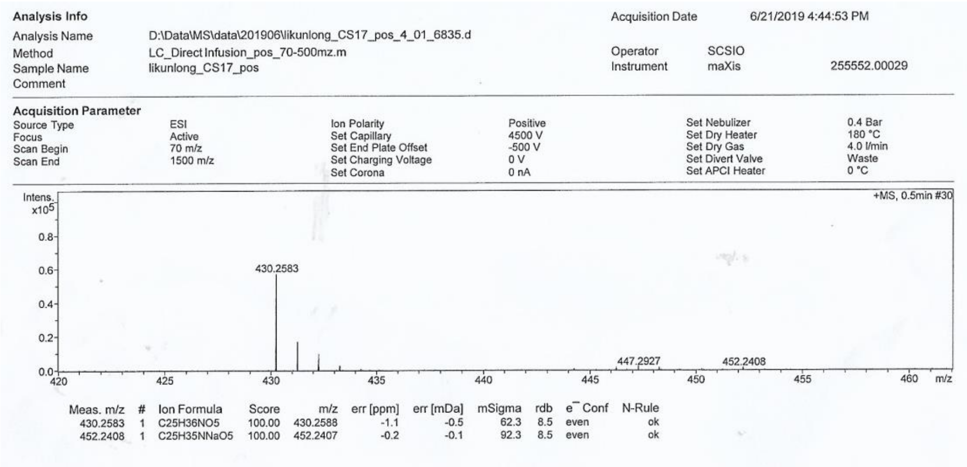

Figure S12. HRESIMS spectrum of piericidin (**1**)

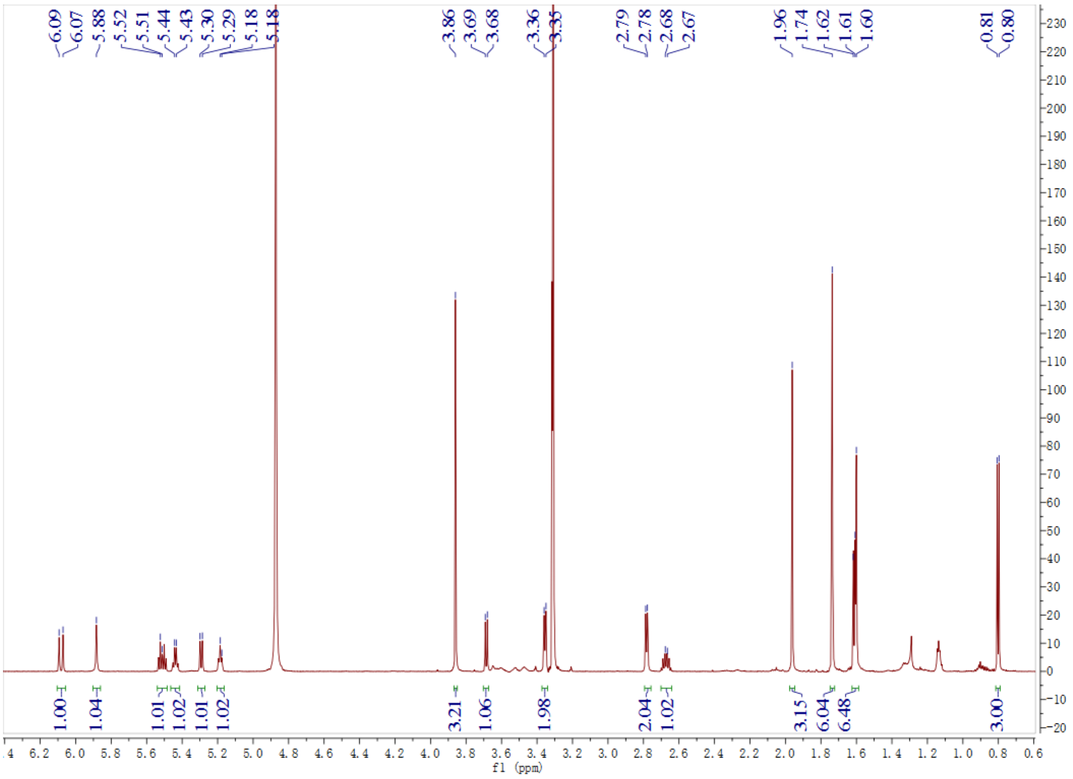

Figure S13. ^{1}H NMR spectrum of piericidin M (**2**) (CD_3OD, 700MHz)

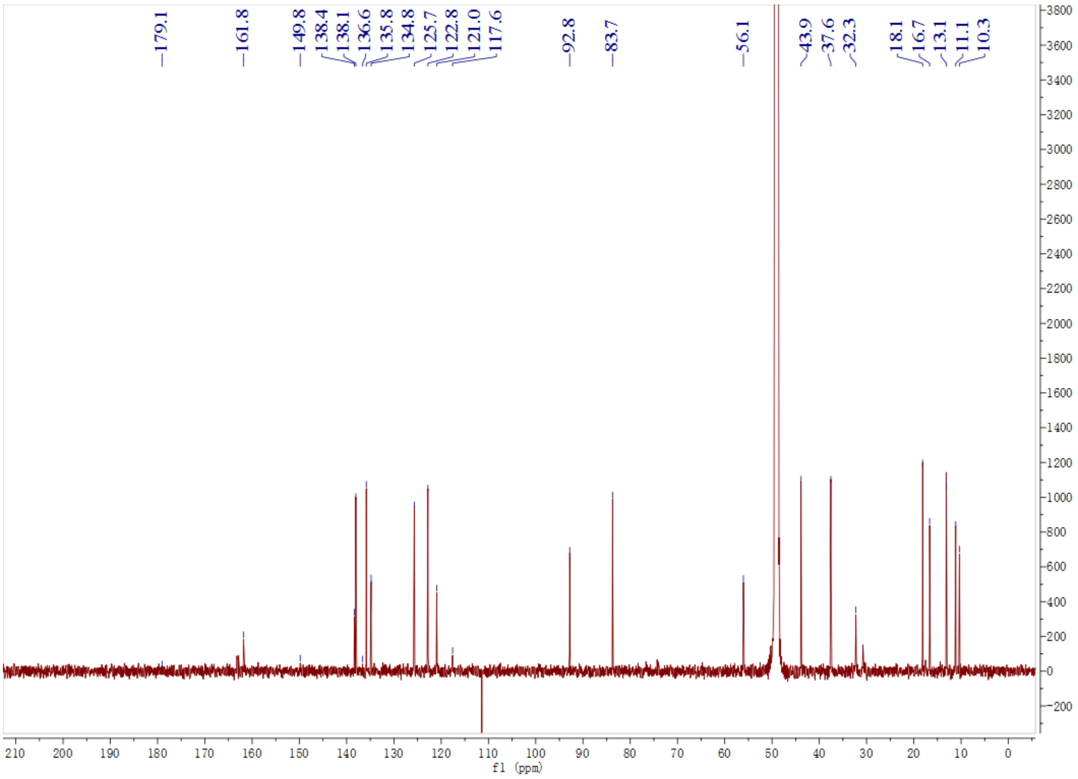

Figure S14. ^{13}C NMR spectrum of piericidin M (**2**) (CD_3OD, 175MHz)

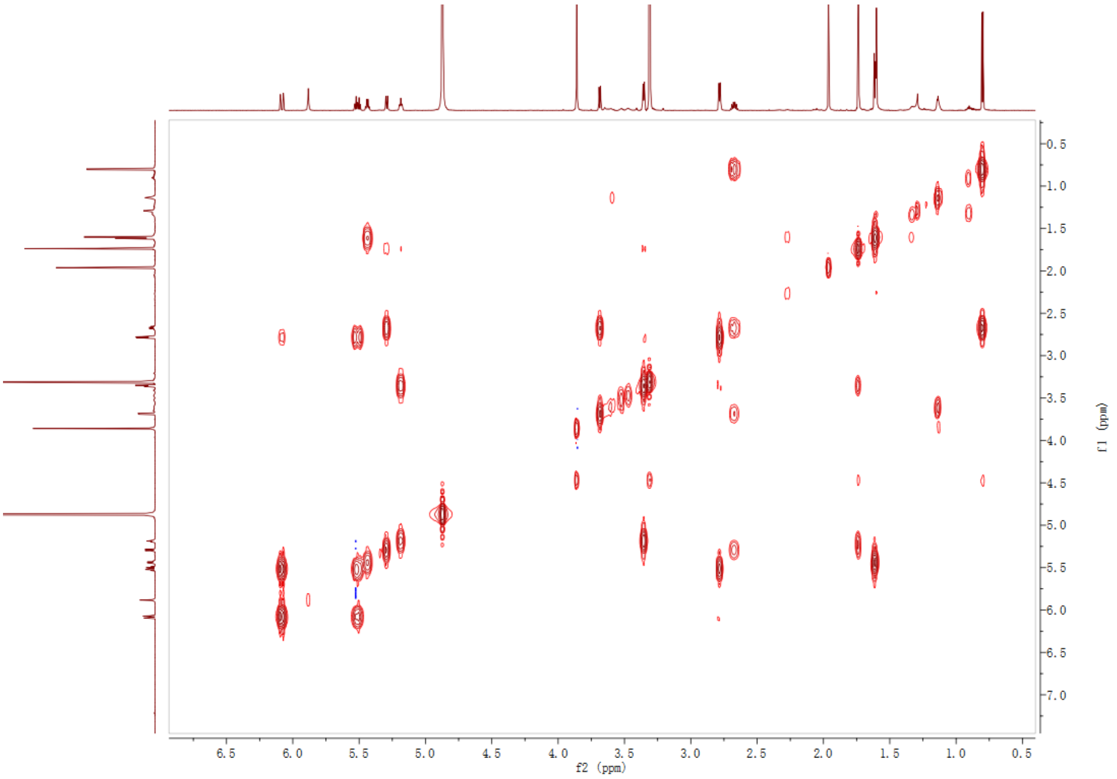

Figure S15. ^{1}H-^{1}H COSY spectrum of piericidin M (**2**) (CD_3OD)

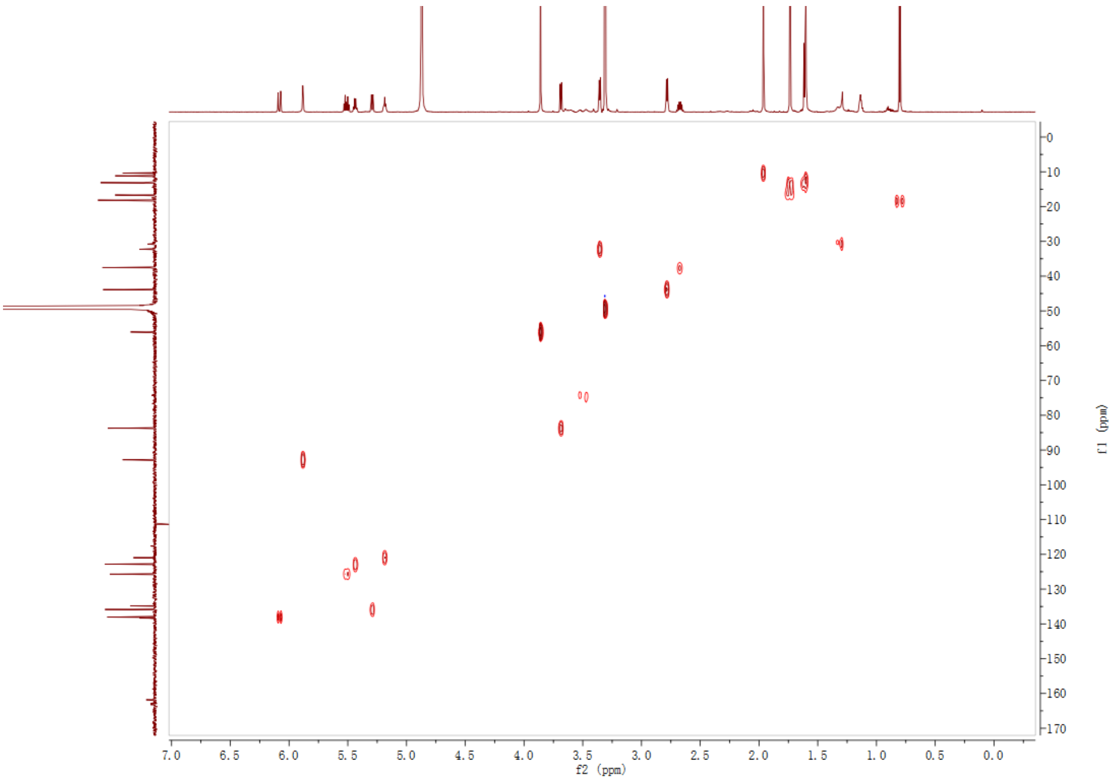

Figure S16. HSQC spectrum of piericidin M (**2**) (CD_3OD)

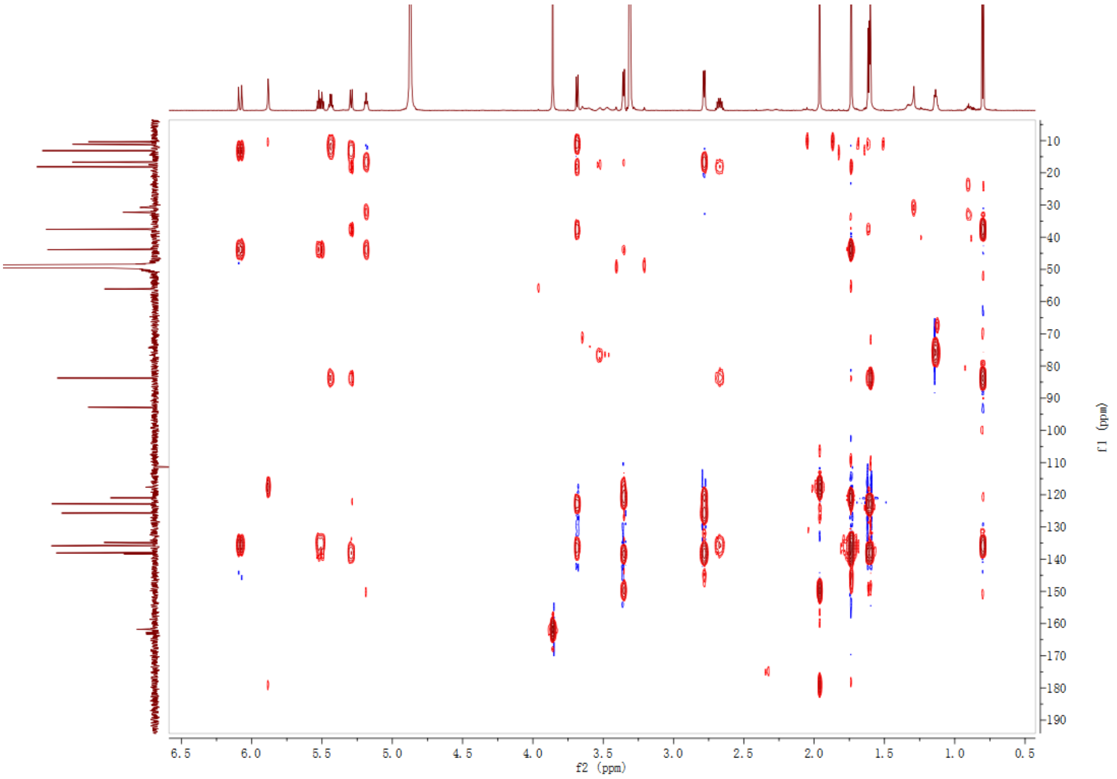

Figure S17. HMBC spectrum of piericidin M (**2**) (CD_3OD)

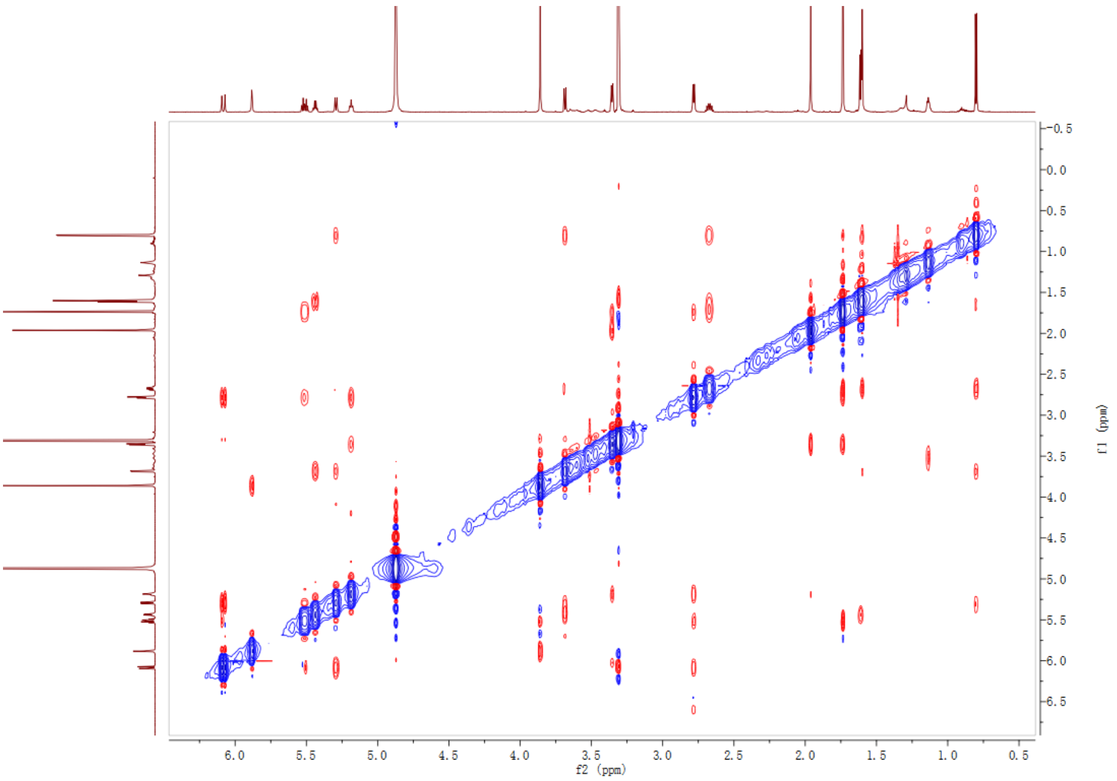

Figure S18. NOESY spectrum of piericidin M (**2**) (CD_3OD)

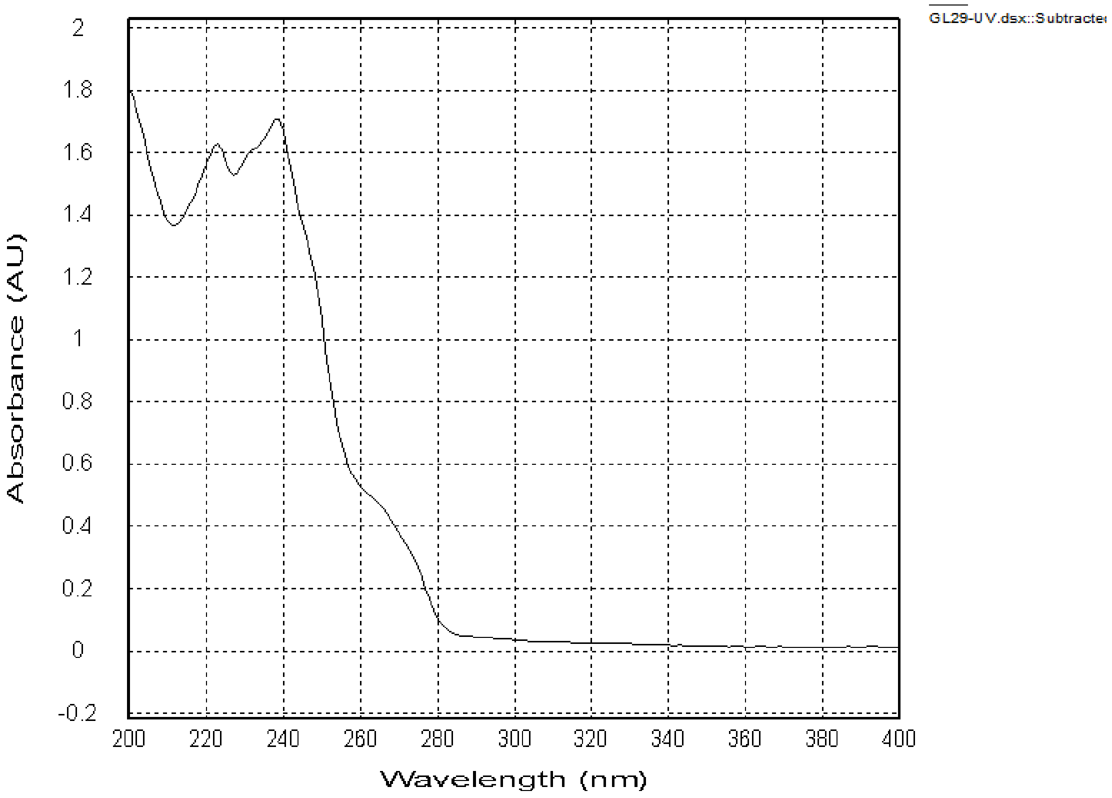

Figure S19. UV spectrum of piericidin M (**2**)

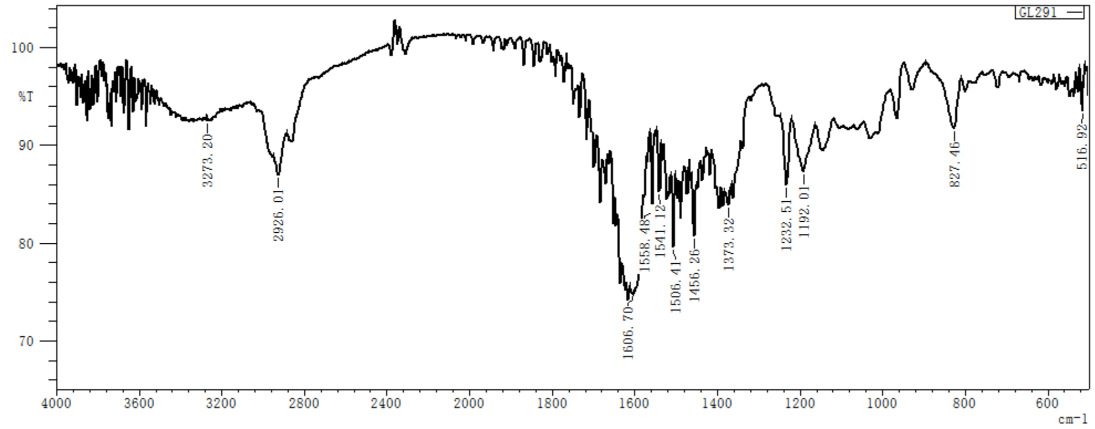

Figure S20. IR spectrum of piericidin M (**2**)

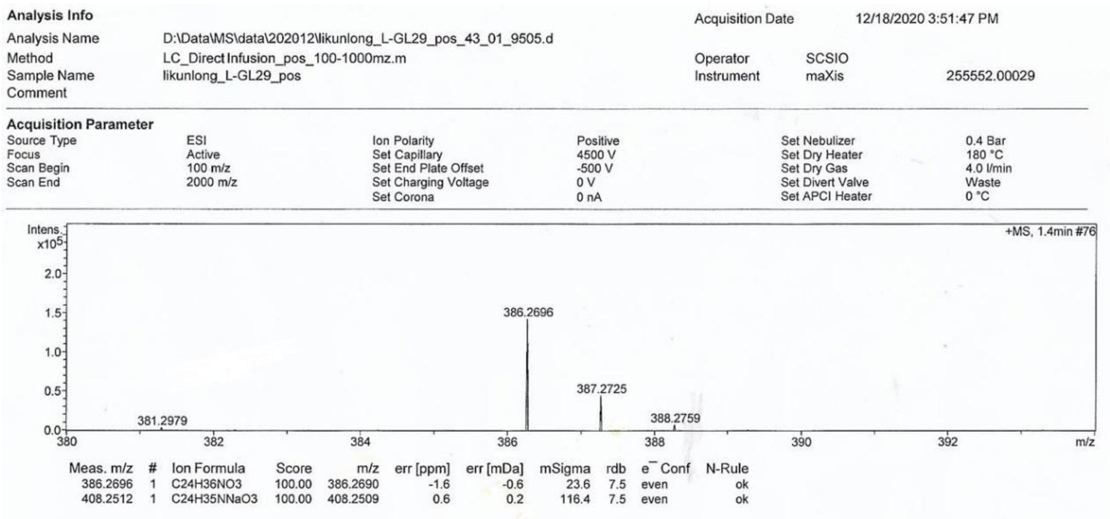

Analysis Info

Analysis Name	D:\Data\MS\data\202012\likunlong_L-GL29_pos_43_01_9505.d	Acquisition Date	12/18/2020 3:51:47 PM
Method	LC_Direct Infusion_pos_100-1000mz.m	Operator	SCSIO
Sample Name	likunlong_L-GL29_pos	Instrument	maXis 255552.00029
Comment			

Acquisition Parameter

Source Type	ESI	Ion Polarity	Positive	Set Nebulizer	0.4 Bar
Focus	Active	Set Capillary	4500 V	Set Dry Heater	180 °C
Scan Begin	100 m/z	Set End Plate Offset	-500 V	Set Dry Gas	4.0 l/min
Scan End	2000 m/z	Set Charging Voltage	0 V	Set Divert Valve	Waste
		Set Corona	0 nA	Set APCI Heater	0 °C

Meas. m/z	#	Ion Formula	Score	m/z	err [ppm]	err [mDa]	mSigma	rdb	e⁻ Conf	N-Rule
386.2696	1	C24H36NO3	100.00	386.2690	-1.6	-0.6	23.6	7.5	even	ok
408.2512	1	C24H35NNaO3	100.00	408.2509	0.6	0.2	116.4	7.5	even	ok

Figure S21. HRESIMS spectrum of piericidin M (**2**)

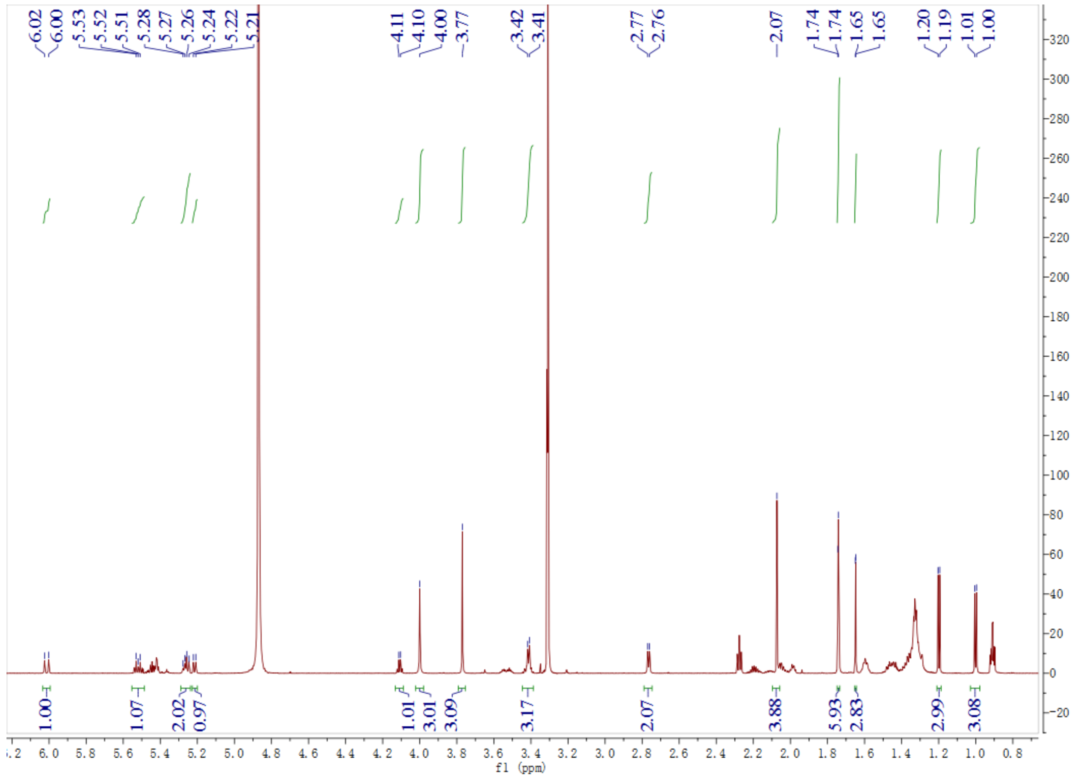

Figure S22. ^{1}H NMR spectrum of piericidin N (**3**) (CD_3OD, 700MHz)

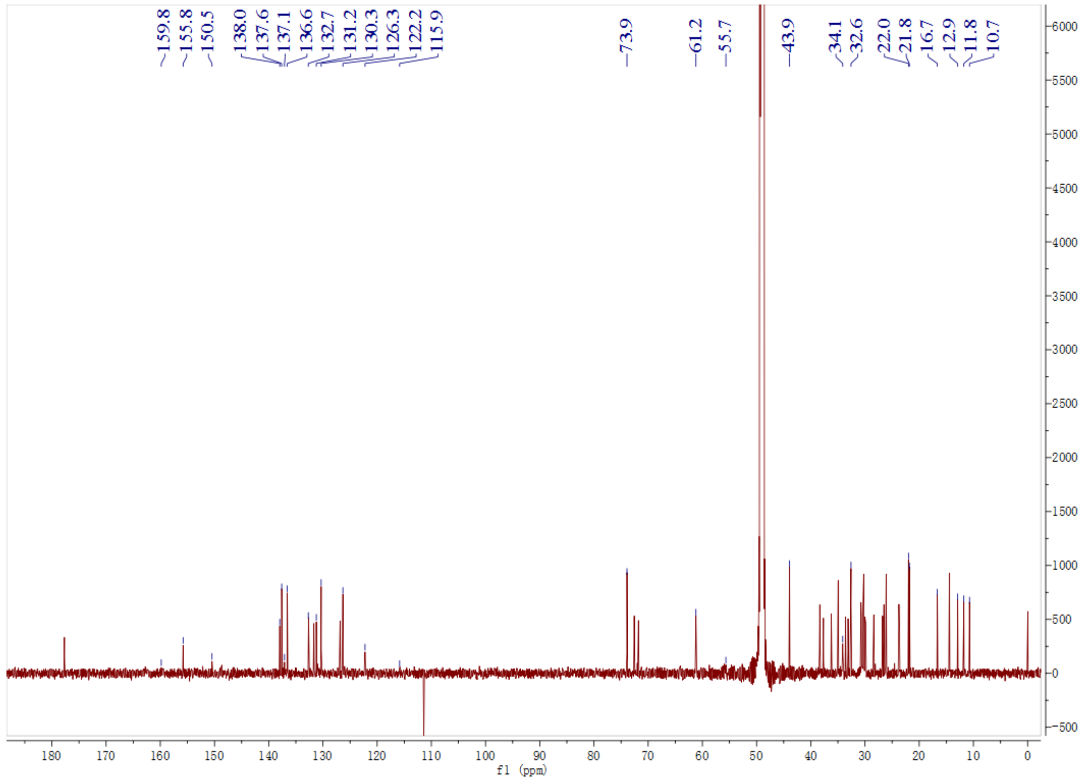

Figure S23. ^{13}C NMR spectrum of piericidin N (**3**) (CD_3OD, 175MHz)

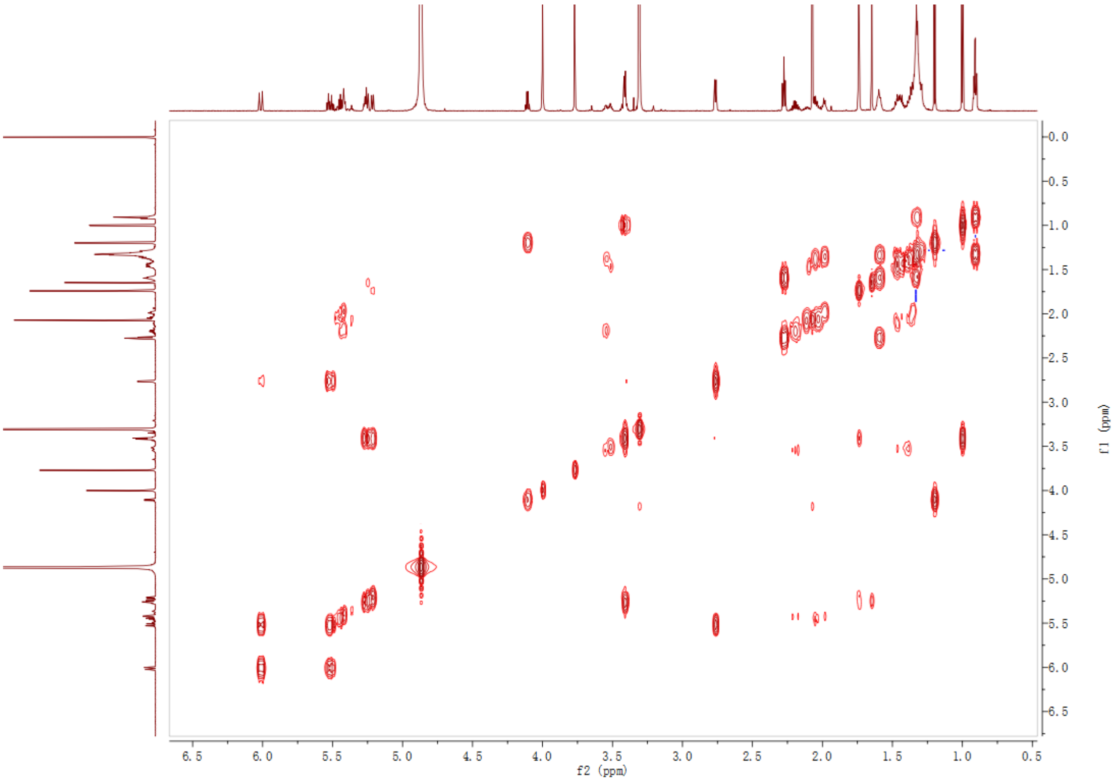

Figure S24. ^{1}H-^{1}H COSY spectrum of piericidin N (**3**) (CD_3OD)

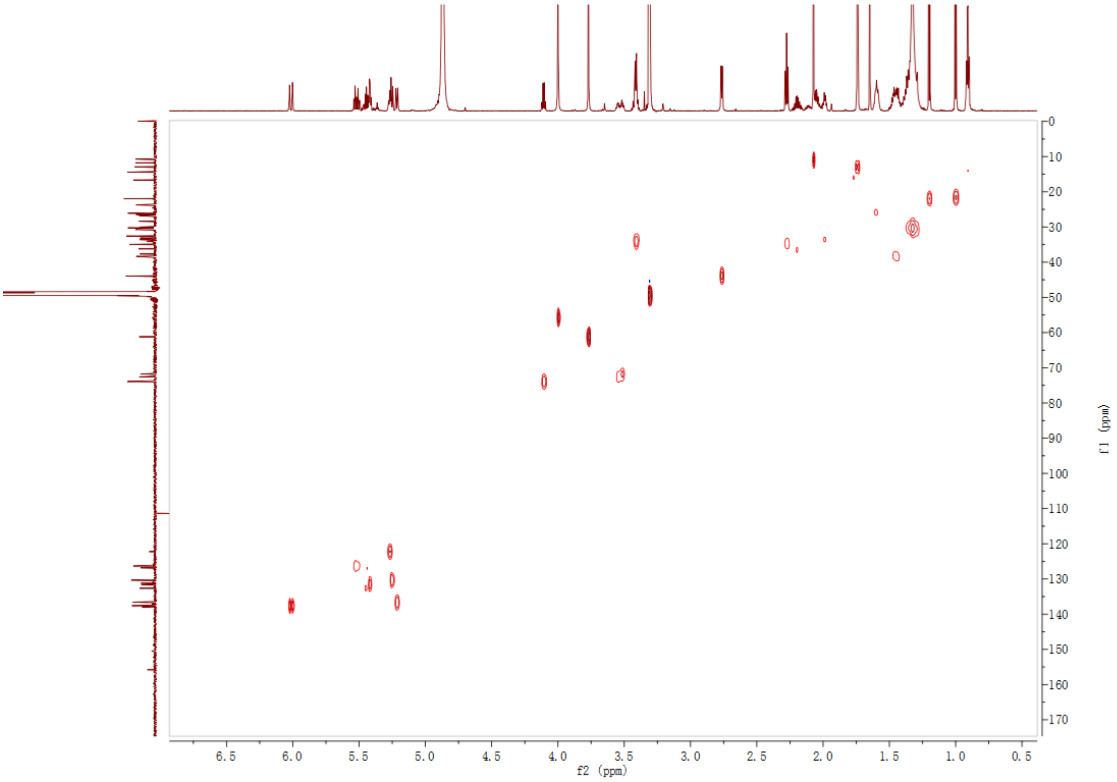

Figure S25. HSQC spectrum of piericidin N (**3**) (CD_3OD)

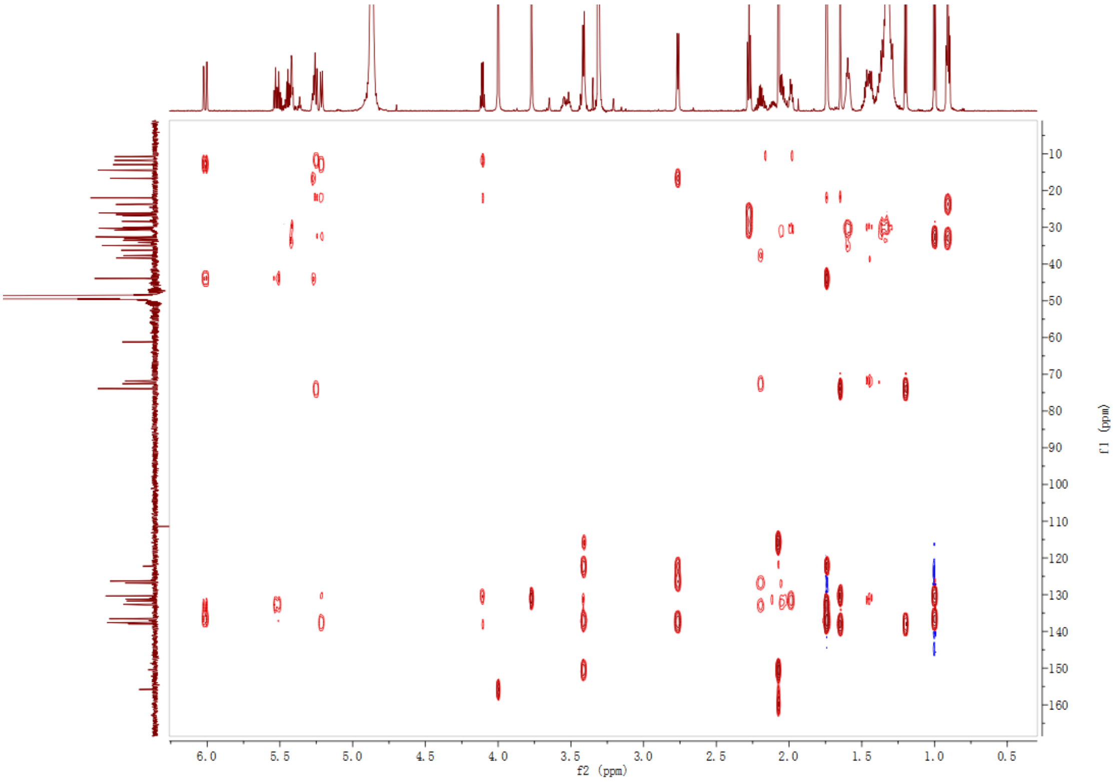

Figure S26. HMBC spectrum of piericidin N (**3**) (CD_3OD)

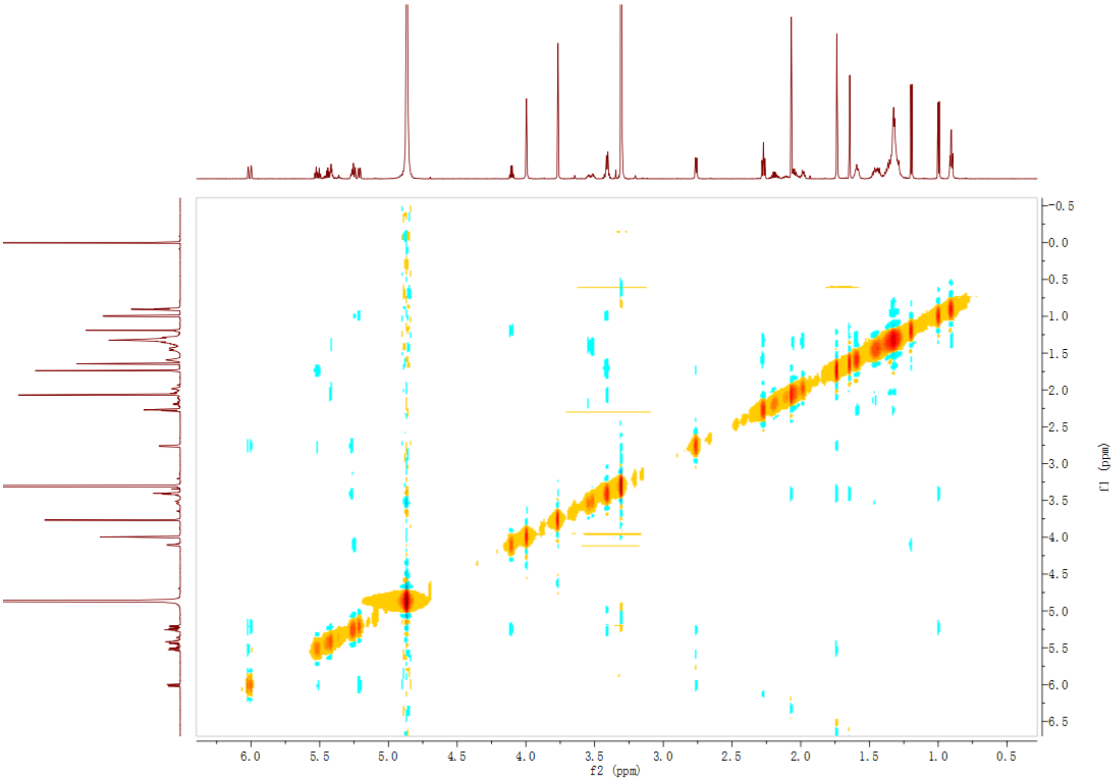

Figure S27. NOESY spectrum of piericidin N (**3**) (CD_3OD)

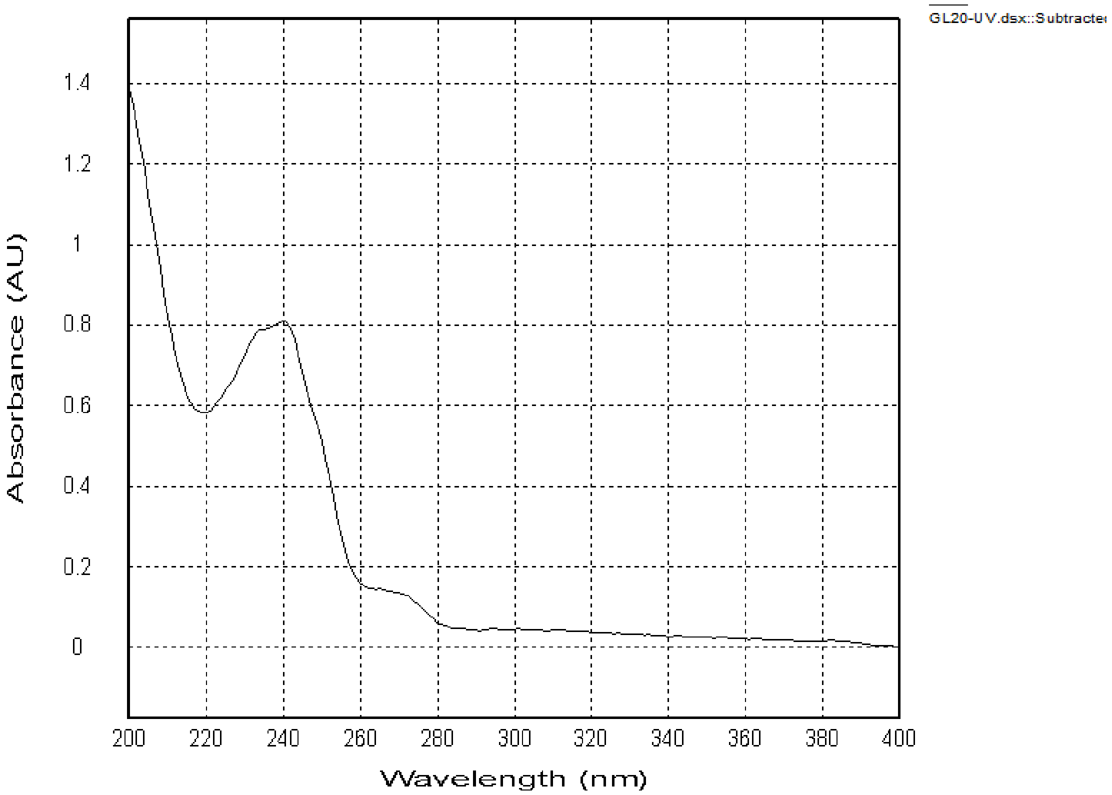

Figure S28. UV spectrum of piericidin N (**3**)

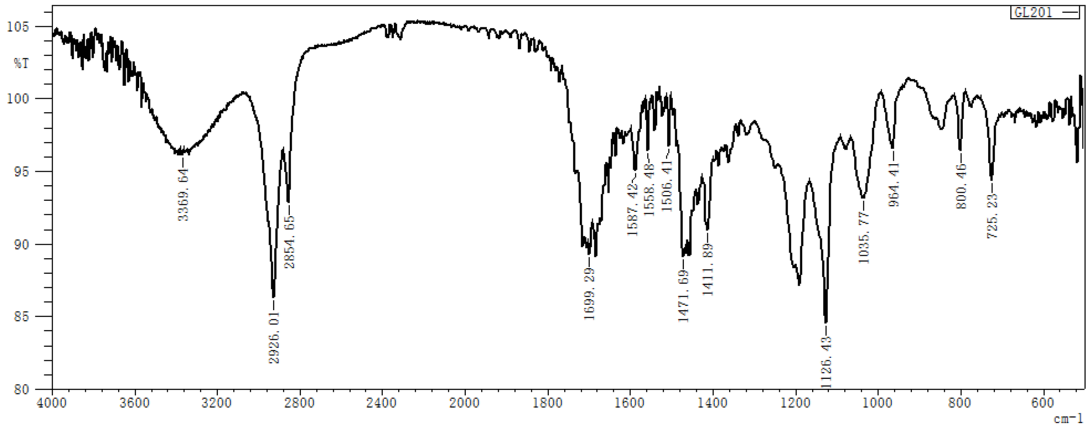

Figure S29. IR spectrum of piericidin N (**3**)

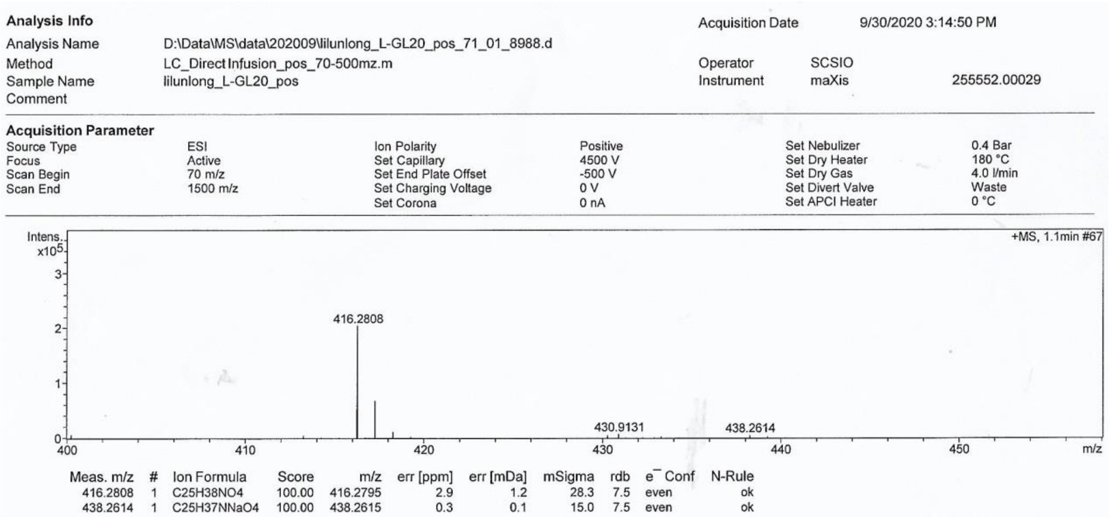

Figure S30. HRESIMS spectrum of piericidin N (**3**)

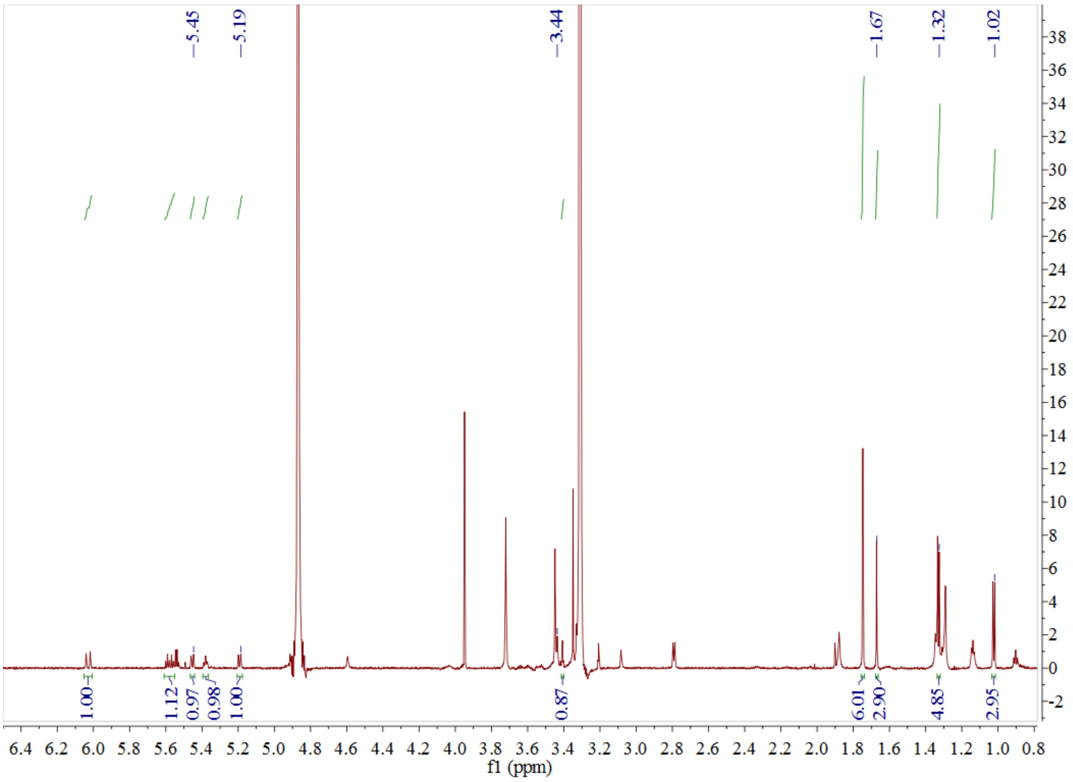

Figure S31. ^{1}H NMR spectrum of **3A** (CD_3OD, 700MHz)

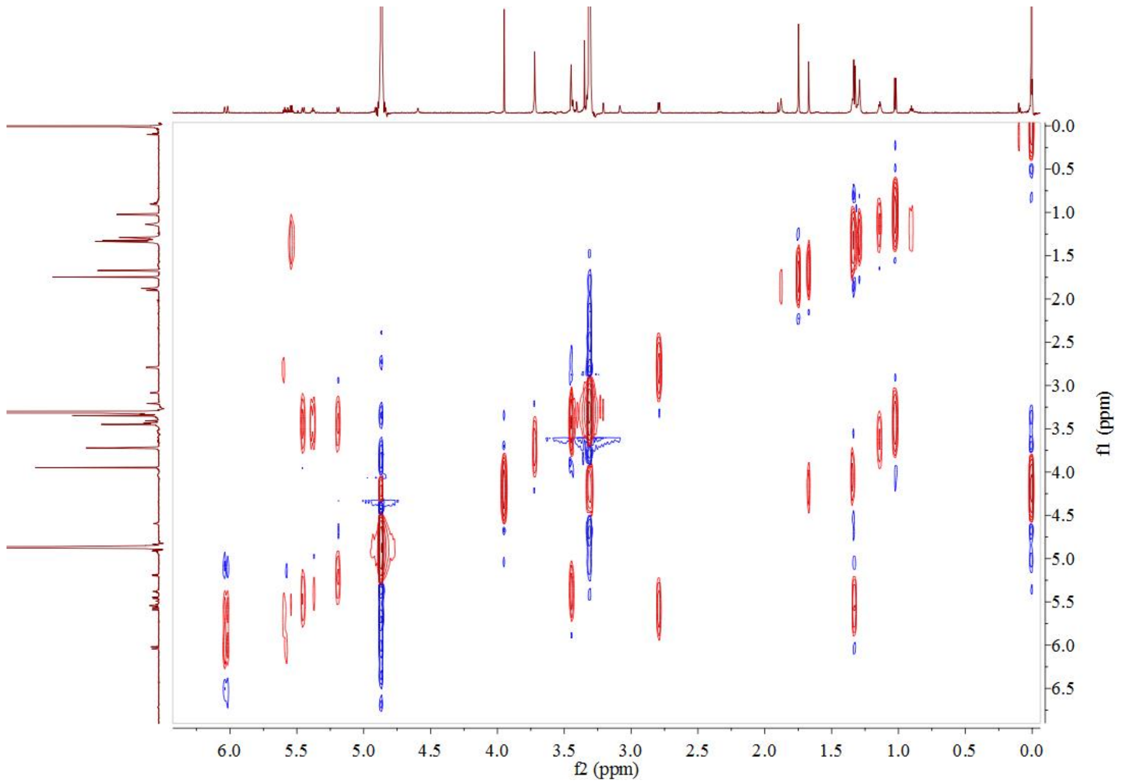

Figure S32. ^{1}H-^{1}H COSY spectrum of **3A** (CD_3OD)

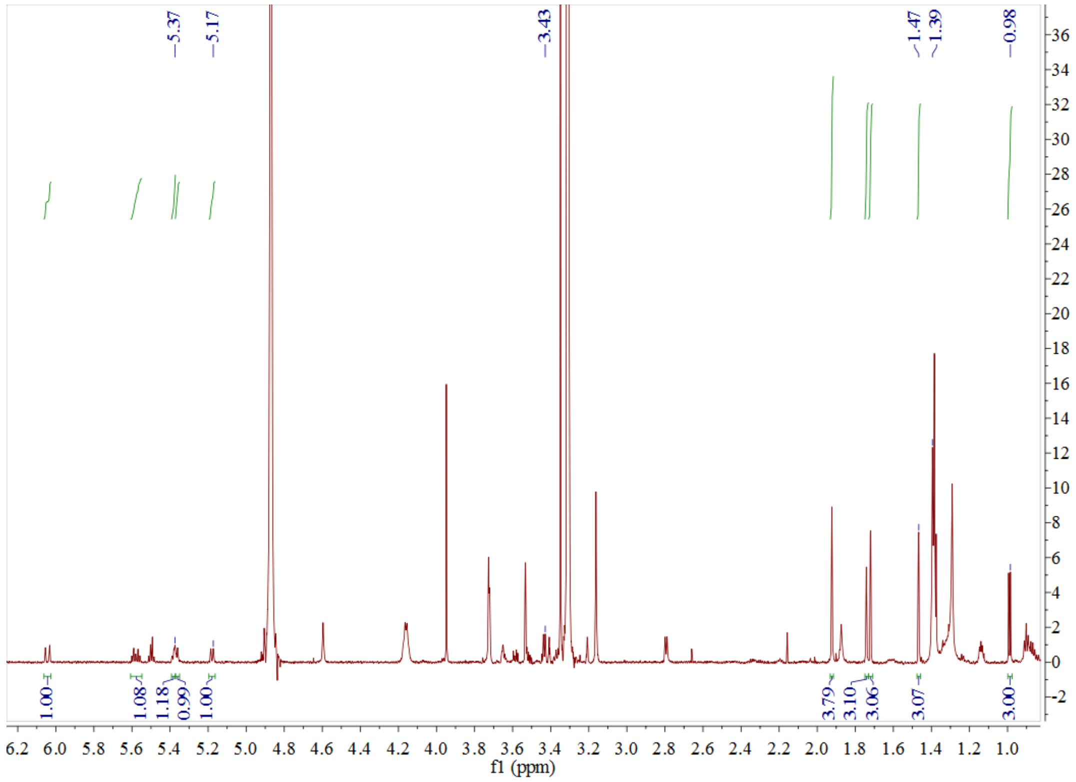

Figure S33. ^{1}H NMR spectrum of **3B** (CD_3OD, 700MHz)

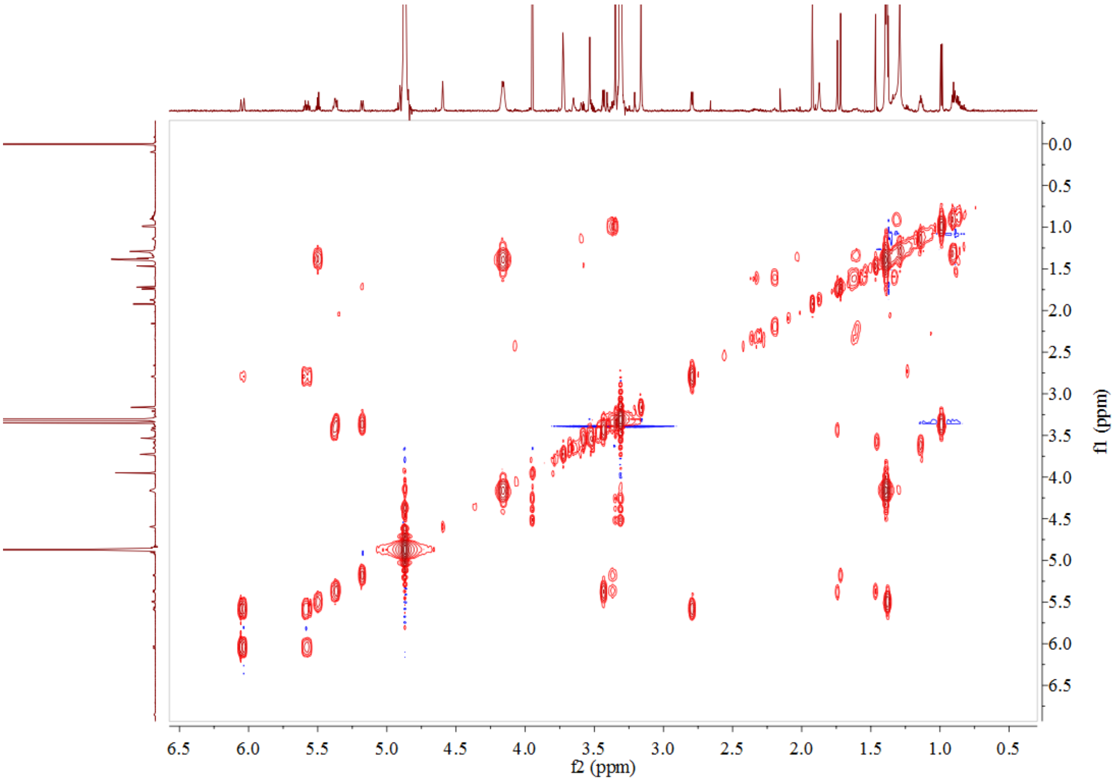

Figure S34. ^{1}H-^{1}H COSY spectrum of **3B** (CD_3OD)

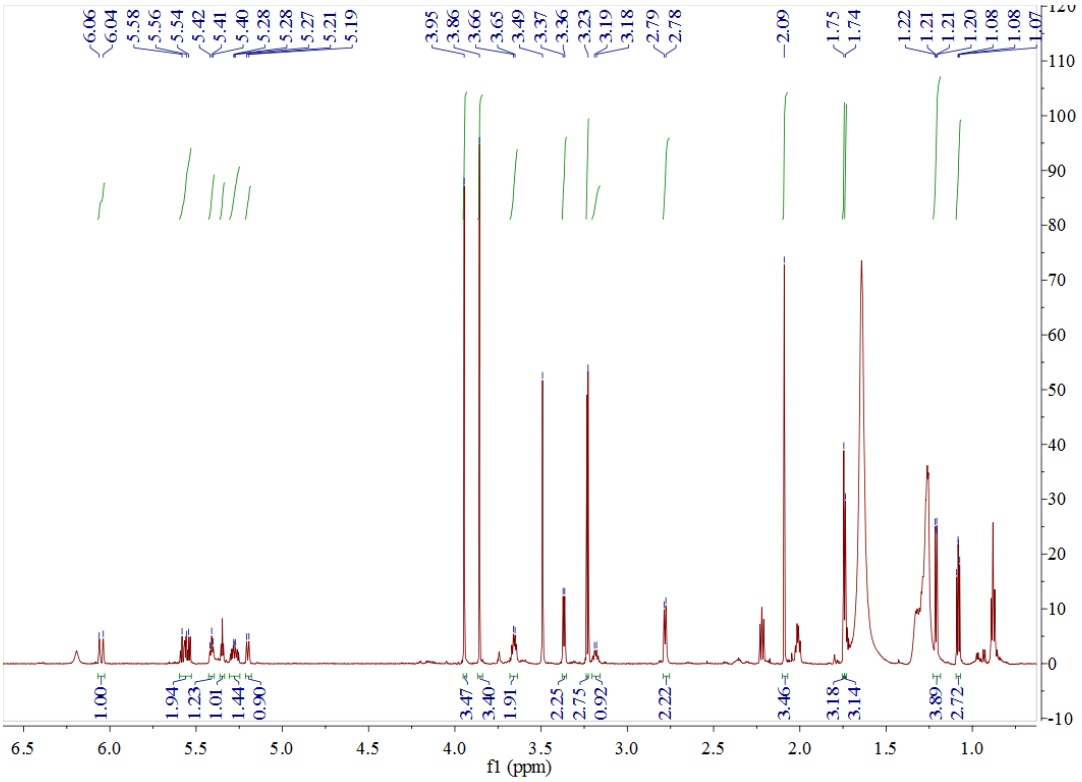

Figure S35. ^{1}H NMR spectrum of piericidin O (**4**) (CD_3OD, 700MHz)

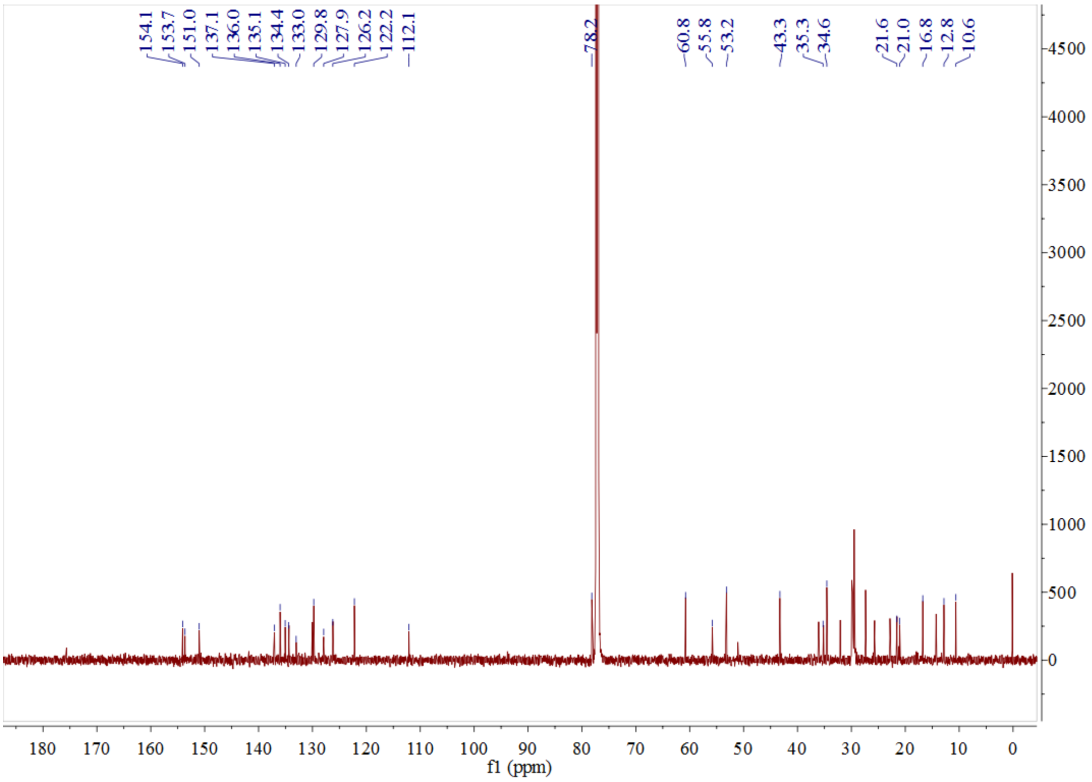

Figure S36. ^{13}C NMR spectrum of piericidin O **(4)** (CD_3OD, 175MHz)

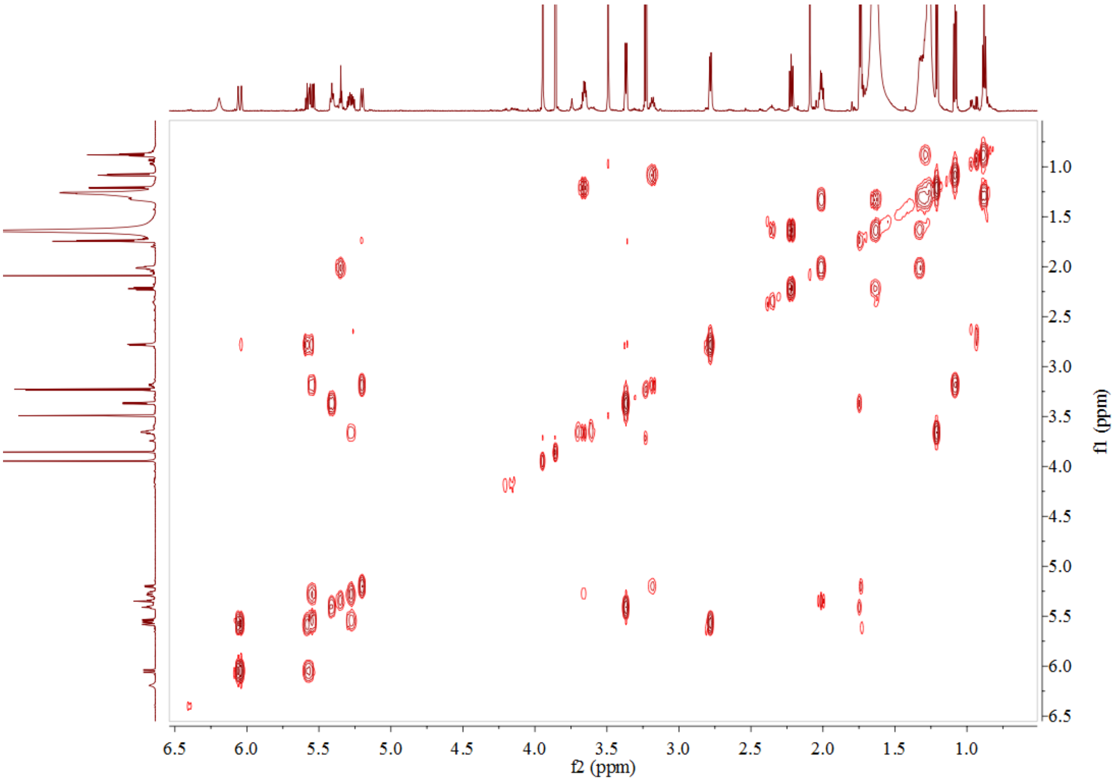

Figure S37. ^{1}H-^{1}H COSY spectrum of piericidin O **(4)** (CD_3OD)

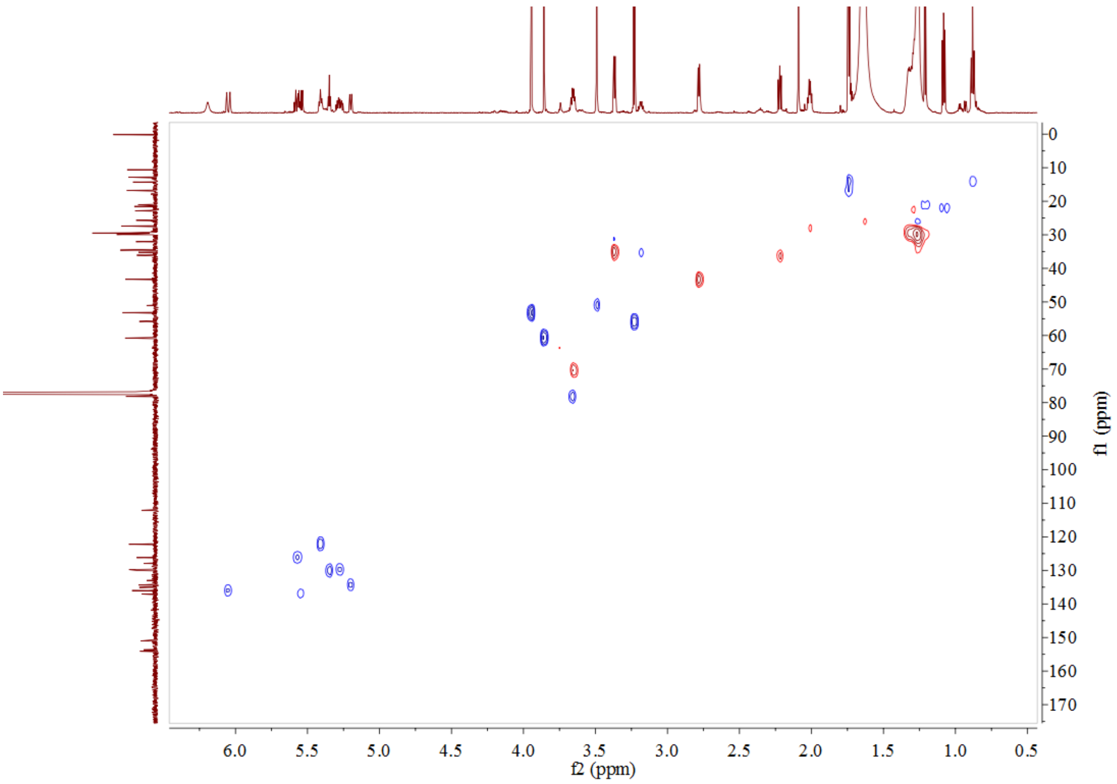

Figure S38. HSQC spectrum of piericidin O (**4**) (CD_3OD)

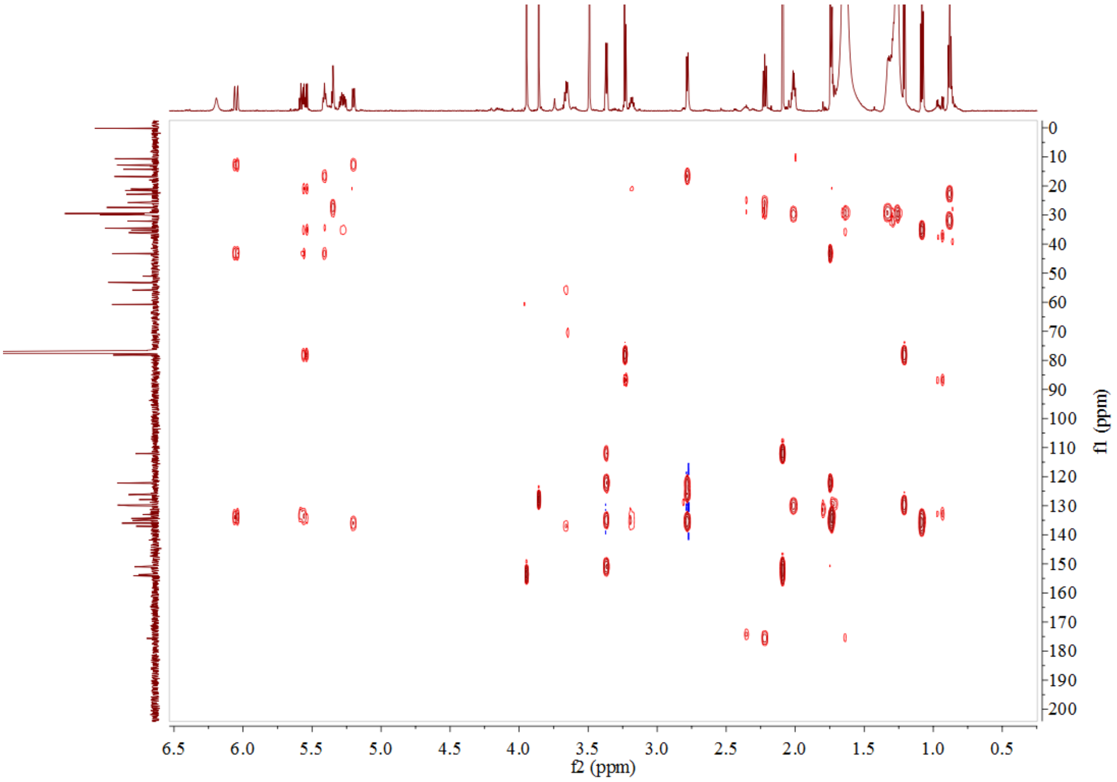

Figure S39. HMBC spectrum of piericidin O (**4**) (CD_3OD)

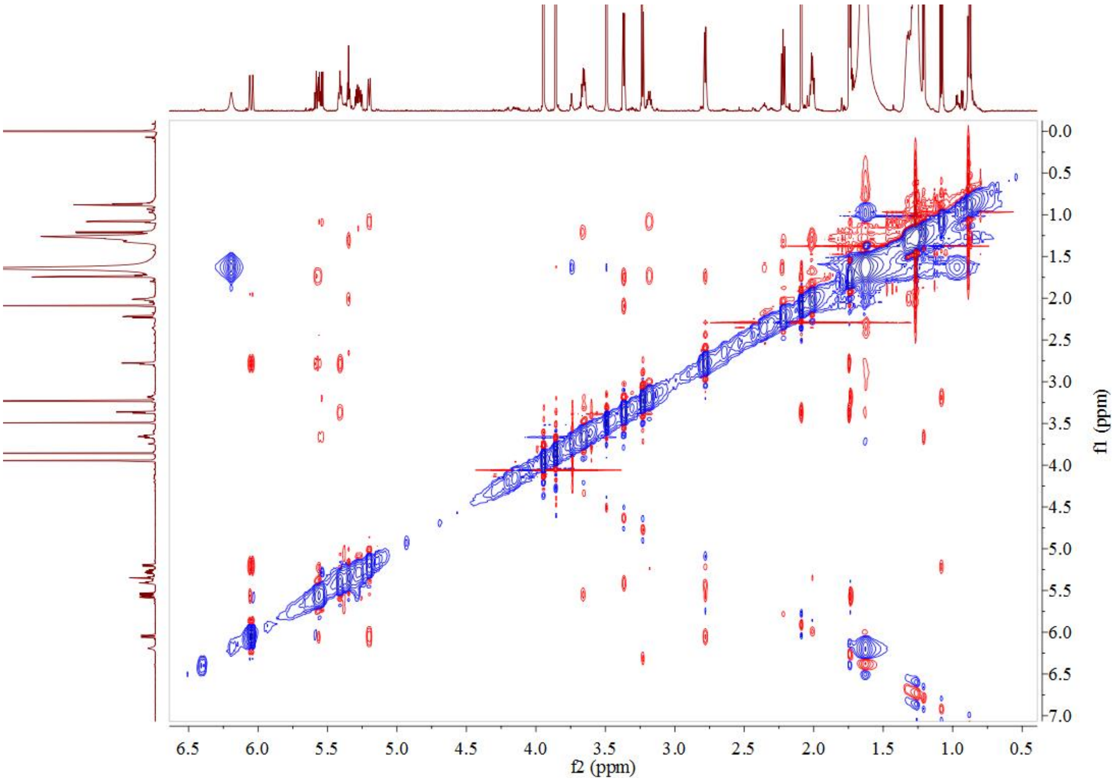

Figure S40. NOESY spectrum of piericidin O (**4**) (CD_3OD)

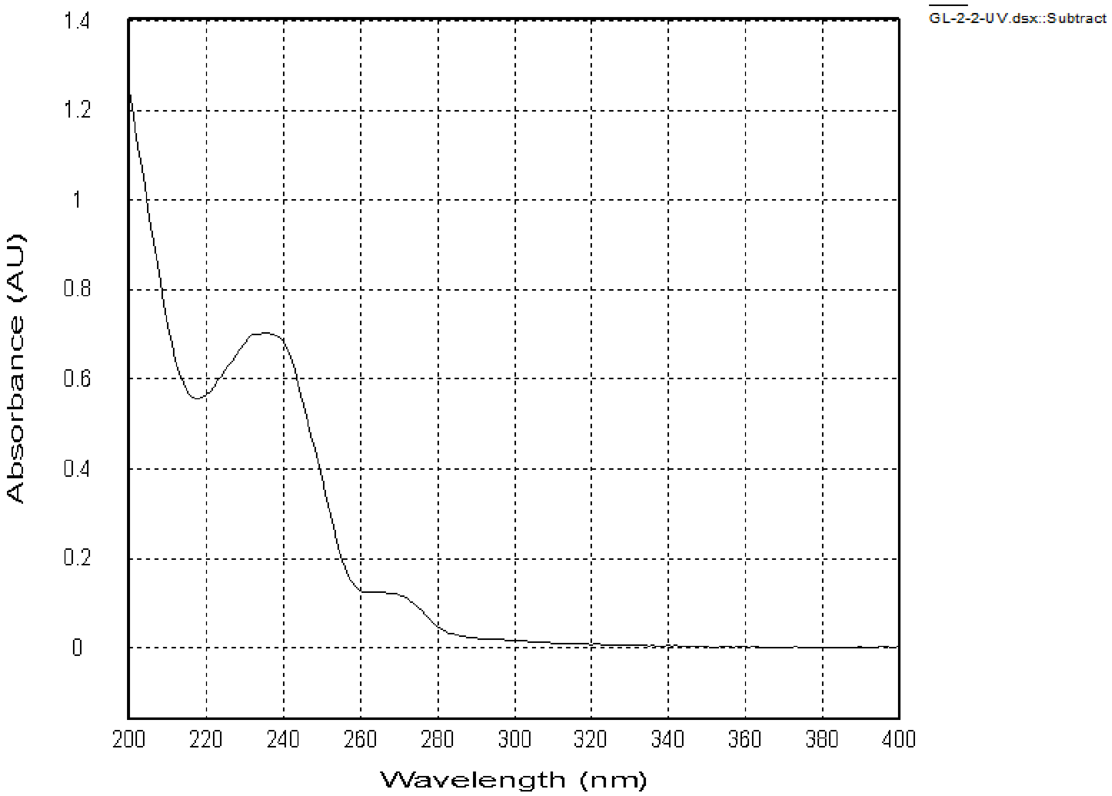

Figure S41. UV spectrum of piericidin O (**4**)

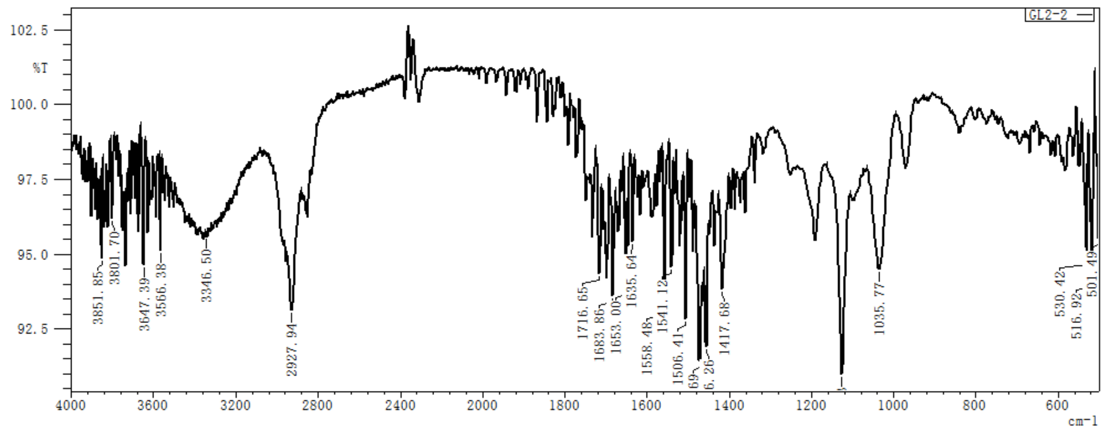

Figure S42. IR spectrum of piericidin O (**4**)

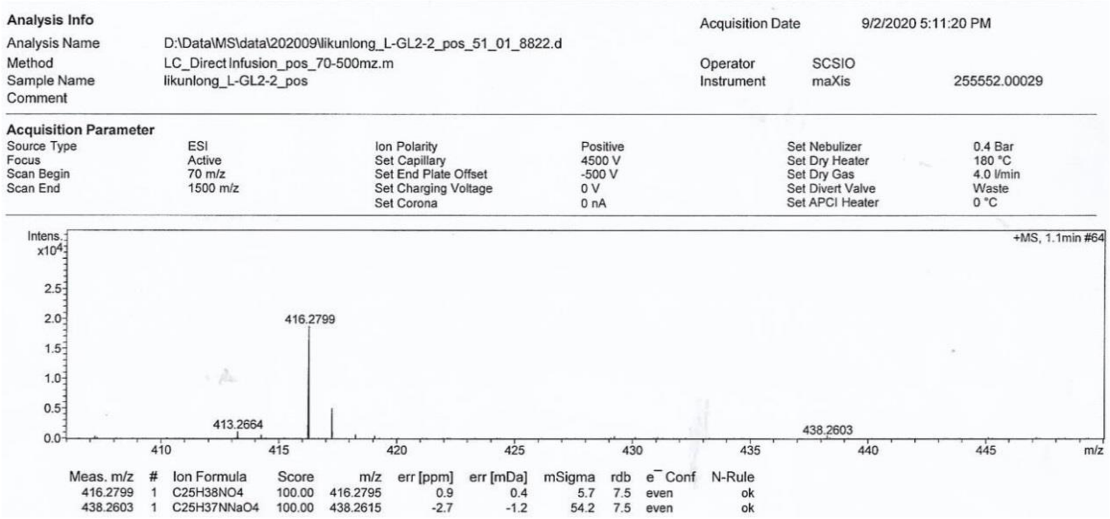

Figure S43. HRESIMS spectrum of piericidin O (**4**)

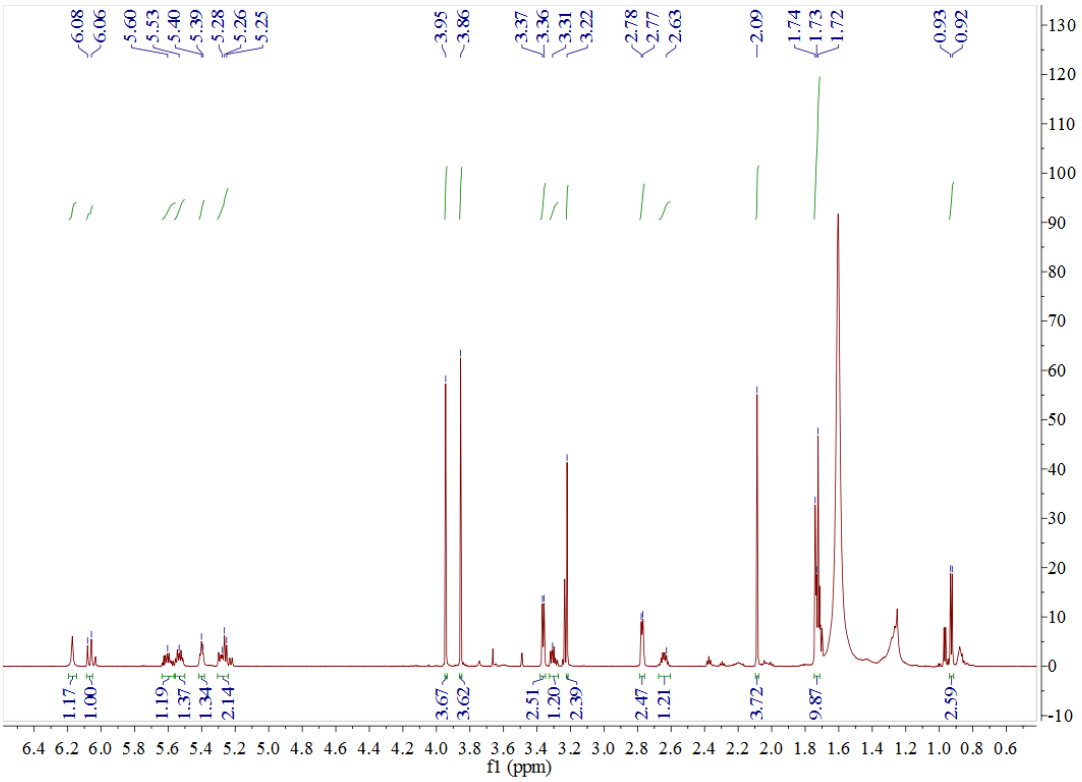

Figure S44. ^{1}H NMR spectrum of piericidin P (**5**) (CD_3OD, 700MHz)

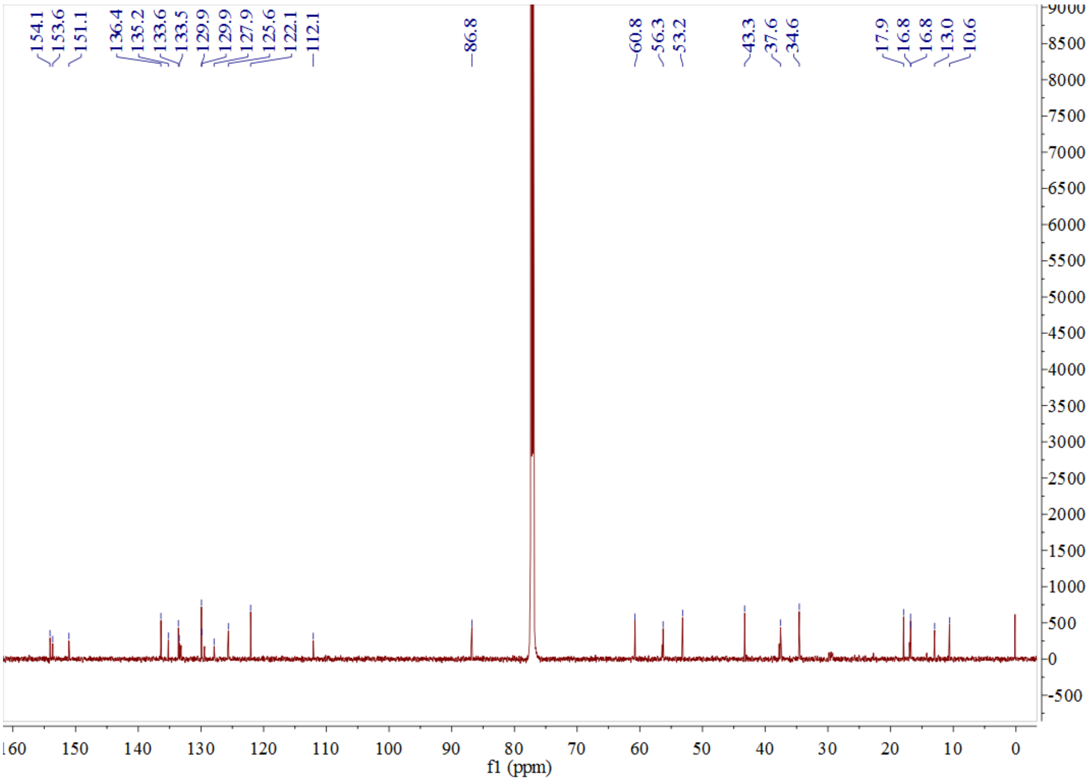

Figure S45. ^{13}C NMR spectrum of piericidin P (**5**) (CD_3OD, 175MHz)

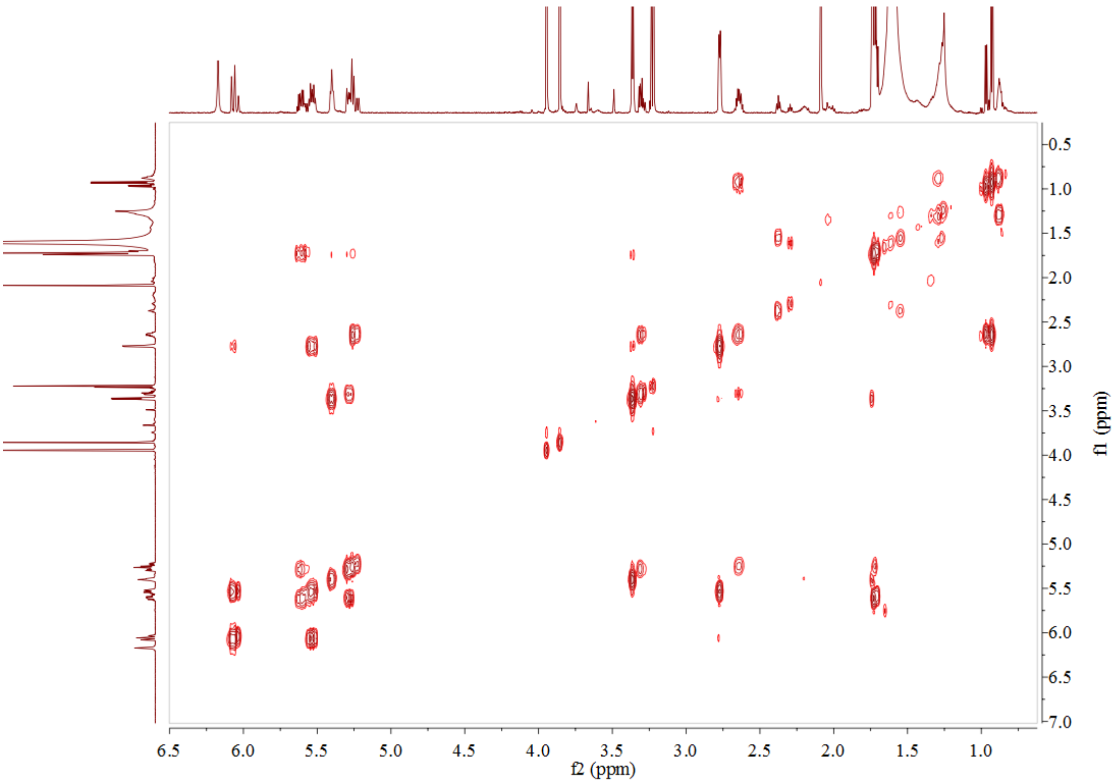

Figure S46. ^{1}H-^{1}H COSY spectrum of piericidin P (**5**) (CD_3OD)

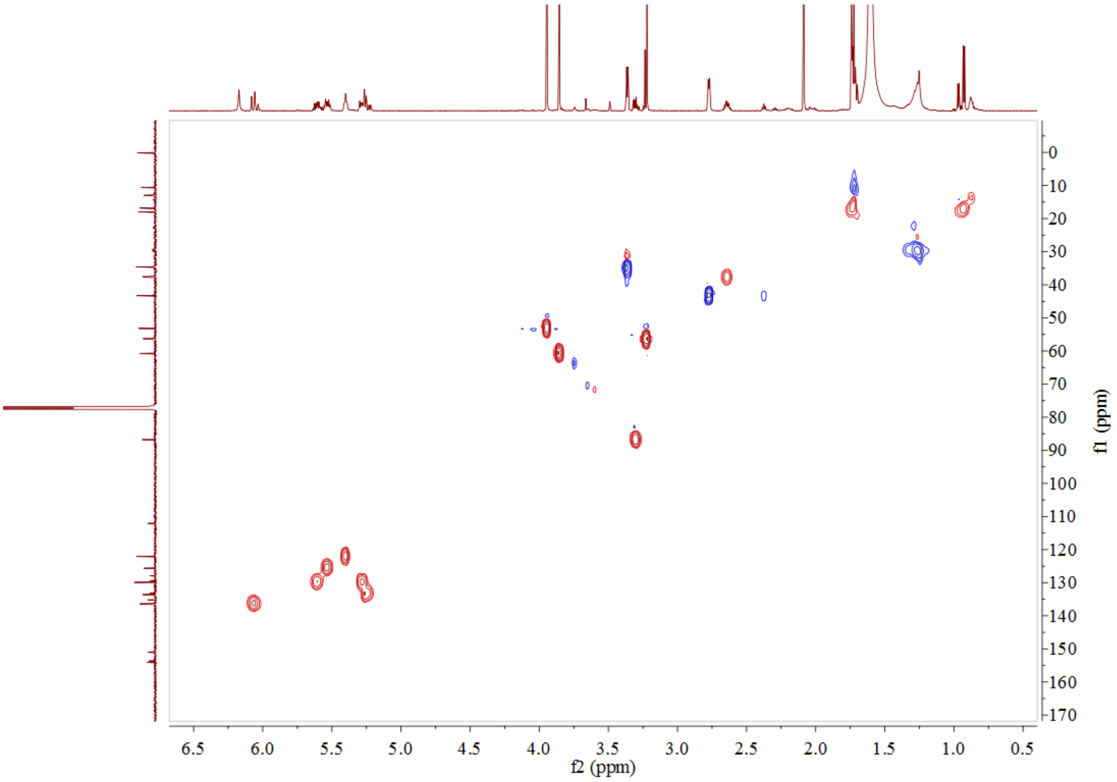

Figure S47. HSQC spectrum of piericidin P (**5**) (CD_3OD)

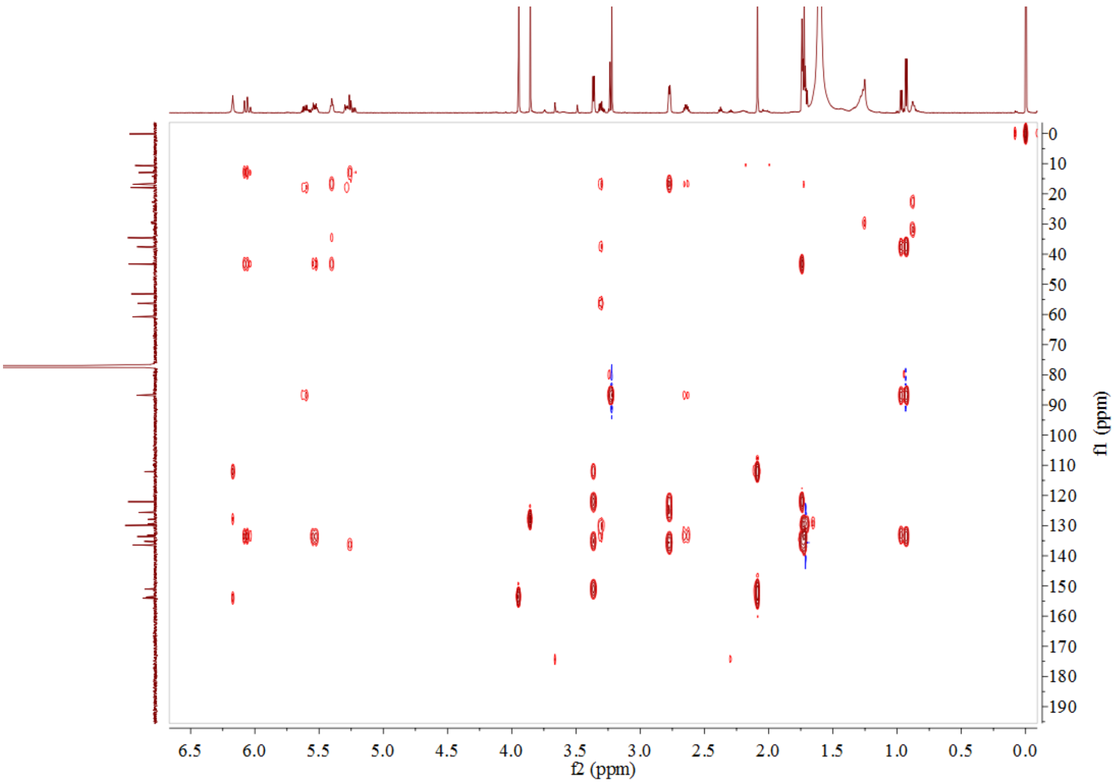

Figure S48. HMBC spectrum of piericidin P (**5**) (CD_3OD)

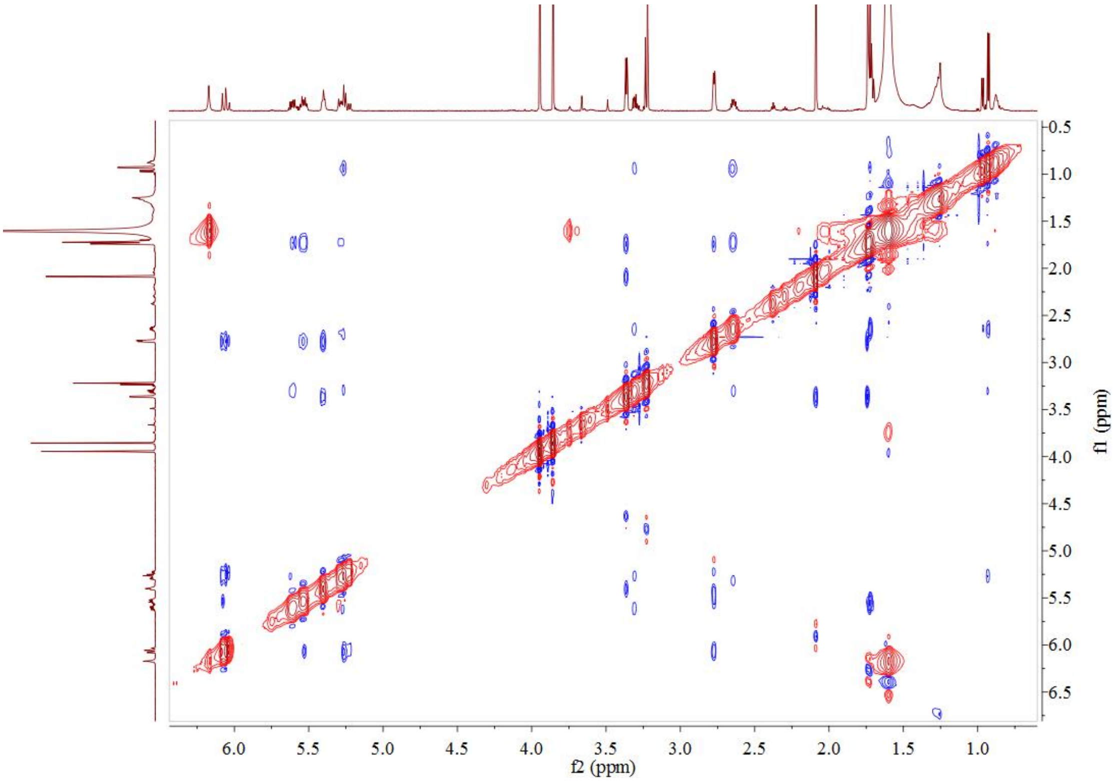

Figure S49. NOESY spectrum of piericidin P (**5**) (CD_3OD)

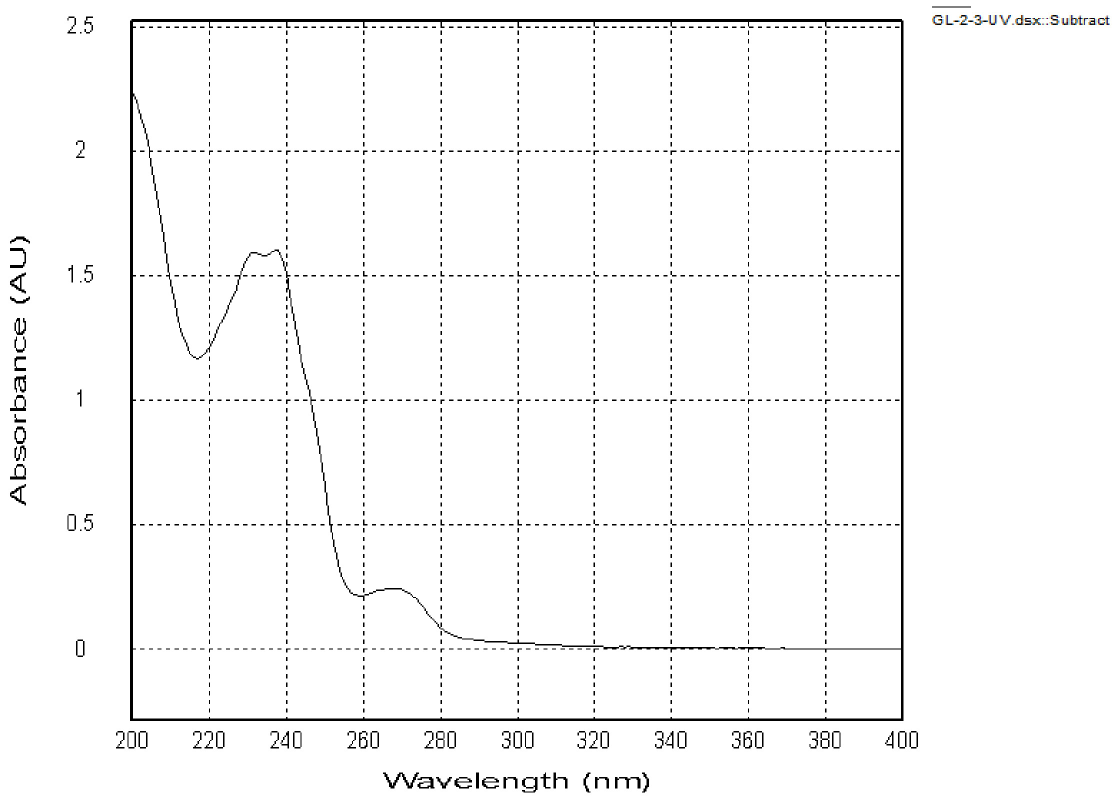

Figure S50. UV spectrum of piericidin P (**5**)

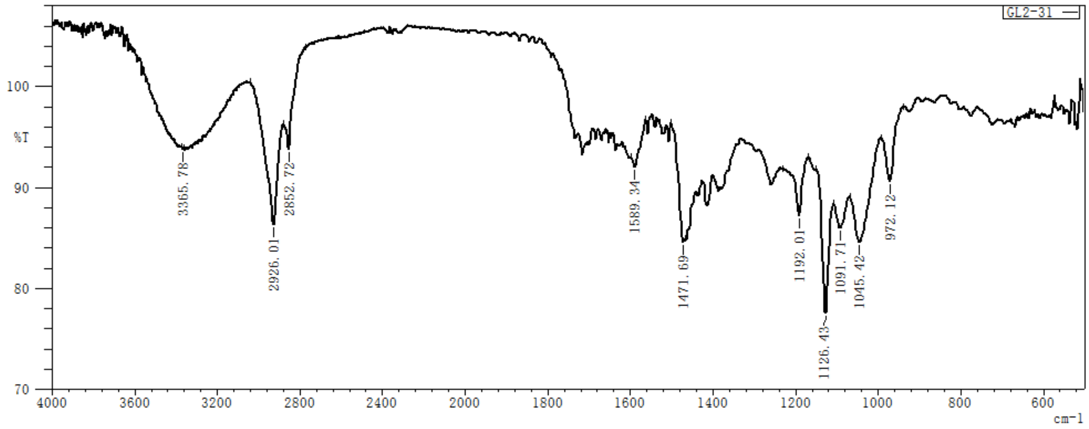

Figure S51. IR spectrum of piericidin P (**5**)

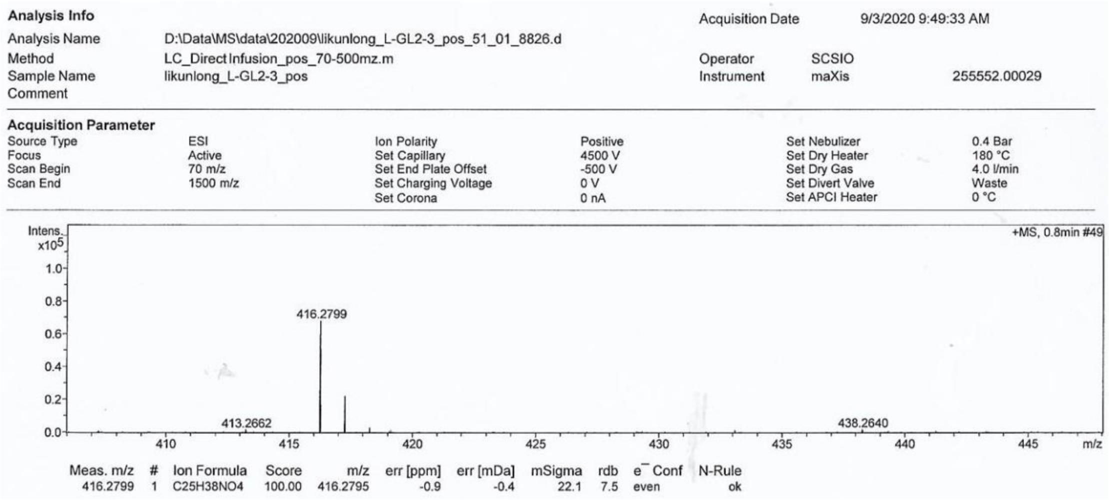

Figure S52. HRESIMS spectrum of piericidin (**5**)

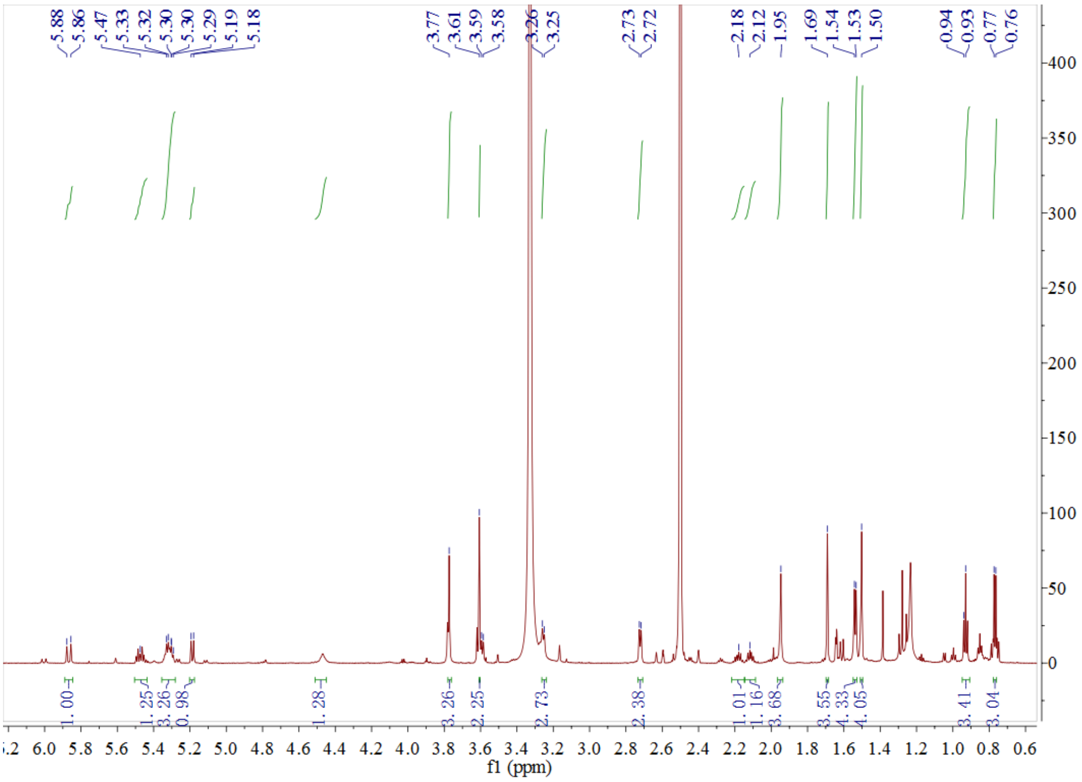

Figure S53. ^{1}H NMR spectrum of piericidin Q (**6**) (DMSO-d_6, 700MHz)

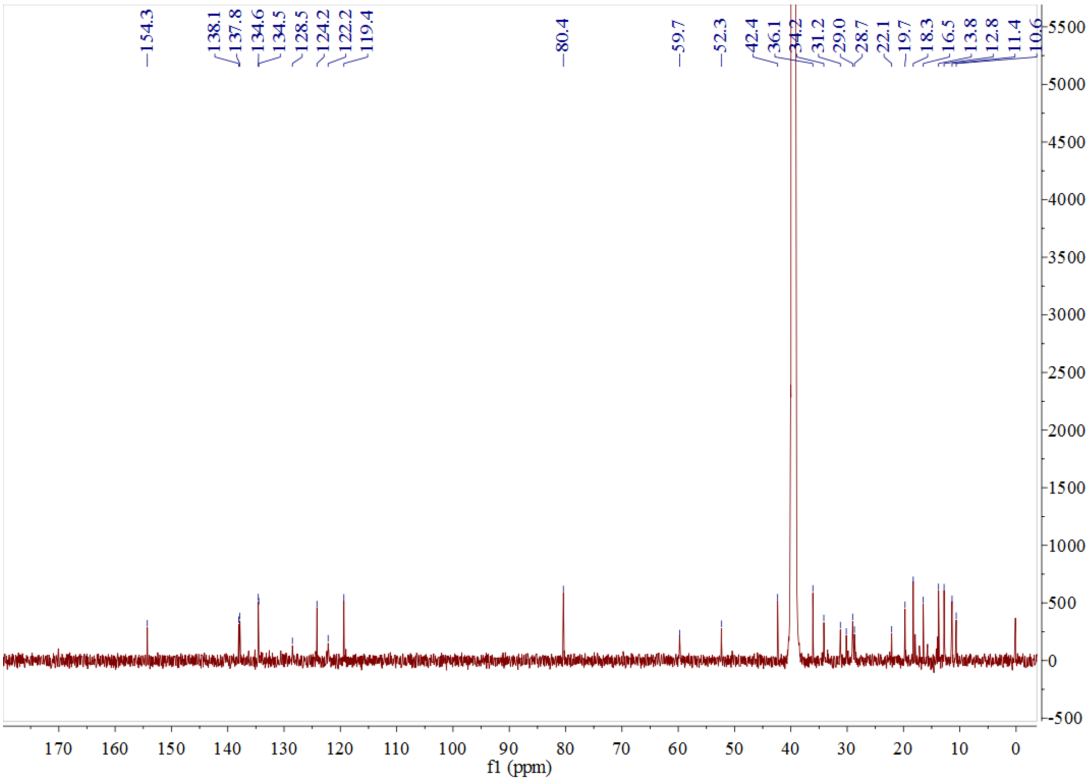

Figure S54. ^{13}C NMR spectrum of piericidin Q (**6**) (DMSO-d_6, 175MHz)

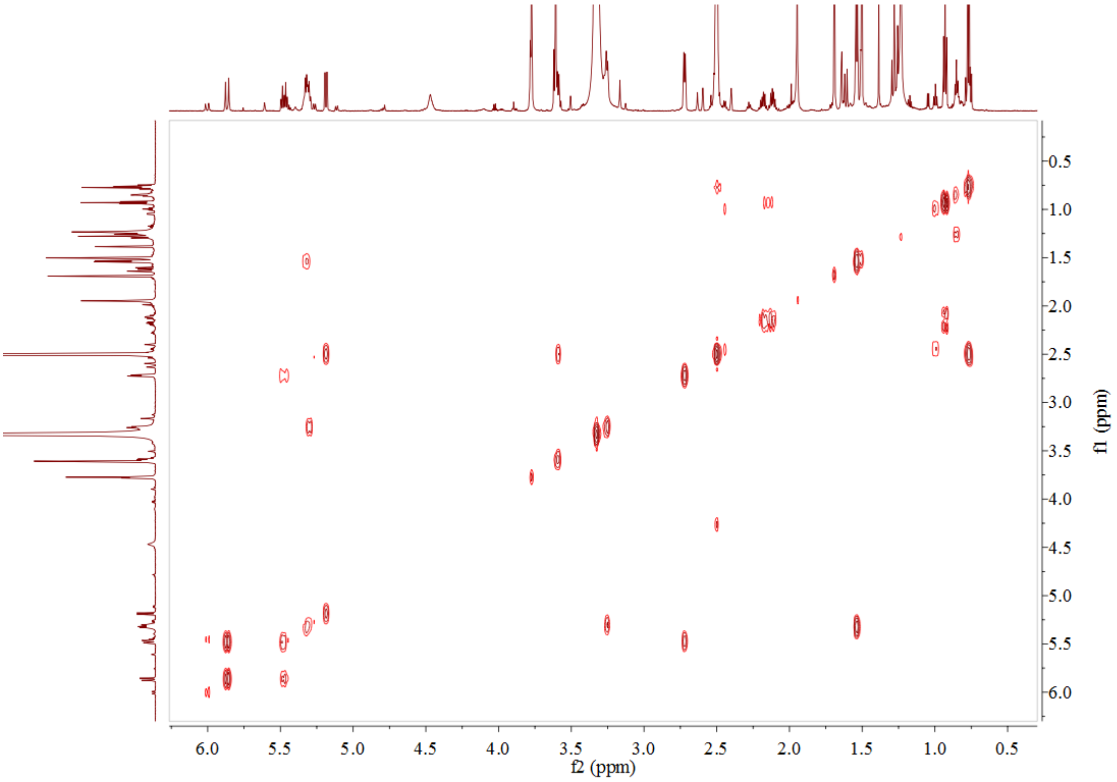

Figure S55. ^{1}H-^{1}H COSY spectrum of piericidin Q (**6**) (DMSO-d_6)

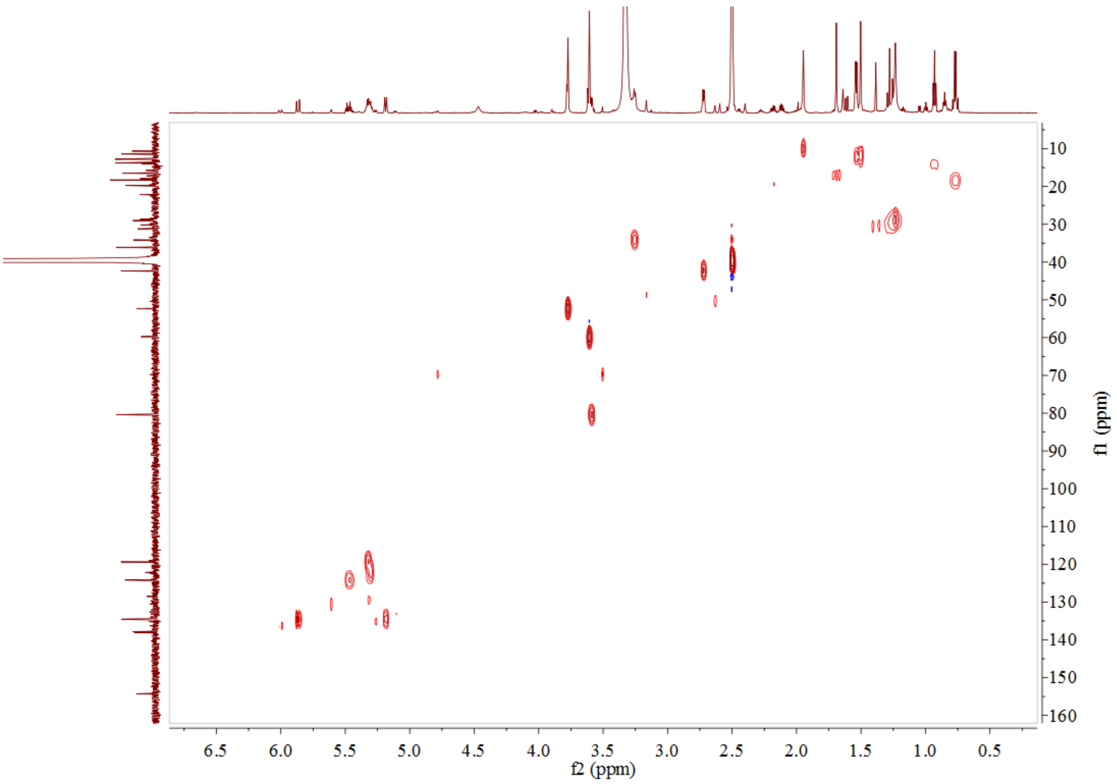

Figure S56. HSQC spectrum of piericidin Q (**6**) (DMSO-d_6)

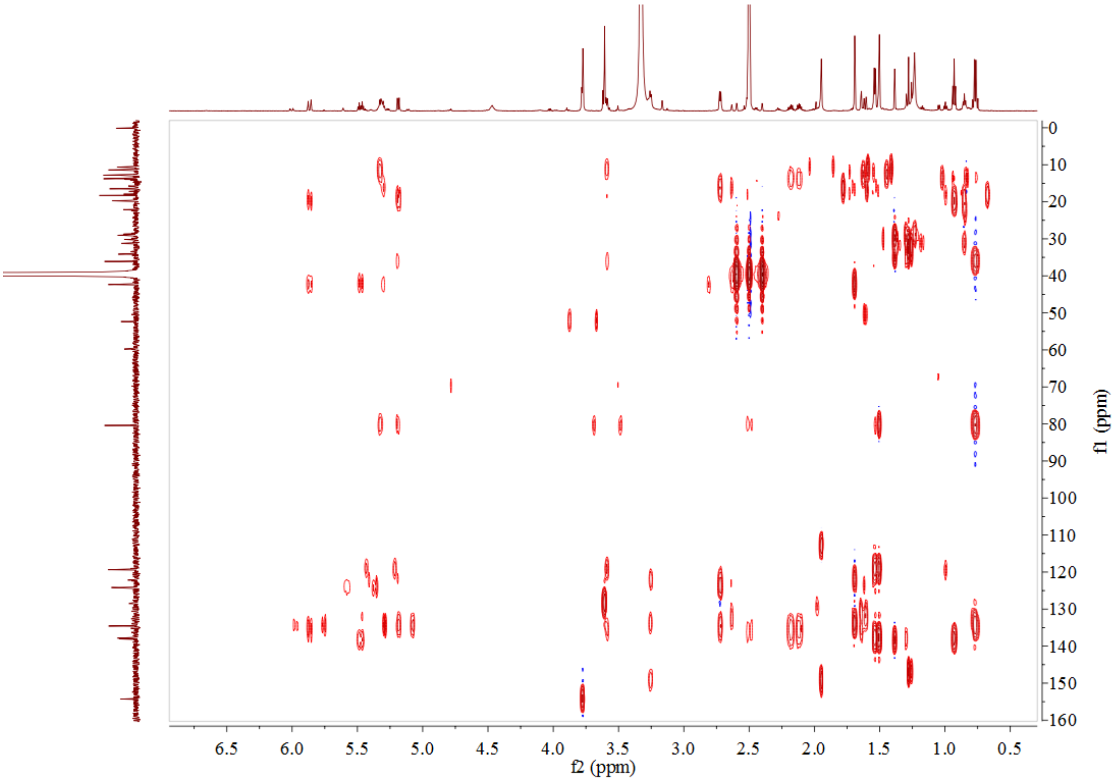

Figure S57. HMBC spectrum of piericidin Q (**6**) (DMSO-d_6)

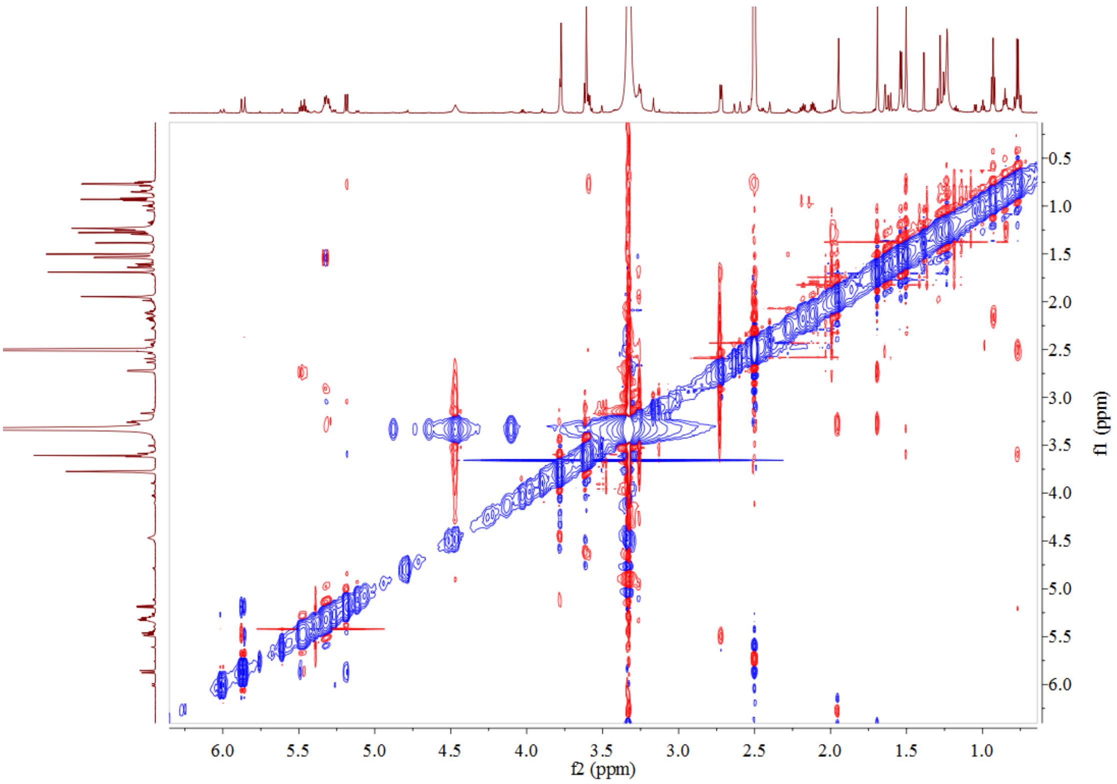

Figure S58. NOESY spectrum of piericidin Q (**6**) (DMSO-d_6)

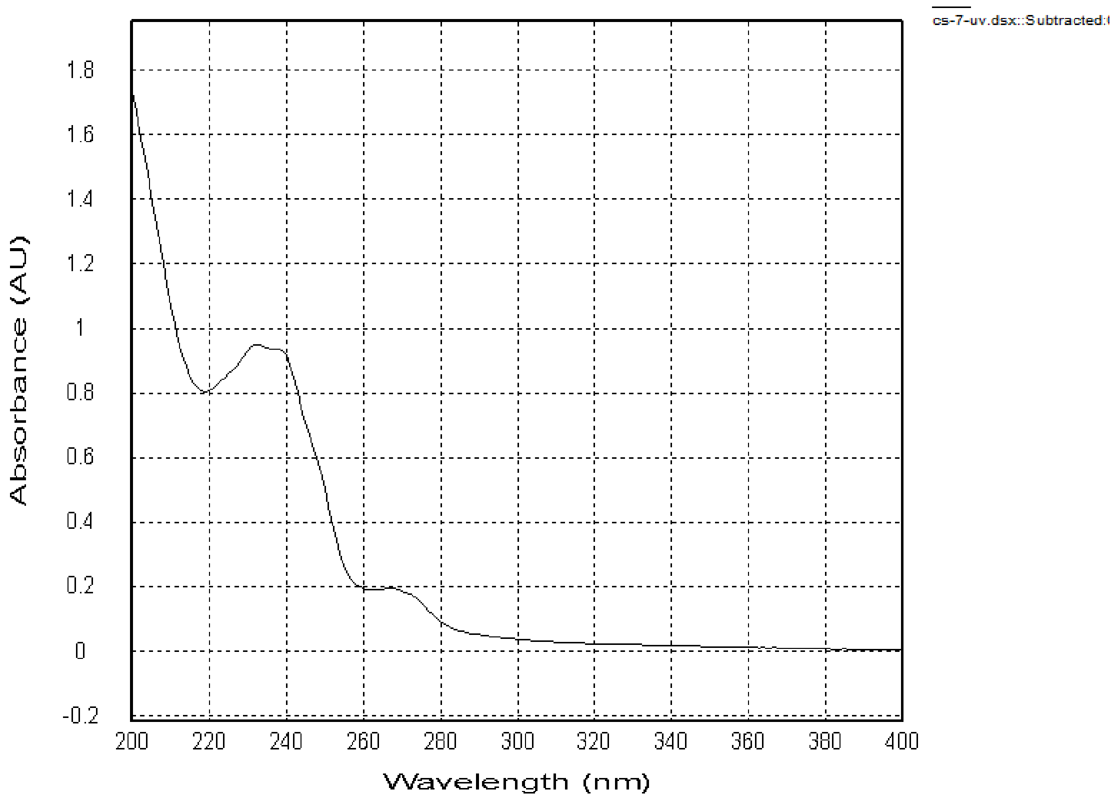

Figure S59. UV spectrum of piericidin Q (**6**)

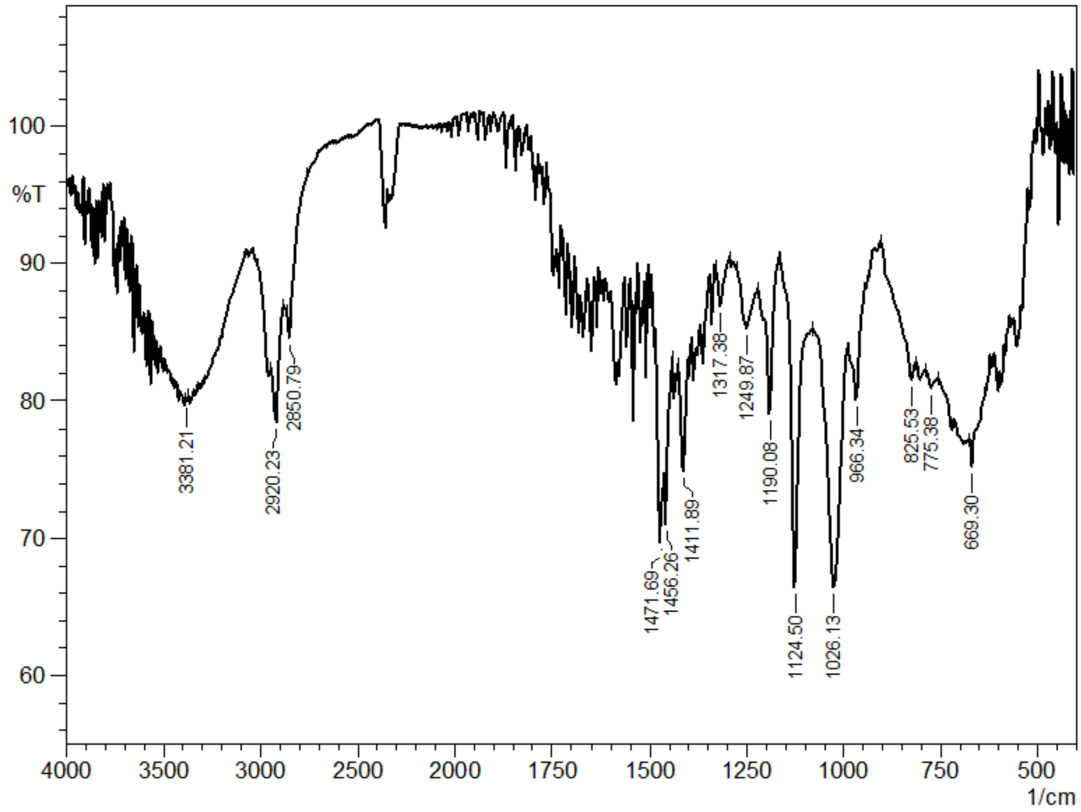

Figure S60. IR spectrum of piericidin Q (**6**)

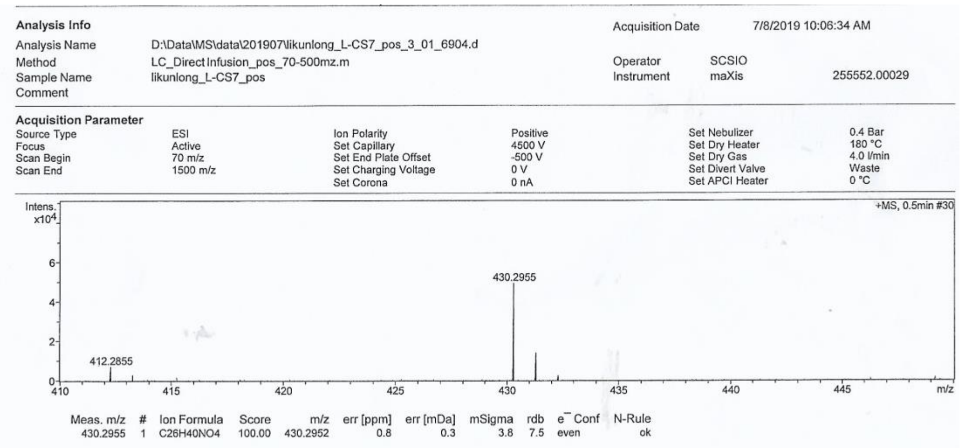

Figure S61. HRESIMS spectrum of piericidin Q (**6**)

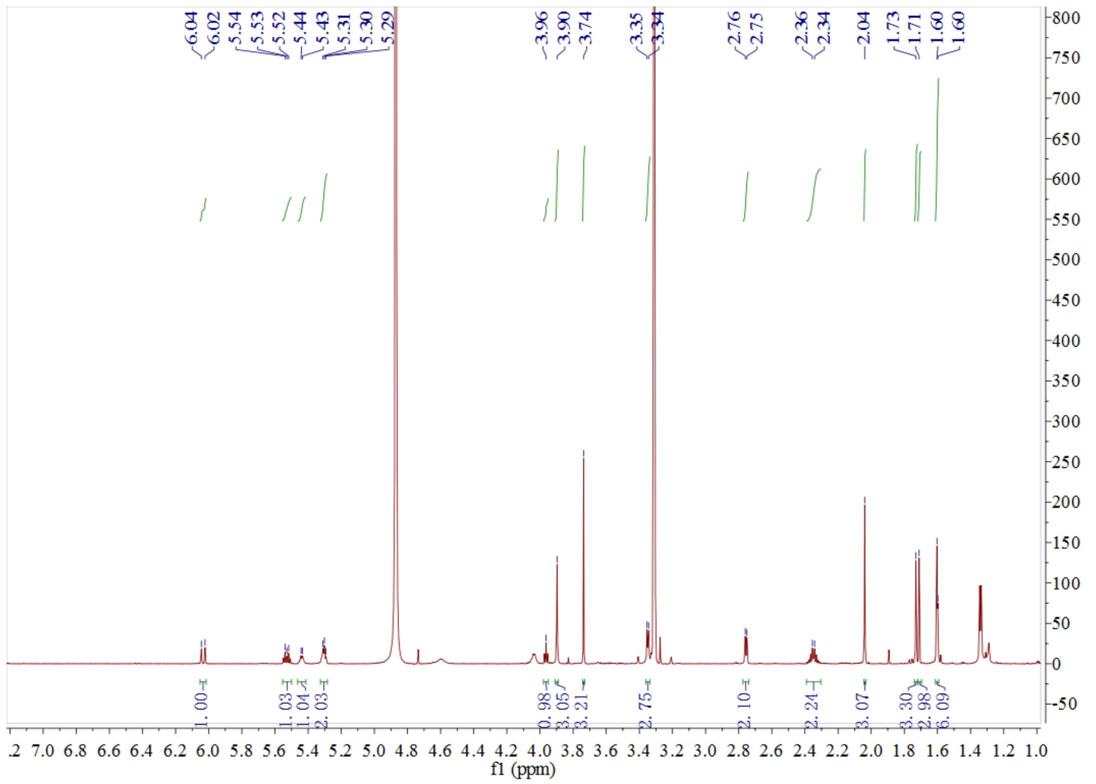

Figure S62. ^{1}H NMR spectrum of piericidin R (**7**) (CD_3OD, 700MHz)

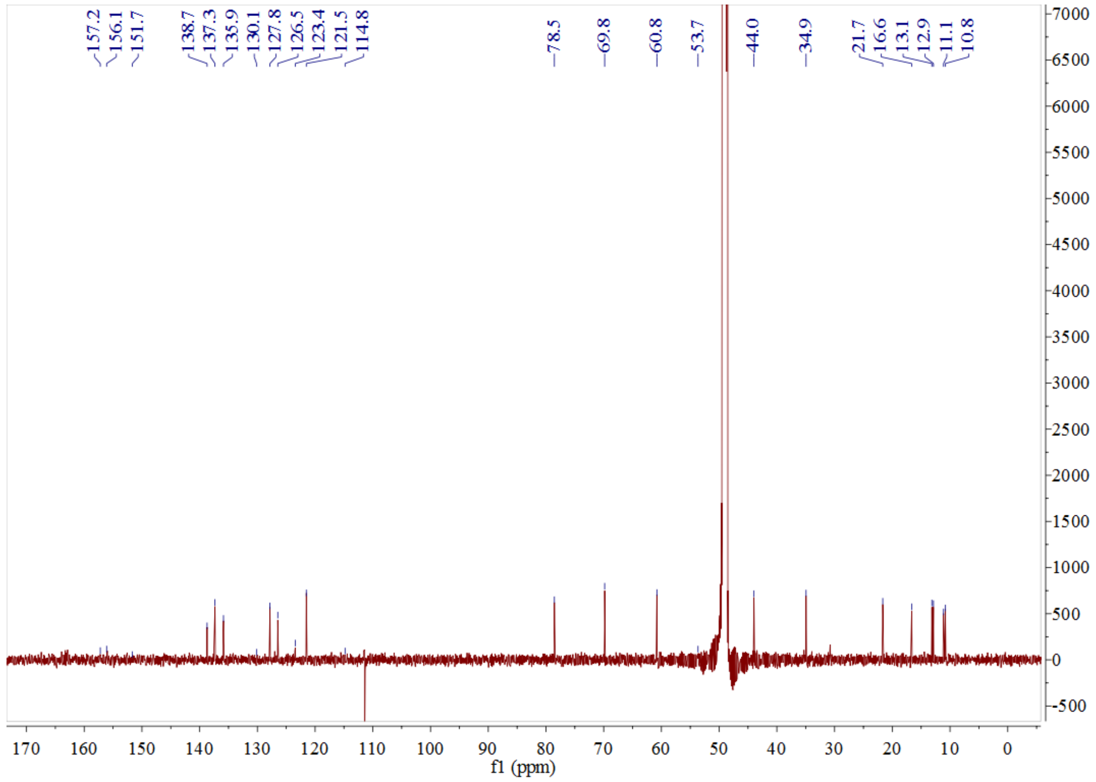

Figure S63. ^{13}C NMR spectrum of piericidin R (**7**) (CD_3OD, 175MHz)

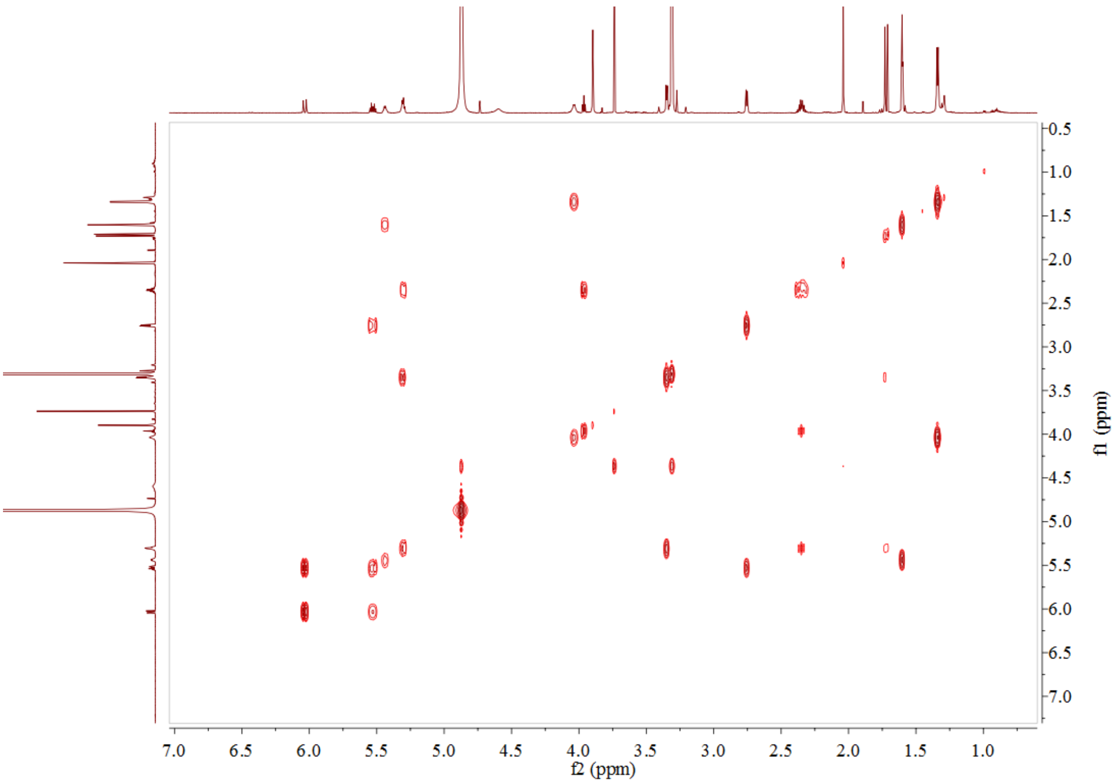

Figure S64. ^{1}H-^{1}H COSY spectrum of piericidin R (**7**) (CD_3OD)

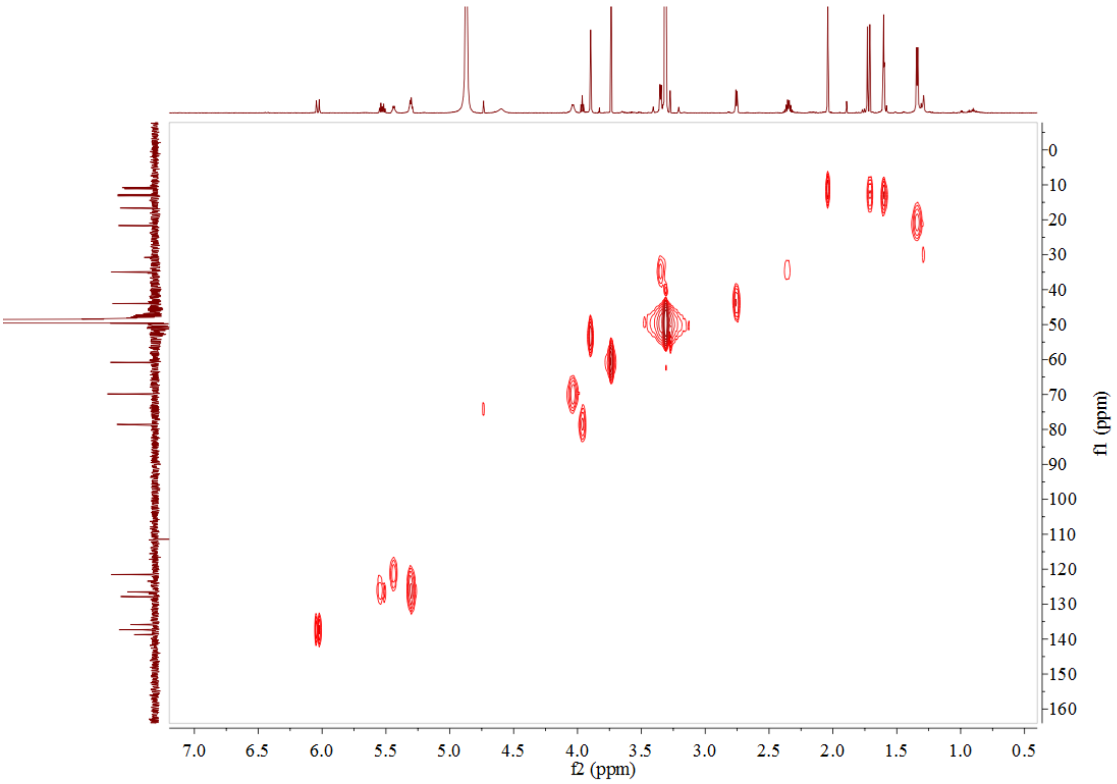

Figure S65. HSQC spectrum of piericidin R (**7**) (CD_3OD)

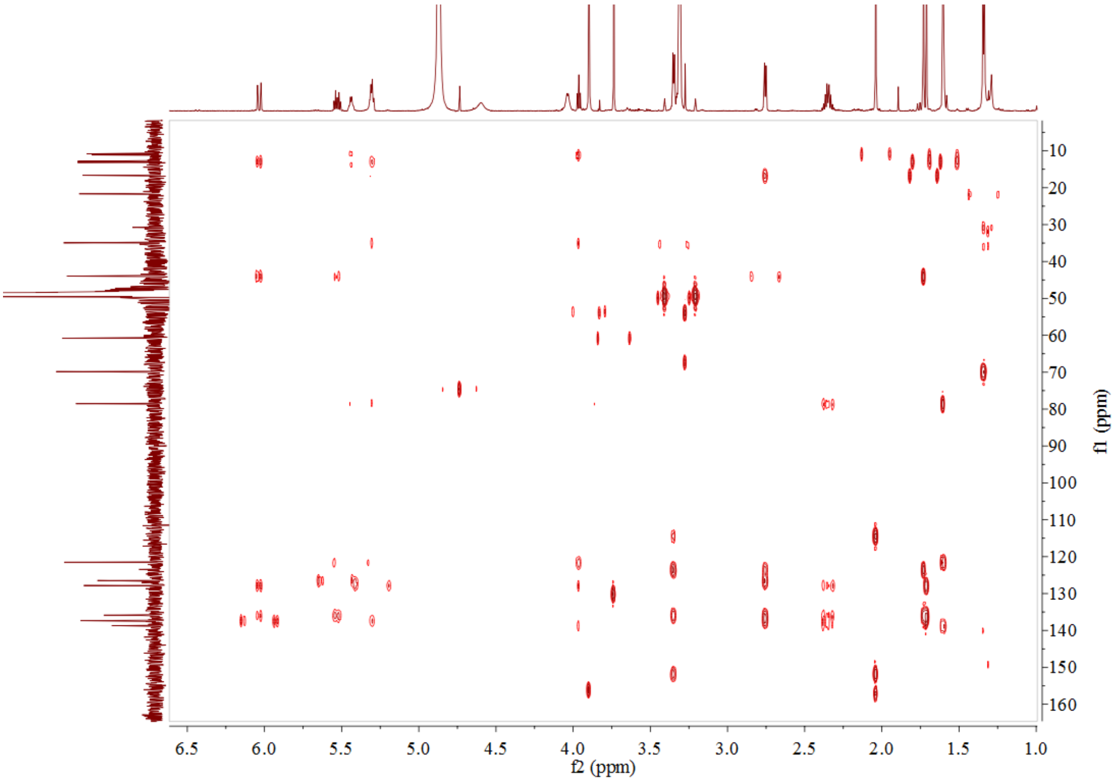

Figure S66. HMBC spectrum of piericidin R (**7**) (CD_3OD)

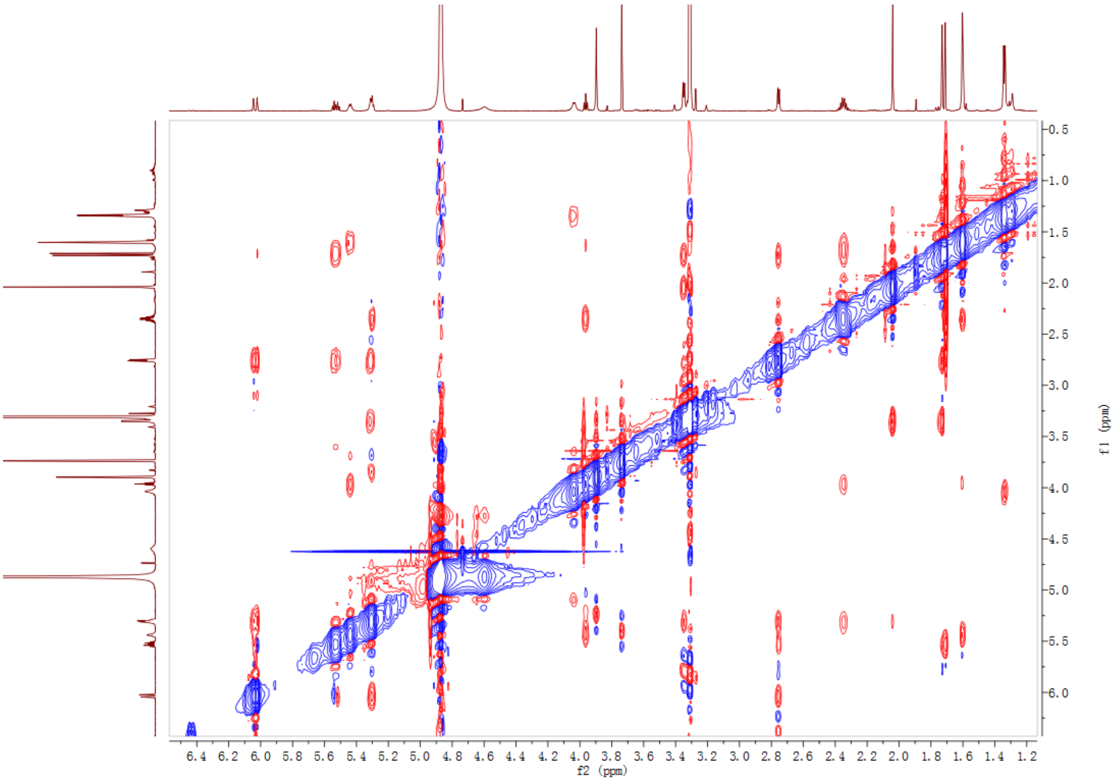

Figure S67. NOESY spectrum of piericidin R (**7**) (CD_3OD)

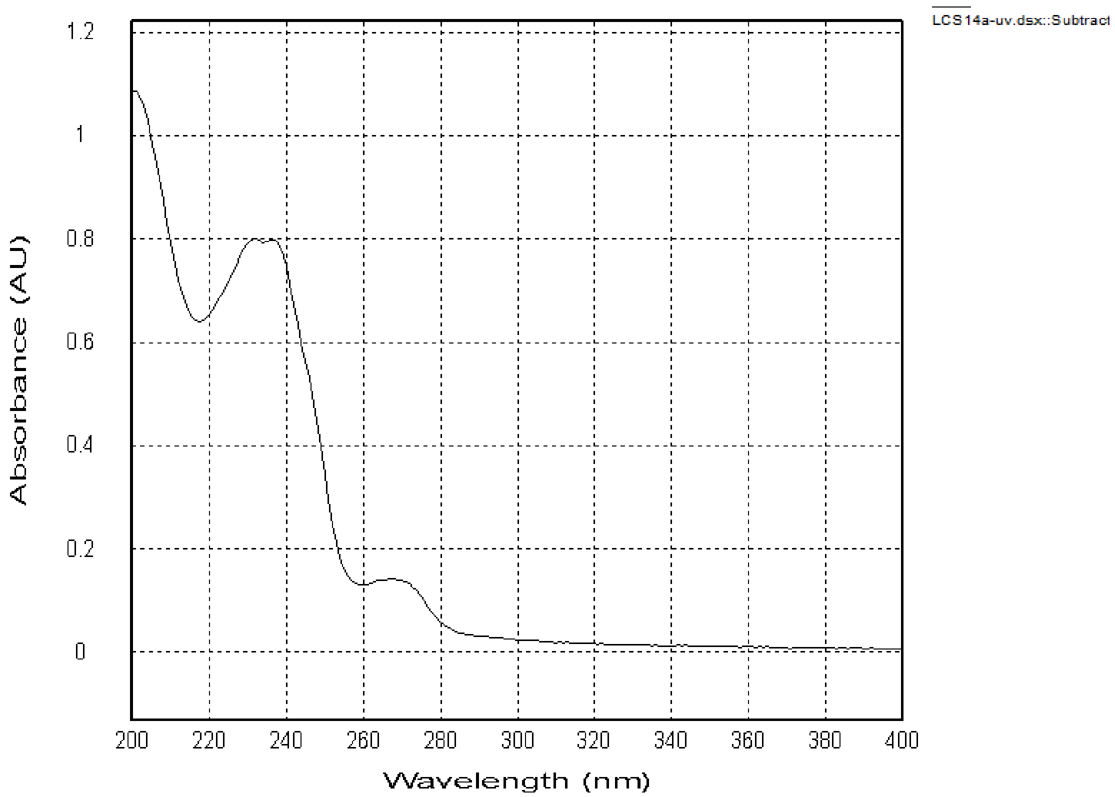

Figure S68. UV spectrum of piericidin R (**7**)

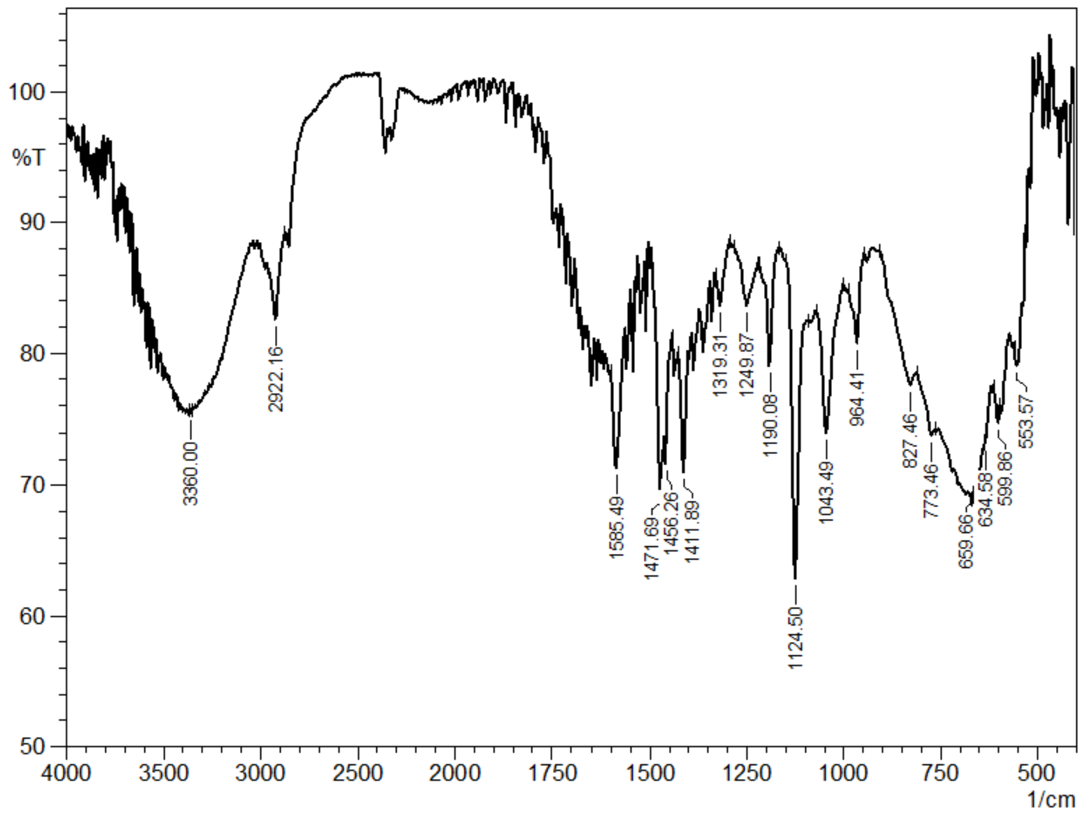

Figure S69. IR spectrum of piericidin R (**7**)

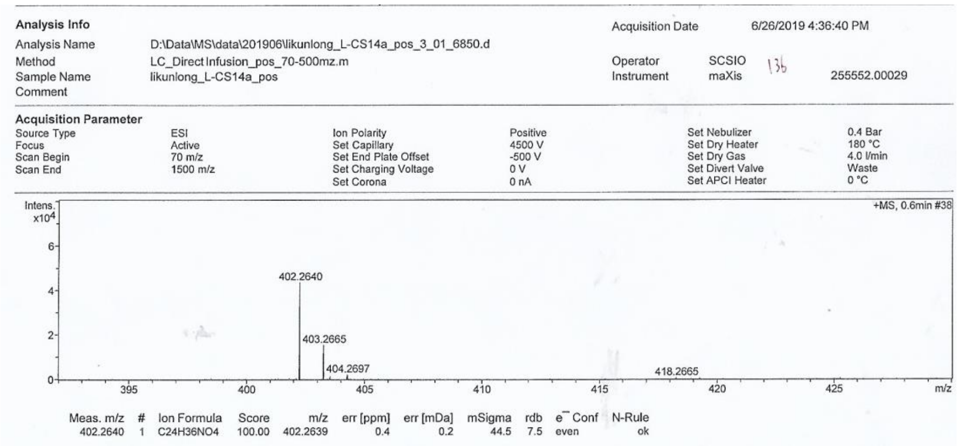

Figure S70. HRESIMS spectrum of piericidin R (**7**)

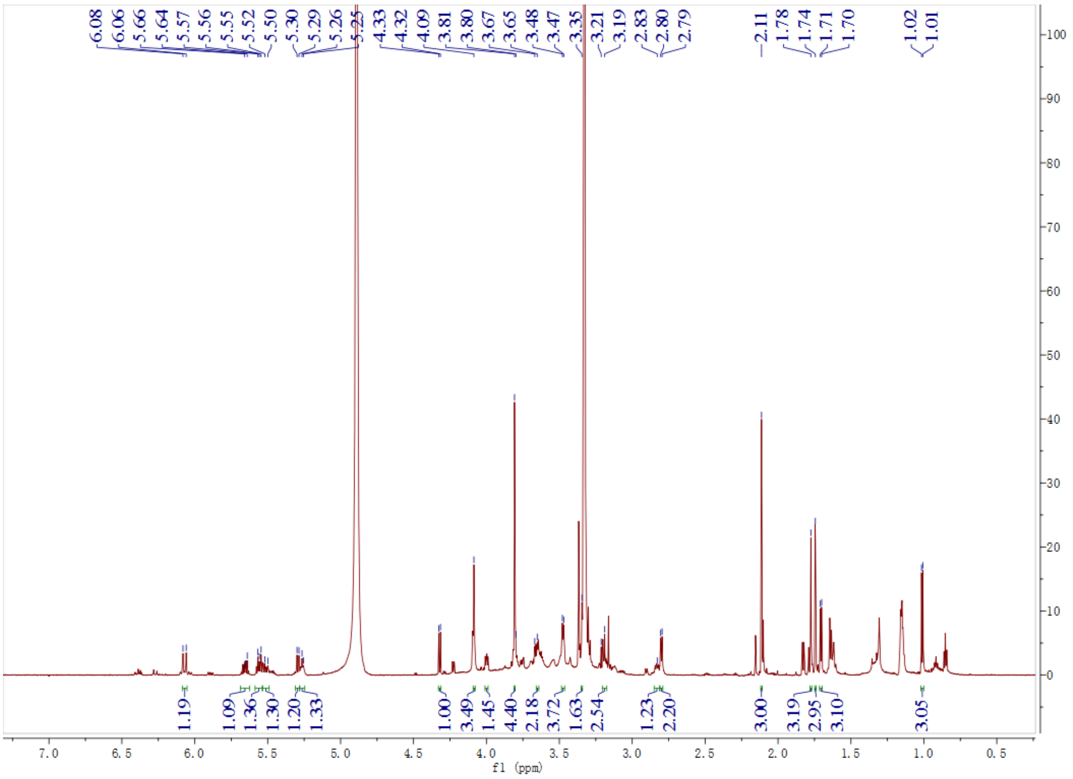

Figure S71. ^{1}H NMR spectrum of compound **8** (CD_3OD, 700MHz)

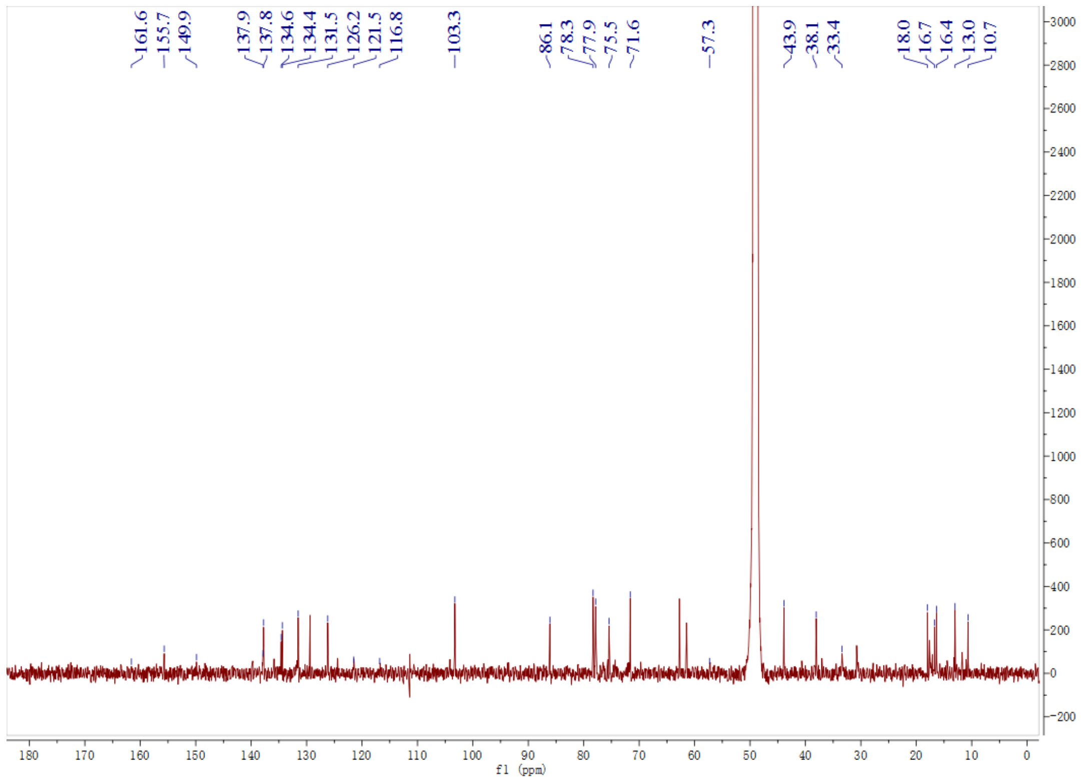

Figure S72. ^{13}C NMR spectrum of compound **8** (CD_3OD, 175MHz)

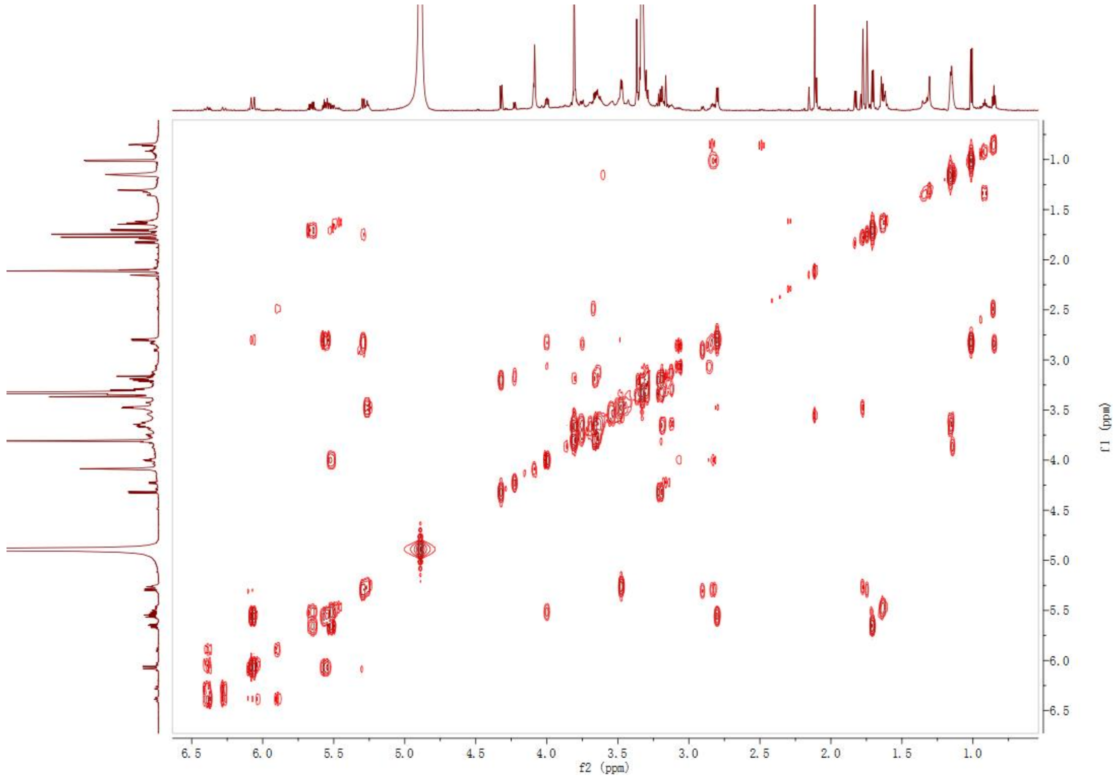

Figure S73. ^{1}H-^{1}H COSY spectrum of compound **8** (CD_3OD)

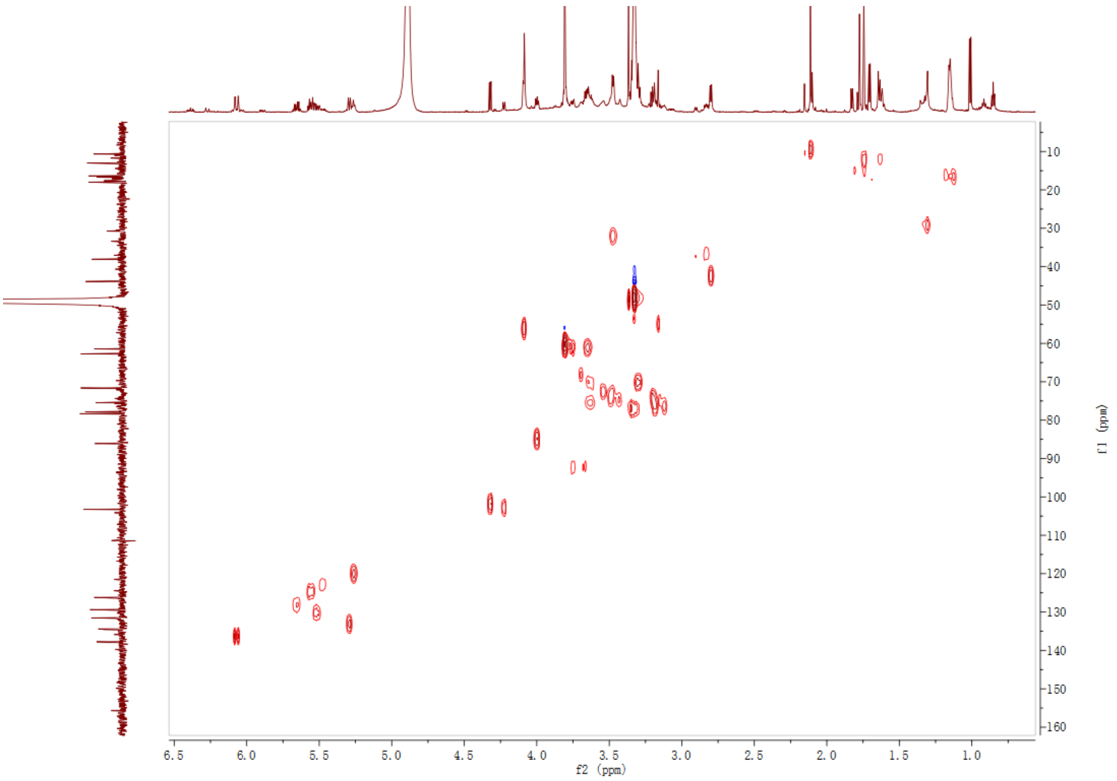

Figure S74. HSQC spectrum of compound **8** (CD_3OD)

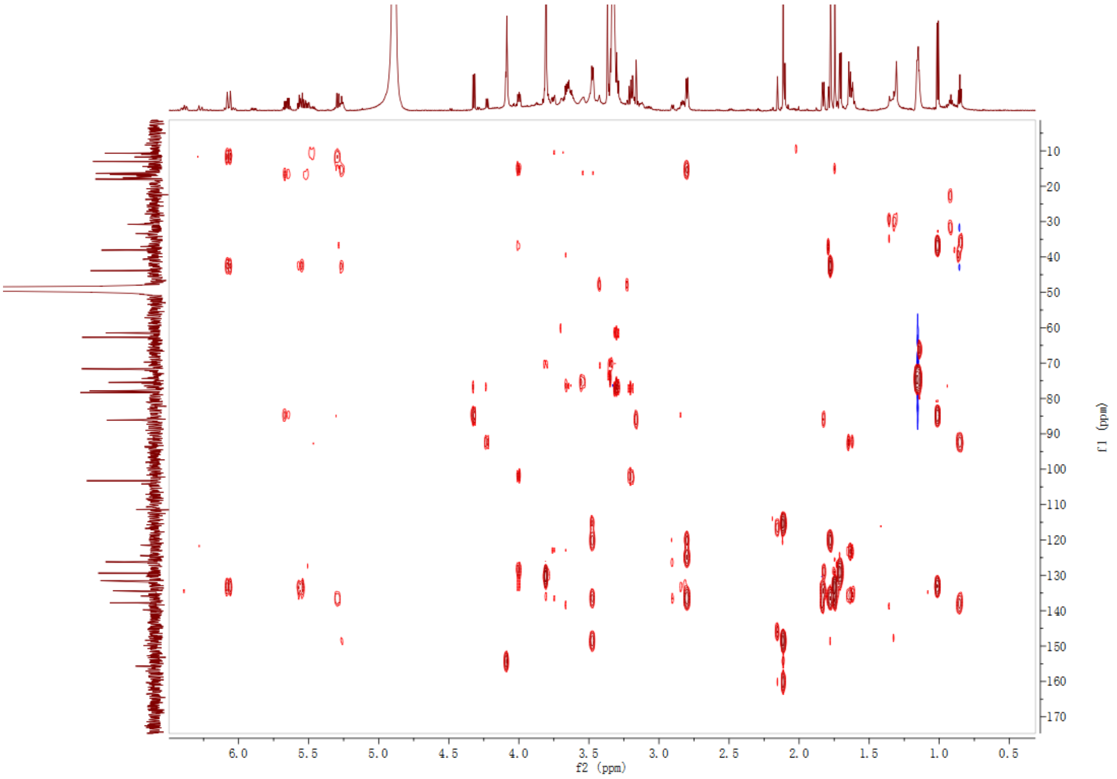

Figure S75 HMBC spectrum of compound **8** (CD_3OD)

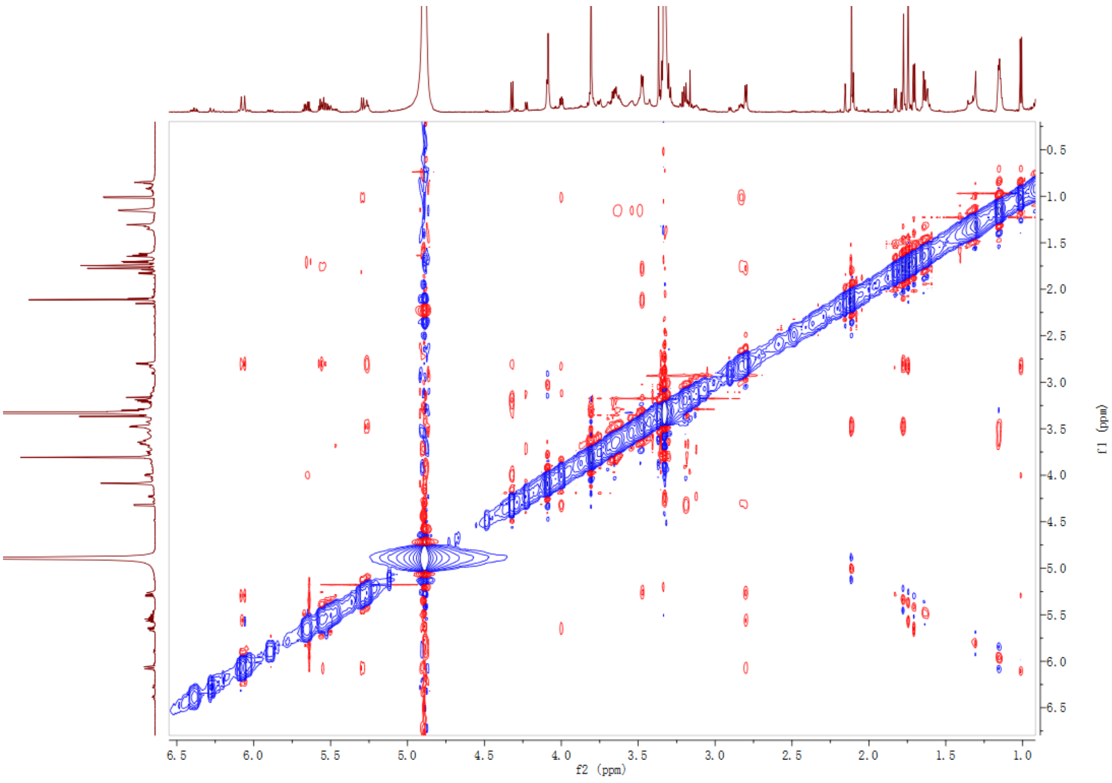

Figure S76. NOESY spectrum of compound **8** (CD_3OD)

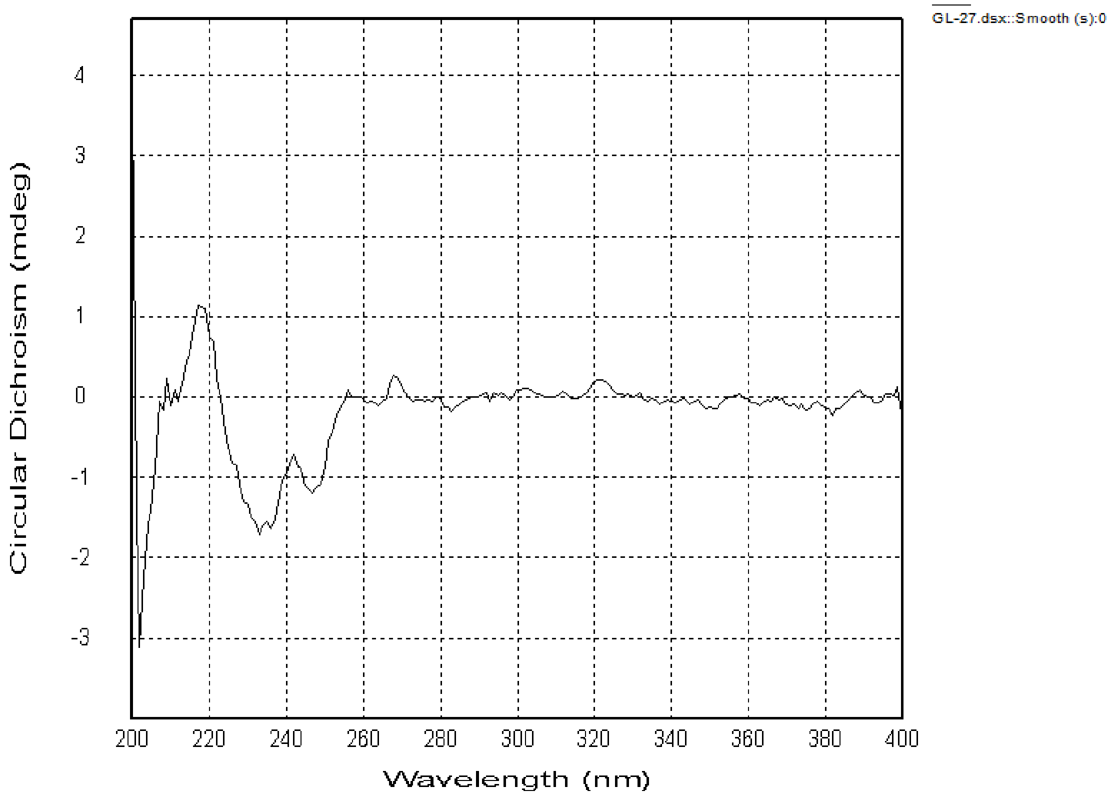

Figure S77. UV spectrum of compound **8**

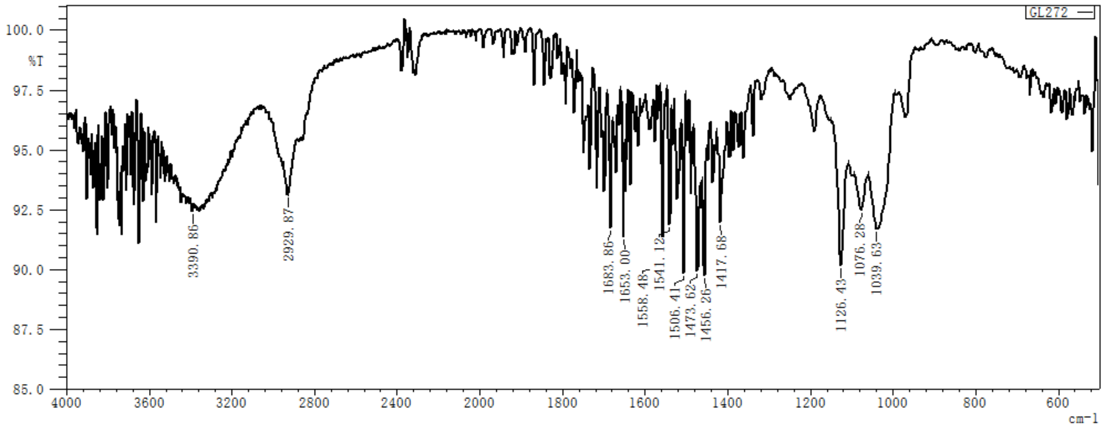

Figure S78. IR spectrum of compound **8**

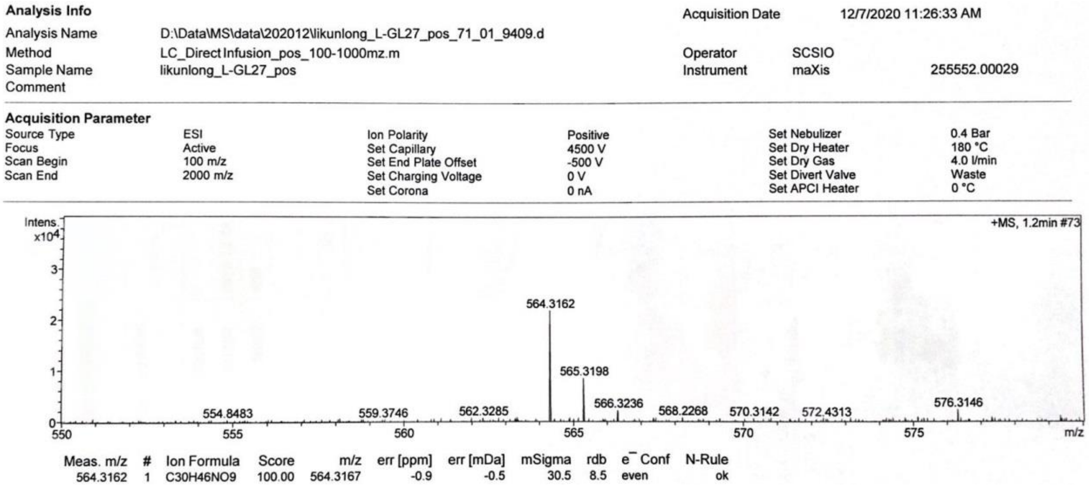

Meas. m/z	#	Ion Formula	Score	m/z	err [ppm]	err [mDa]	mSigma	rdb	e⁻ Conf	N-Rule
564.3162	1	C30H46NO9	100.00	564.3167	-0.9	-0.5	30.5	8.5	even	ok

Figure S79. HRESIMS spectrum of compound **8**

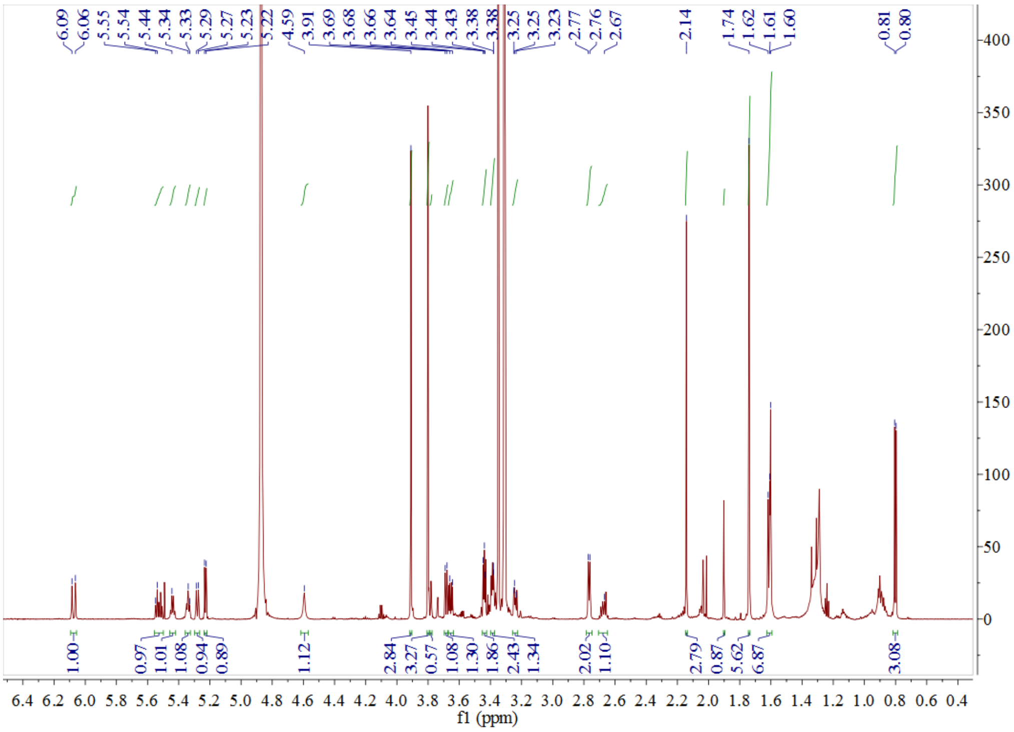

Figure S80. ^{1}H NMR spectrum of compound **9** (CD_3OD, 700MHz)

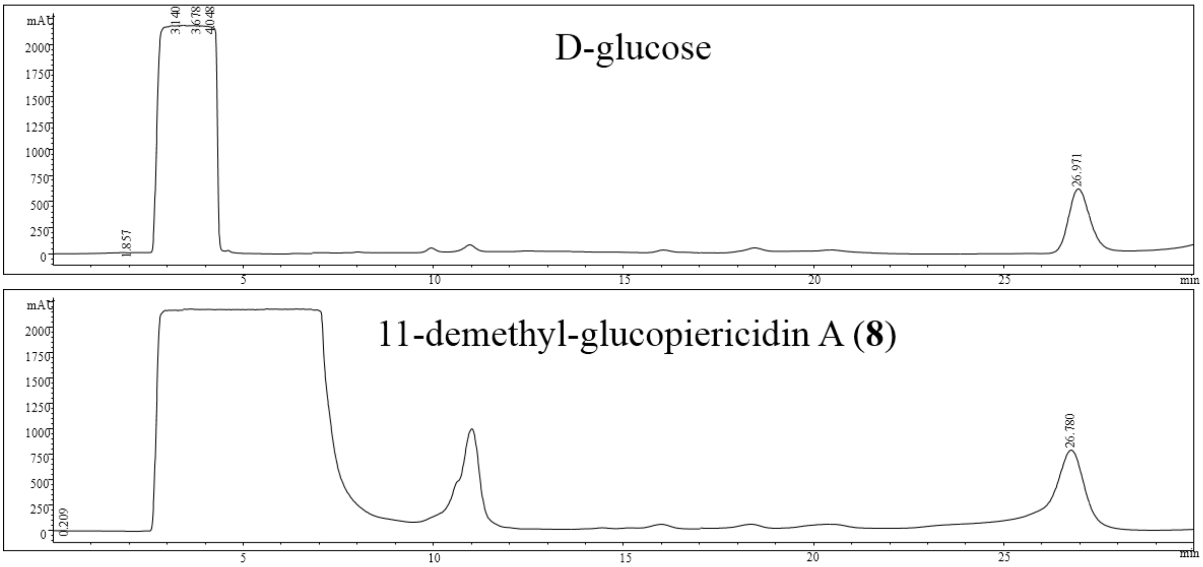

Figure S81. The HPLC results for D-glucose and **8** by acidic hydrolysis

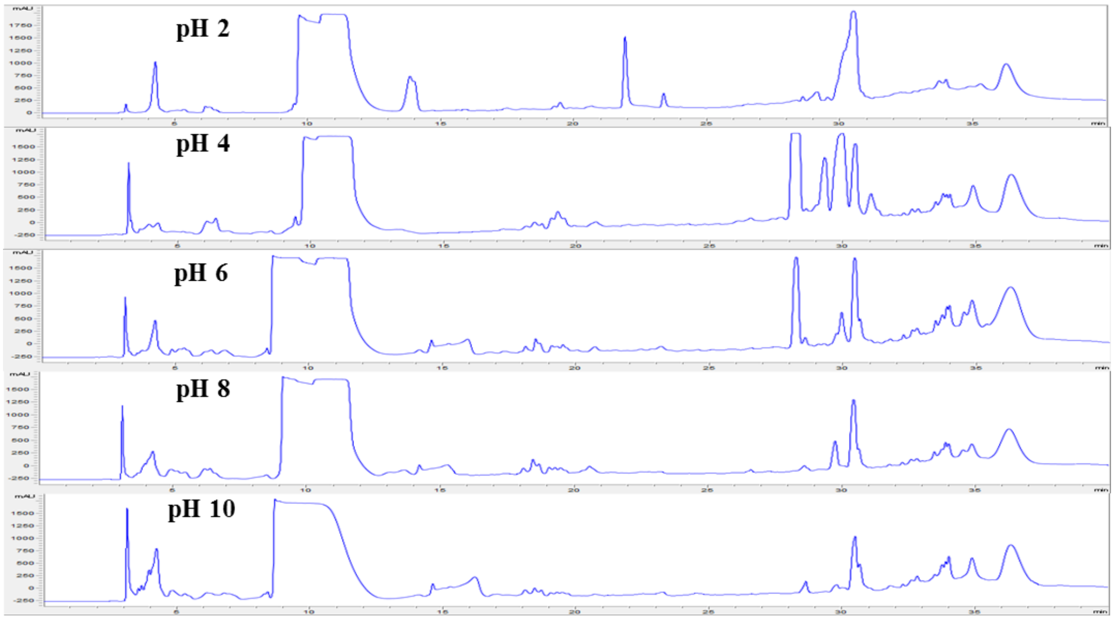

Figure S82. The HPLC analysis of crude extract in different external pH.

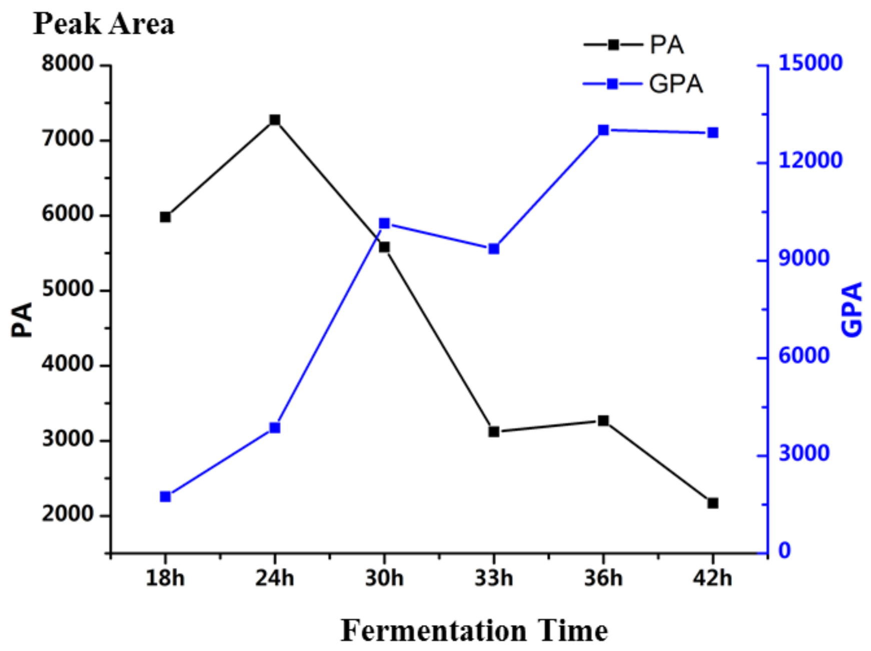

Figure S83. The content analysis of PA and GPA in different fermentation time.

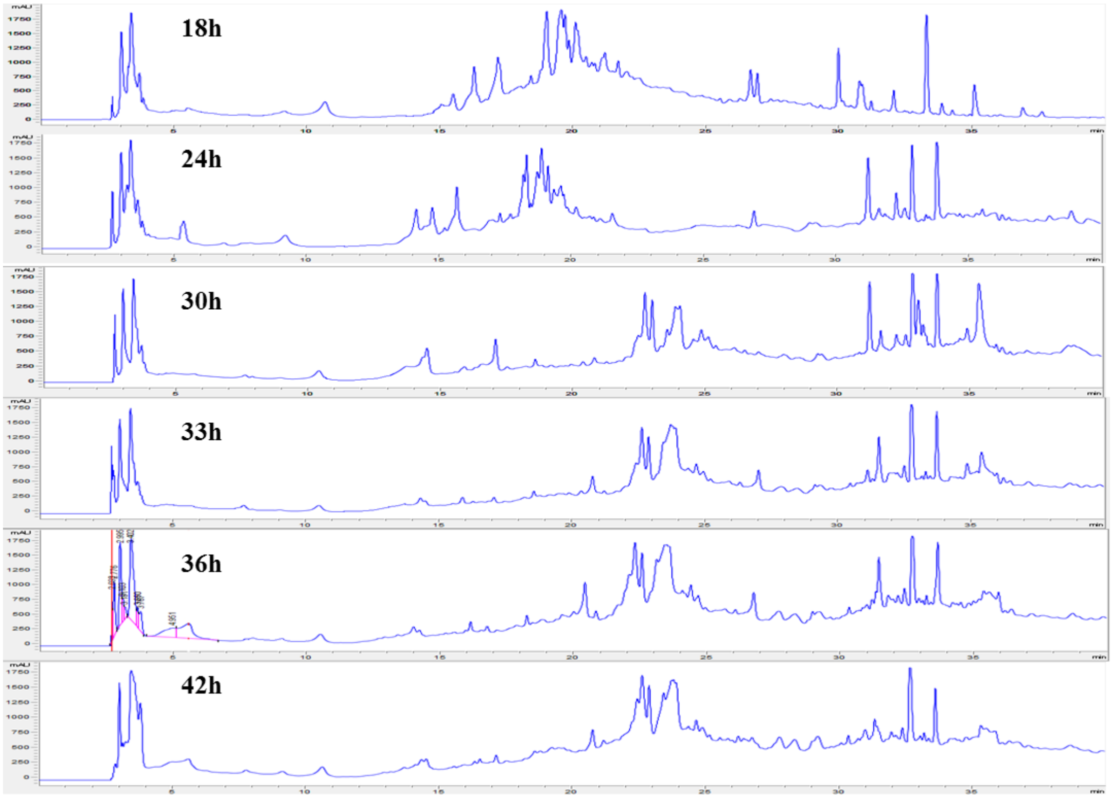

Figure S84. The HPLC analysis of crude extract in different fermentation time.
